# Treatment- and immune-related adverse events of immune checkpoint inhibitors in esophageal or gastroesophageal junction cancer: A network meta-analysis of randomized controlled trials

**DOI:** 10.3389/fonc.2022.821626

**Published:** 2022-12-08

**Authors:** Jianqing Zheng, Bifen Huang, Lihua Xiao, Min Wu, Jiancheng Li

**Affiliations:** ^1^ Department of Radiation Oncology, The Second Affiliated Hospital of Fujian Medical University, Quanzhou, China; ^2^ Department of Obstetrics and Gynecology, Quanzhou Medical College People’s Hospital Affiliated, Quanzhou, China; ^3^ Department of Radiation Oncology, Clinical Oncology School of Fujian Medical University, Fujian Cancer Hospital, Fuzhou, China

**Keywords:** immune checkpoint inhibitors, esophageal cancer, gastro-esophageal junction cancer, network meta-analysis, safety assessment

## Abstract

**Objective:**

To systematically evaluate the safety and adverse event profiles of immune checkpoint inhibitors (ICIs) in patients with esophageal cancer (EPC) or gastroesophageal junction cancer (GEJC).

**Methods:**

PubMed, Web of Science, Cochrane Library, and major conference proceedings were systematically searched for all phase II or phase III randomized controlled trials (RCTs) in EPC or GEJC using ICIs. Safety outcomes including treatment-related adverse events (trAEs), immune-related adverse events (irAEs), and serious trAEs were evaluated by network meta-analysis or dichotomous meta-analysis based on the random-effects model.

**Results:**

Eleven RCTs involving EPC (five RCTs) and GEJC (six RCTs) were included in the final meta-analysis. NMA showed that placebo was associated with the best safety ranking for grade 3–5 trAEs (SUCRA = 96.0%), followed by avelumab (78.6%), nivolumab (73.9%), ipilimumab (57.0%), and pembrolizumab (56.6%). Conventional pairwise meta-analysis (CPM) showed that ICIs have similar grade 3–5 trAE risk compared with chemotherapy (RR = 0.764, 95% CI: 0.574 to 1.016, *I* ^2^ = 95.7%, *Z* = 1.85, *P* = 0.065). NMA showed that the general safety of grade 3–5 irAEs ranked from high to low is as follows: ChT (85.1%), placebo (76.5%), ipilimumab (56.0%), nivolumab (48.5%), avelumab (48.4%), camrelizumab (41.8%), pembrolizumab (36.4%), and nivolumab + ipilimumab (21.6%). CPM showed that the rates of grade 3–5 irAEs in the ICI group and the chemotherapy group were 7.35% (154/2,095, 95% CI: [6.23%, 8.47%]) versus 2.25% (42/1,869, 95% CI: [1.58%, 2.92%]), with statistical significance (RR = 3.151, 95% CI = 2.175 to 4.563, *Z* = 6.07, *P* = 0.000). The most common irAEs in the ICI group were skin reaction (15.76%, 95% CI: [13.67%, 17.84%]), followed by hypothyroidism (9.73%, 95% CI: [8.07%, 11.39%]), infusion-related reactions (5.93%, 95% CI: [4.29%, 7.58%]), hepatitis (5.25%, 95% CI: [4.28%, 6.22%]), and pneumonitis (4.45%, 95% CI: [3.5%, 5.4%]).

**Conclusion:**

Different ICIs had different toxicity manifestations and should not be considered as an entity. Compared with chemotherapy, ICIs were more prone to irAEs, but the overall rates remained low and acceptable. For clinicians, it is important to recognize and monitor the adverse events caused by ICIs for patients with EPC or GEJC.

## Introduction

Worldwide, esophageal cancer (EPC) still remains one of the most commonly diagnosed cancers and the leading cause of cancer-related death (1), particularly with the highest rates and mortality occurring in China (2). According to the GLOBOCAN report, an estimated 604,100 new cases were diagnosed in 2020 globally, among which Chinese cases accounted for 53.5%, which was up to 324,000 cases (3). An estimated 544,000 new deaths occurred in 2020 globally, while Chinese cases accounted for 55.3%, which was up to 301,000 cases (3). Patients with EPC are most commonly diagnosed with locally advanced cancer stages, and more than 50.4% of cases suffered from distant metastases and irreversible diseases at the time of diagnosis, which led to a frustrating overall 5-year survival rate of less than 20% (4). Generally, fluoropyrimidine and platinum-based regimens are recommended and accepted as standard first-line treatment regimen (5). Although chemotherapy has improved the overall 5-year survival rate to a certain extent, the prognosis of esophageal cancer is still poor (6). In particular, after first-line chemotherapy, there is no accepted and satisfactory standard treatment for advanced or metastatic esophageal cancer (7).

In recent years, cancer immunotherapies based on immune checkpoint inhibitors (ICIs) have become the fifth largest tumor treatment after surgery, chemotherapy, radiotherapy, and small molecules targeted therapy in oncology and have revolutionized the treatment landscape and made major breakthroughs in the treatment of tumors, especially for advanced or metastatic cancer (8). Clinical applications or trials on ICIs had been carried out in the field of various types of tumors, and more and more cancer patients had benefited from this innovative treatment (9). Compared with that of the four existing traditional treatment regimens, the scope of application of cancer immunotherapies is appropriately enlarged, and the number of patients receiving immunotherapies is increasing (10). ICIs directly and selectively killed cancer cells through immunogenic cell death by activating the immune system of cancer patients (11). ICIs had improved the survival rate of many refractory tumors and the quality of life of patients with advanced cancer (12). However, the emergence of a new treatment model and drugs is also accompanied by the emergence of new medication regimens and adverse reactions (11). Although ICIs have shown significant clinical benefits in improving the survival prognosis for most cancer patients, immune-related adverse events (irAEs) that affect body organs are one of the major hindrances when these drugs are applied (13). Although a large number of studies have confirmed the efficacy and safety of ICIs in esophageal cancer, there is a lack of direct head-to-head comparison of evidence for different types of ICIs (9, 14). Therefore, it is not clear if different ICIs have different toxicity profiles in the immunotherapy of esophageal cancer (15). Generally speaking, it is difficult to carry out special randomized controlled trials to compare the differences in the adverse event spectrum of different ICIs, because the occurrence of these adverse events is difficult to predict, and the rates of grade 3–5 adverse events are very low (8). Therefore, meta-analysis is an effective research method for studies focusing on adverse events of ICIs. Network meta-analysis (NMA) may be applied to integrate all available evidence from phase II or phase III RCTs to get direct or indirect comparisons of different ICIs, especially when head-to-head RCTs among regimens are lacking (16). To the best of our knowledge, no NMA of ICI regimens that explored the spectrum of adverse events of immunotherapy is available yet in advanced esophageal or gastroesophageal junction cancer. Therefore, we conducted a systematic review to investigate the safety and adverse event profiles of ICIs for advanced esophageal or gastroesophageal junction cancer using NMA.

## Methods

The current study was conducted according to the Preferred Reporting Items for Systematic Reviews and Meta-analyses (PRISMA) (17), and the quality control and quality assurance (QC and QA) of the manuscript were instructed by the corresponding authors (JL and JZ).

### Search strategy and inclusion criteria for clinical trials

Relevant clinical trials published in various databases such as PubMed, Web of Science, and Cochrane Library were searched. Major conference proceedings including the Clinicaltrial.gov, American Society of Clinical Oncology (ASCO), and European Society for Medical Oncology (ESMO) databases were also searched for recent conference abstracts.

Relevant search terms relating to the present study were composed of various combinations of the medical subject headings (MeSH) and free-text terms. Search terms were combined by the Boolean operator “AND” or “OR” if necessary. A PubMed search was conducted using the following search terms: 1) search terms related to disease were “esophageal neoplasm,” “esophagus cancer,” “esophageal cancer,” “gastro-oesophageal junction cancer,” “gastro-esophageal junction cancer,” “cancer of the gastroesophageal junction,” “adenocarcinoma of the esophagus and gastroesophageal junction,” etc. (2) Search terms related to drugs or immunotherapy were “ipilimumab,” “pembrolizumab,” “nivolumab,” “atezolizumab,” “durvalumab,” “camrelizumab,” and other ICIs. The trade name of the drug includes “Yervoy,” “Keytruda,” “Opdivo,” “Tecentriq,” “Imfinzi,” etc. 3) Other search terms included “anti-CTLA-4 mab,” “anti-PDL1,” “anti-PD1,” “PD1 receptor,” “programmed cell death 1 protein,” “PD-1,” “PD-L1,” etc.

The selection criteria for clinical trials were organized according to the guidelines of the participants, interventions, comparisons, outcomes, and study design (PICOS) recommended by the Cochrane Collaboration. The inclusion criteria were as follows: i) the included patients were all pathologically diagnosed esophageal cancer or gastroesophageal junction cancer (GEJC) patients (P); ii) interventions of concern referred to immunotherapy with ICIs alone or in combination with chemotherapy (I); iii) controlled treatment regimens included chemotherapy alone (ChT) or best supportive treatment (BST), but there were no restrictions related to the chemotherapy regimens and chemotherapy cycles (C); iv) five safety outcomes included rates of treatment-related adverse events (trAEs), immune-related adverse events (irAEs), death, discontinuation of therapy, and grades 3–5 organ-special adverse events (O); and v) all randomized, open-label, controlled clinical trials with efficacy and safety data of ICIs were included. Although priority was given to phase III clinical trials, phase II clinical trials with a control group would be also included.

The exclusion criteria were as follows: i) phase I clinical trials and non-RCT studies, ii) participants with other tumors, iii) case reports and reviews, iv) incomplete data or non-original research, and v) repeated publications.

Articles were only included if they were published in English, but there was no restriction related to publication year. Two researchers (JZ and BH) were assigned to independently review all the data. If there were repeated articles in the selected clinical trials, only the latest published articles will be used for the final analysis. After the discussion according to the inclusion criteria and reaching a consensus, a decision was made to finally include or exclude the eligible articles. If a consensus cannot be reached, the corresponding author (JL) of this article is responsible for the final ruling.

### Data extraction and quality assessment

After reading the full text, two researchers (JZ and Tingting Li) extracted and cross-checked the data, including the following: 1) basic information: such as the title of the trial, author’s name, year of publication, source of literature, etc.; 2) methodological information of the trial: the sample size of the study included, the basic information of the study population, including the entry time and number of participants, disease stages, etc.; the randomization method of the trial, the evaluation method of important outcome indicators; median follow-up duration, death, and withdrawal, etc.; 3) detailed information on intervention measures: ICI medication, medication in the control group, etc.; and 4) detailed information for safety outcome indicators mentioned above. Disagreements were resolved by consensus.

Two independent researchers evaluated the included RCTs according to the bias risk assessment method recommended by the Cochrane Assistance Network. The evaluation methodological criteria and items were as follows: 1) generation of random allocation sequence, 2) the method of allocation concealment, 3) the method of blinding the patient, 4) the method of blinding the doctor or the therapist, 5) the method of blinding the data collection and analysis personnel, 6) the incomplete data reported, 7) selective reporting bias, and 8) other potential bias affecting authenticity.

We evaluate the risk of bias for each RCT according to the following criteria: “Yes” indicates a low risk of bias, “No” indicates a high risk of bias, and “Unclear” indicates that the literature does not provide sufficient information for bias assessment. The two researchers discussed according to the above standards and methods and, if necessary, reached a consensus according to the opinions of the third researcher.

### Statistical analysis

Adverse events including trAEs and irAEs were evaluated from two different perspectives: overview and detail. An overview analysis involved all kinds of AEs observed in ≥ grade 3–5 or all grade of the study population, and a detailed analysis involved some prespecified AEs of interest observed in ≥ grade 3–5 or all grades of the study population. The detailed information of related safety was extracted from the original literature and recorded as the number of events reported and no events for each specific treatment, respectively. If enough data were available to achieve network meta-analysis, a random-effects NMA was conducted in the frequency framework, using the command of “network” in Stata 16.0. Direct or indirect safety effects were combined into some summary statistics, that is, risk ratios (RRs) and 95% credibility intervals, to quantify the effect of adverse events in the network meta-analysis. Risk ratios less than 1 represented a beneficial effect favoring the ICI group. Two-sided *P <*0.05 indicated that the comparison was statistically significant. If the data were unavailable for the NMA, a conventional pairwise meta-analysis based on the random-effects model or the fixed-effects model was conducted depending on the size of heterogeneity. In this case, the outcome of interest may be grouped by whether ICIs were given. Heterogeneity was assessed using the chi-square test and *I* ^2^ statistics. *I* ^2^ ≥50% indicated obvious heterogeneity, and a random-effects model should be applied for pooled analysis. The classic half-integer continuity correction, that is adding 0.5 to each cell, was used in the data preprocessing stage if zero adverse events in any arm were reported.

The pooled rates of grade 3–5 or all-grade adverse events for treatments were meta-analyzed by the command of “metan” in STATA 16.0. Subgroup analyses for RRs between the ICI-treated group and the control group were performed based on the panoramic analysis, and prespecified, exploratory stratification factors for subgroup analyses involved the phase of the study (phase II versus phase III), treatment lines (first line, second line, and third line), ICI drug type (anti-PD-1, anti-CTLA-4, anti-PD-L1), treatment mode (ICIs alone versus ICIs combined with ChT), sample size (<500 versus ≥500), etc.

## Results

### Eligible studies and characteristics

In the literature retrieval stage, a total of 459 articles were obtained through preliminary screening. After reading the titles and abstracts, 422 articles including duplicate reports, irrelevant articles, non-randomized controlled trials, review articles, and phase I trials were excluded. The remaining 20 articles were excluded based on the selection criteria after reading the full text. Finally, a total of 11 trials reported in 17 articles met the inclusion criteria, of which 6 articles were updated or subgroup reports (18–30). Therefore, 11 articles were included in the meta-analysis, all of which were published in English (18, 20–25, 27–30). Updated reports or subgroup reports included three clinical trials, which were ATTRACTION−3 (19), ATTRACTION-4 (26), and ATTRACTION-2 (31–34).

**Figure 1:**
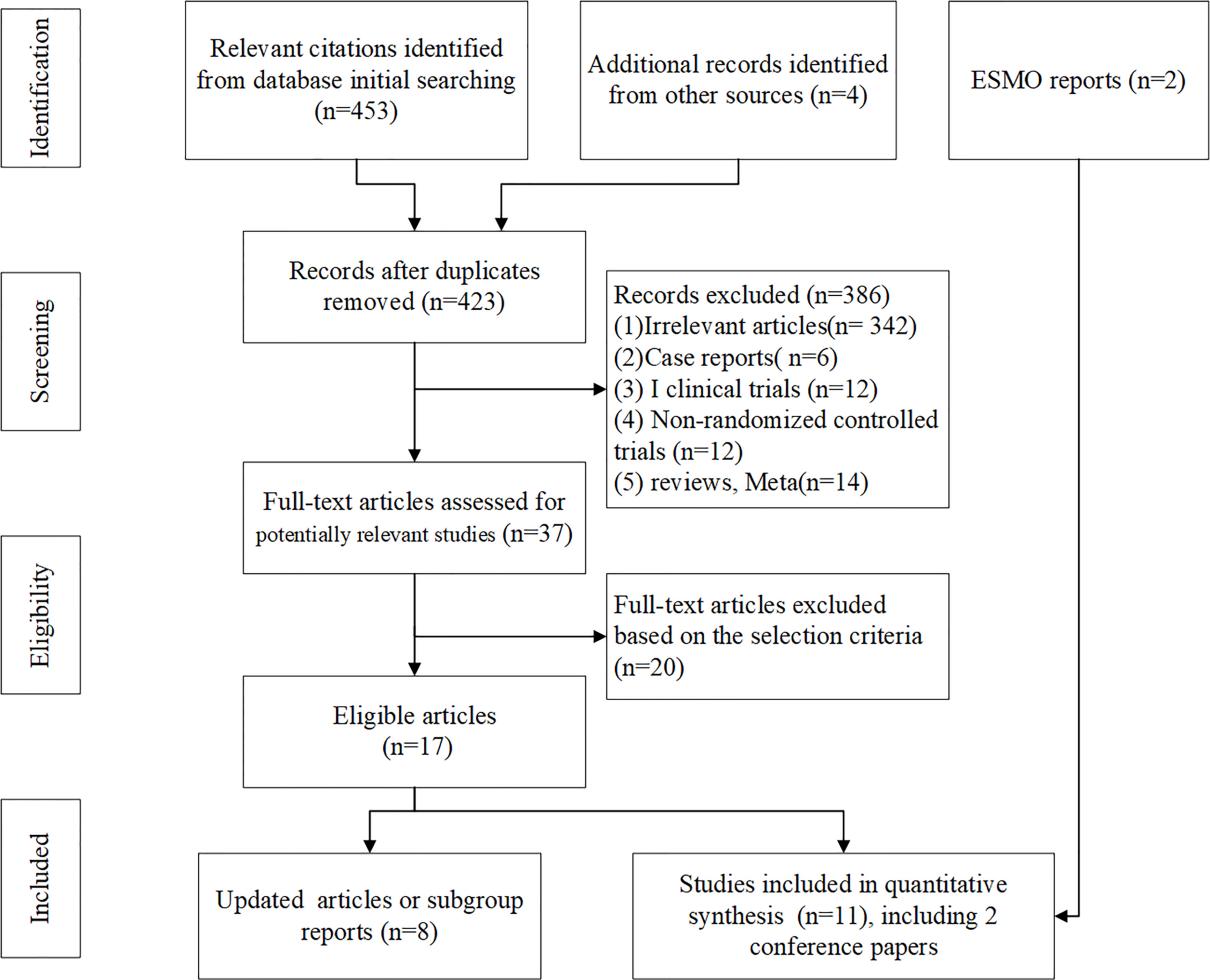
Flowchart of the study selection and design.

**Figure 1** shows the flowchart of the study selection and design procedure. The baseline characteristics of the 11 studies are summarized and shown in **Table 1**. All 11 studies included 7,089 patients, and the number of analysis population for AEs was 6,992. Most patients included came from an international multicenter. The cancer types included in the study were esophageal carcinoma (18, 20–23) and gastroesophageal junction cancer (24, 25, 27–30). Only one trial was a phase II study (28). First-line ICIs were applied in four clinical trials (22–25), second-line ICIs were applied in five clinical trials (18, 20, 21, 27, 28), and third-line ICIs were applied in two clinical trials (29, 30). Monotherapy with ICIs was found in six clinical trials (18, 20, 21, 27–30). In particular, anti-PD-L1 ICIs were used in only one trial (29), and anti-CTLA-4 ICIs were used in only two trials (23, 28).

**Table 1:** Characteristics of the included studies.

Study name	References	Trial phase	Treatment line	Cancer type	Treatment	ICI type	Treatment mode	No. of patients	Analysis population for AEs
ATTRACTION-3	32 (18)	III	Second-line	EPC	ChT			209	208
		III	Second-line	EPC	Nivo	PD-1	ICIs	210	209
ESCORT	20 (20)	III	Second-line	EPC	ChT			220	220
		III	Second-line	EPC	Camr	PD-1	ICIs	228	228
KEYNOTE-181	21 (21)	III	Second-line	EPC	ChT			314	296
		III	Second-line	EPC	Pemb	PD-1	ICIs	314	314
KEYNOTE-590	22 (22)	III	First-line	EPC	ChT			376	370
		III	First-line	EPC	Pemb + ChT	PD-1	ICIs + ChT	373	370
CheckMate-648	23 (23)	III	First-line	EPC	ChT			324	304
		III	First-line	EPC	Nivo + ChT	PD-1	ICIs + ChT	321	310
		III	First-line	EPC	Nivo + Ipil + ChT	CTLA-4	ICIs + ChT	325	322
CheckMate-649	Moehler et al., 2020s2202020202020 (24)	III	First-line	GEJC	ChT			792	792
		III	First-line	GEJC	Nivo + ChT	PD-1	ICIs + ChT	789	789
Study name	References	Trial phase	Treatment line	Cancer type	Treatment	ICI type	Treatment mode	No. of patients	Analysis population for AEs
ATTRACTION-4	25 (25)	III	First-line	GEJC	ChT			362	362
		III	First-line	GEJC	Nivo + ChT	PD-1	ICIs + ChT	362	362
KEYNOTE-061	27 (27)	III	Second-line	GEJC	ChT			296	276
		III	Second-line	GEJC	Pemb + ChT	PD-1	ICIs + ChT	296	294
NCT01585987	28 (28)	II	Third-line	GEJC	Placebo			57	57
		II	Third-line	GEJC	Ipil	CTLA-4	ICIs	57	57
JAVELIN Gastric 300	29 (29)	III	Third-line	GEJC	ChT			186	177
		III	Third-line	GEJC	Avel	PD-L1	ICIs	185	184
ATTRACTION-2	30 (30)	III	Third-line	GEJC	Placebo			163	161
		III	Third-line	GEJC	Nivo	PD-1	ICIs	330	330

EPC, esophageal cancer; GEJC, gastroesophageal junction cancer; ChT, chemotherapy; Nivo, nivolumab; Camr, camrelizumab; Pemb, pembrolizumab; Ipil, ipilimumab; Avel, avelumab; ICIs, immune checkpoint inhibitors; PD-1, programmed cell death-1; PD-L1, programmed cell death ligand 1; CTLA-4, cytotoxic T lymphocyte associate protein-4.

### Risk of bias

**Figure 2:**
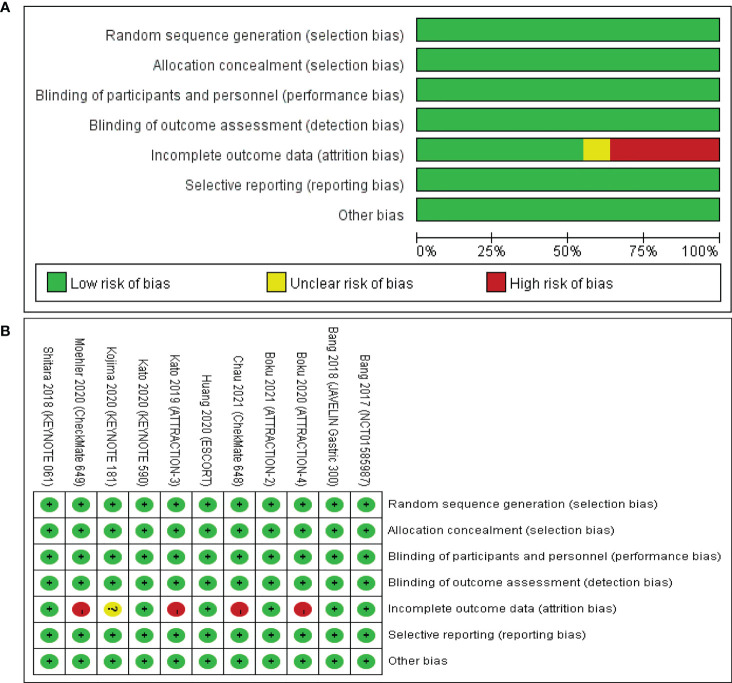
Risk of bias assessment for the included studies. Green for low risk of bias, yellow for unclear risk of bias, and red for high risk of bias. **(A)** The risk of bias graph shows an overall risk of bias for each item. **(B)** The risk of bias summary shows the detailed risk of bias of each item for each study.

The risk of bias assessment for the included studies involving the 11 articles is summarized and shown in **Figures 2A, B**. Four clinical trials were judged to be at high risk of bias mainly due to incomplete outcome data for major results of irAEs (18, 23–25). One clinical trial was judged to be at unclear risk of bias because of the lack of total results of grade 3–5 irAEs (21). The remaining studies had a low risk of bias and can be considered high quality.

### Network meta-analysis for trAEs

**Figure 3:**
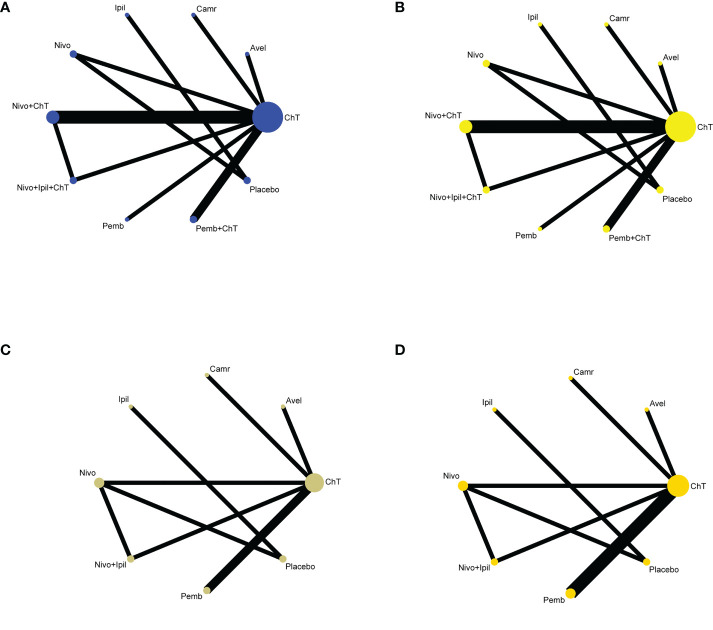
Network plots of comparisons with **(A)** grade 3–5 trAEs, **(B)** all-grade trAEs, **(C)** grade 3–5 irAEs, and **(D)** all-grade irAEs based on the network meta-analyses. ChT, chemotherapy; Avel, avelumab; Camr, camrelizumab; Ipil, ipilimumab; Nivo, nivolumab; Pemb, pembrolizumab.

Only one trial had not reported the results of grade 3–5 trAE (25). Therefore, data from the other studies can be successfully applied to implement NMA. A total of 3,005 patients (nine trials) were assigned to ChT therapy, 184 patients (one trial) to avelumab therapy, 228 patients (one trial) to camrelizumab therapy, 57 patients (one trial) to ipilimumab therapy, 539 patients (two trials) to nivolumab therapy, 1,461 patients (three trials) to nivolumab plus ChT therapy, 322 patients (one trial) to nivolumab + ipilimumab + ChT therapy, 314 patients (one trial) to pembrolizumab therapy, 664 patients (one trial) to pembrolizumab plus ChT therapy, and 218 patients (two trials) to placebo therapy. The network plot is shown in **Figures 3A, B**.

In the consistency model, for the rates of grade 3–5 trAEs, the results with significant benefits for different pairwise comparisons could be found in avelumab versus ChT, nivolumab versus ChT, pembrolizumab versus ChT, placebo versus ChT, placebo versus nivolumab + ChT, and placebo versus pembrolizumab + ChT. The results with significant increasing risk could be found in nivolumab + ChT versus avelumab, nivolumab + ipilimumab + ChT versus avelumab, pembrolizumab + ChT versus avelumab, nivolumab + ChT versus camrelizumab, nivolumab + ChT versus nivolumab, and nivolumab + ipilimumab + ChT versus nivolumab.

**Figure 4:**
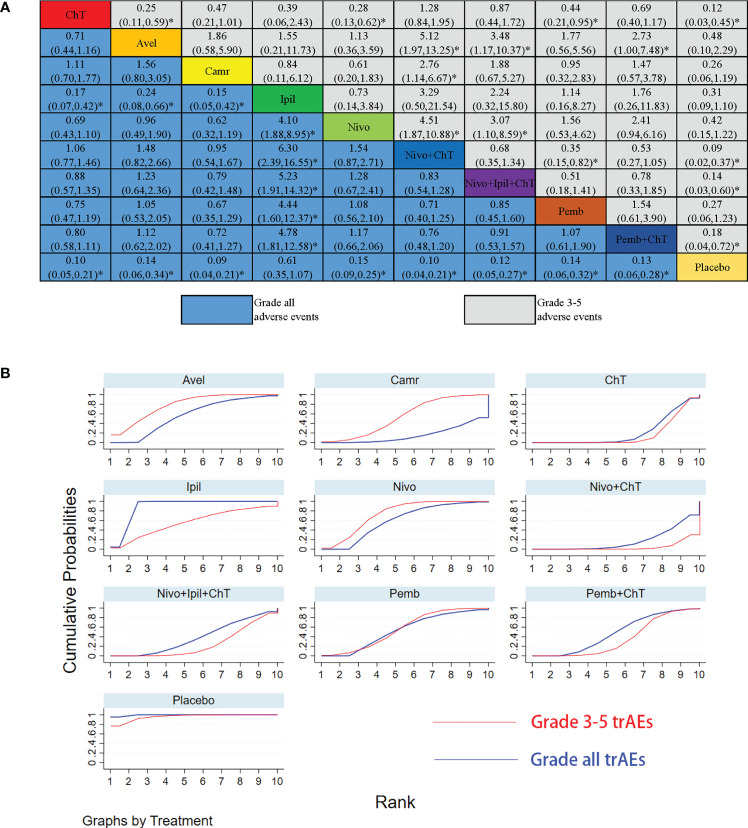
Results of the network meta-analysis for 10 treatment regimens in terms of treatment-related adverse events(trAEs) with grade 3–5 trAEs and all-grade trAEs. **(A)** League table for different treatment regimens. Relative effects (RRs) with 95% confidence intervals are shown for different treatment regimens compared with each other. The RR for a given comparison could be read in the intersection of two treatments. All *Z*-tests to compare two treatments were performed two-sided. *P < 0.05. ChT, chemotherapy; Avel, avelumab; Camr, camrelizumab; Ipil, ipilimumab; Nivo, nivolumab; Pemb, pembrolizumab. Placebo also involves the best supportive care. **(B)** The surface under the cumulative ranking curves (SUCRAs) for grade 3–5 trAEs and all-grade trAEs.

For the rates of all-grade trAEs, the results with significant benefits for different pairwise comparisons could be found in ipilimumab versus ChT, placebo versus ChT, ipilimumab versus avelumab, placebo versus avelumab, ipilimumab versus camrelizumab, placebo versus camrelizumab, placebo versus nivolumab, placebo versus nivolumab + ChT, placebo versus nivolumab + ipilimumab + ChT, placebo versus pembrolizumab, and placebo versus pembrolizumab + ChT. The results with significant increasing risk could be found in nivolumab versus ipilimumab, nivolumab + ChT versus ipilimumab, nivolumab + ipilimumab + ChT versus ipilimumab, pembrolizumab versus ipilimumab, and pembrolizumab + ChT versus ipilimumab. The details of all comparisons are indicated in **Figure 4A**. The ranking of benefits for different treatment regimens was assessed by the surface under the cumulative ranking curves (SUCRAs) and is shown in **Figure 4B**.

**Figure 5:**
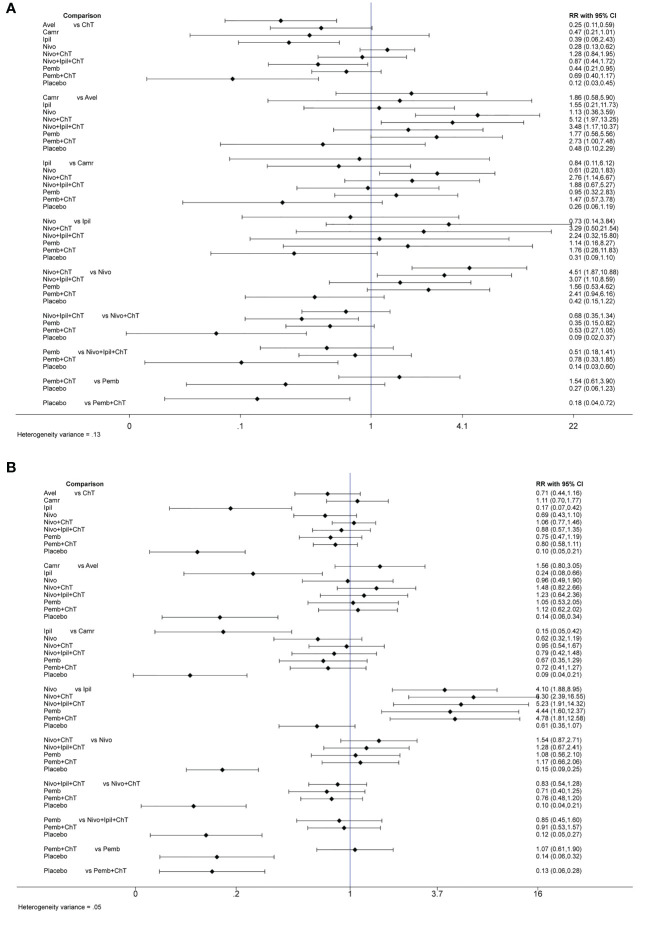
Forest plots for pairwise comparisons of all individual regimens with each other with **(A)** forest plots for grade 3–5 trAEs and **(B)** forest plots for all-grade trAEs.

As shown in **Figure 4B**, placebo was associated with the best safety ranking for grade 3–5 trAEs (SUCRA = 96.0%), followed by avelumab (78.6%), nivolumab (73.9%), ipilimumab (57.0%), and pembrolizumab (56.6%); placebo was associated with the best safety ranking for all-grade trAEs (99.5%), followed by ipilimumab (89.3%), nivolumab (60.1%), avelumab (56.8%), and pembrolizumab (53.5%). The relevant SUCRA values for the different treatments are detailed in **Supplementary Material Table S1**. Forest plots for pairwise comparisons of all individual regimens and their combinations are shown in **Figures 5A, B**.

For the rates of grade 1–2 trAEs, nivolumab + ChT was associated with the best safety ranking for grade 1–2 trAEs (88.2%), followed by pembrolizumab + ChT (85.1%), nivolumab + ipilimumab + ChT (82.4%), ChT (53.5%), and placebo (53.3%). The results of NMA are indicated in **Supplementary Figures S1**, **S2A, B**, and **S3** and **Table S1**.

### Subgroup analysis for trAEs

Stratification factors used for subgroup analyses included treatment lines (first line, second line, and third line), ICI drug type (anti-PD-1, anti-CTLA-4, anti-PD-L1), treatment mode (ICIs alone versus ICIs combined with ChT), and sample size (<500 versus ≥500). Based on the panoramic analysis of whether ICI treatment was applied, although the overall rates of grade 3–5 and all-grade trAEs were similar between the two groups, there were statistical differences in the rates of trAEs in some subgroups. For first-line treatment, ICIs were usually applied in combination with chemotherapy; consequently, the additional ICIs had significantly increased the rates of grade 3–5 trAEs (RR = 1.159, 95% CI = 1.012 to 1.327). However, for second-line treatment, ICIs had significantly decreased the rates of grade 3–5 trAEs (RR = 0.395, 95% CI = 0.317 to 0.491). In the case of ICIs alone, compared with chemotherapy, ICIs significantly reduced the rates of grade 3–5 trAEs (RR = 0.584, 95% CI = 0.350 to 0.974). The detailed results for subgroup analyses are listed in **Table 2**. Forest plots for subgroup analyses are indicated in **Supplementary Figures S4A–E** and **S5A–S5E**.

**Table 2:** Subgroup analysis of risk ratios for treatment-related adverse events (trAEs) comparing ICI therapy with chemotherapy.

Subgroup	Grade 3–5 trAEs			All-grade trAEs		
analysis^a^	*I* ^2^ (*P*)	RR (95% CI)	*Z* and *P*	*I* ^2^ (*P*)	RR (95% CI)	*Z* and *P*
Overall	95.7% (0.000)	0.764 (0.574, 1.016)	*Z* = 1.85, *P* = 0.065	96.7% (0.000)	0.916 (0.831, 1.010)	*Z* = 1.77, *P* = 0.077
Subgroup						
Treatment lines						
Second-line	52.1% (0.100)	0.395 (0.317, 0.491)	*Z* = 8.30, *P* = 0.000	97.6% (0.000)	0.762 (0.570, 1.019)	*Z* = 1.83, *P* = 0.067
First-line	80.1% (0.000)	1.159 (1.012, 1.327)	*Z* = 2.13, *P* = 0.033	89.5% (0.000)	1.006 (0.952, 1.062)	*Z* = 0.20, *P* = 0.841
Third-line	94.2% (0.000)	1.198 (0.199, 7.200)	*Z* = 0.20, *P* = 0.843	95.2% (0.000)	1.190 (0.600, 2.358)	*Z* = 0.50, *P* = 0.619
ICI drug type						
PD-1	96.2% (0.000)	0.773 (0.566, 1.057)	*Z* = 1.61, *P* = 0.106	97% (0.000)	0.919 (0.827, 1.020)	*Z* = 1.59, *P* = 0.111
CTLA-4	82.1% (0.000)	1.531 (0.434, 5.404)	*Z* = 0.66, *P* = 0.508	93.1% (0.000)	1.177 (0.614, 2.258)	*Z* = 0.49, *P* = 0.624
PD-L1	–	0.251 (0.156, 0.404)	*Z* = 5.69, *P* = 0.000	–	0.661 (0.557, 0.785)	*Z* = 4.73, *P* = 0.000
Treatment mode						
ICIs alone	88.7% (0.000)	0.584 (0.350, 0.974)	*Z* = 2.06, *P* = 0.039	95.40% (0.000)	0.952 (0.755, 1.200)	*Z* = 0.42, *P* = 0.678
ICIs + ChT	91.6% (0.000)	1.007 (0.818, 1.239)	*Z* = 0.06, *P* = 0.950	96.80% (0.000)	0.926 (0.836, 1.025)	*Z* = 1.49, *P* = 0.136
Sample size						
<500	90.9% (0.000)	0.663 (0.327, 1.344)	*Z* = 1.14, *P* = 0.254	95.50% (0.000)	1.012 (0.760, 1.348)	*Z* = 0.08, *P* = 0.933
≥500	94.4% (0.000)	0.892 (0.697, 1.142)	*Z* = 0.90, *P* = 0.366	97.60% (0.000)	0.891 (0.795, 0.998)	*Z* = 1.99, *P* = 0.047

a Subgroup analyses were conducted based on the pairwise comparisons of all individual trials.

### Meta-analysis of serious trAEs, events leading to discontinuation, and treatment-related death

Only five clinical trials had provided detailed data comparing the rates of serious trAEs between ICIs and chemotherapy (18, 20, 23, 24, 28). The meta-analysis shows that the rates of serious trAEs in the ICI group and the chemotherapy group were 22.66% (434/1,915) and 11.46% (216/1,885), respectively. However, no significant difference between the two groups was found (RR = 1.786, 95% CI = 0.978 to 3.262, *Z* = 1.89, *P* = 0.059). Six clinical trials had provided detailed data comparing the rates of events leading to discontinuation (18, 20–24). The

meta-analysis shows that the rates of events leading to discontinuation in the ICI group and the chemotherapy group were 22.42% (570/2542) and 11.59% (289/2,494), respectively, without statistical significance (RR = 1.447, 95% CI = 0.908 to 2.307, *Z* = 1.55, *P* = 0.120). Five clinical trials had provided detailed data comparing the rates of treatment-related death (18, 20–23). The meta-analysis shows that the rates of treatment-related death in the ICI group and the chemotherapy group were 1.88% (33/1,753) and 1.41% (24/1,702), respectively, without statistical significance (RR = 1.335, 95% CI = 0.793 to 2.249, *Z* = 1.09, *P* = 0.277). The corresponding forest plots are shown in **Supplementary Figures S6A–C**.

### Meta-analysis based on specific treatment-related adverse events

**Figure 6:**
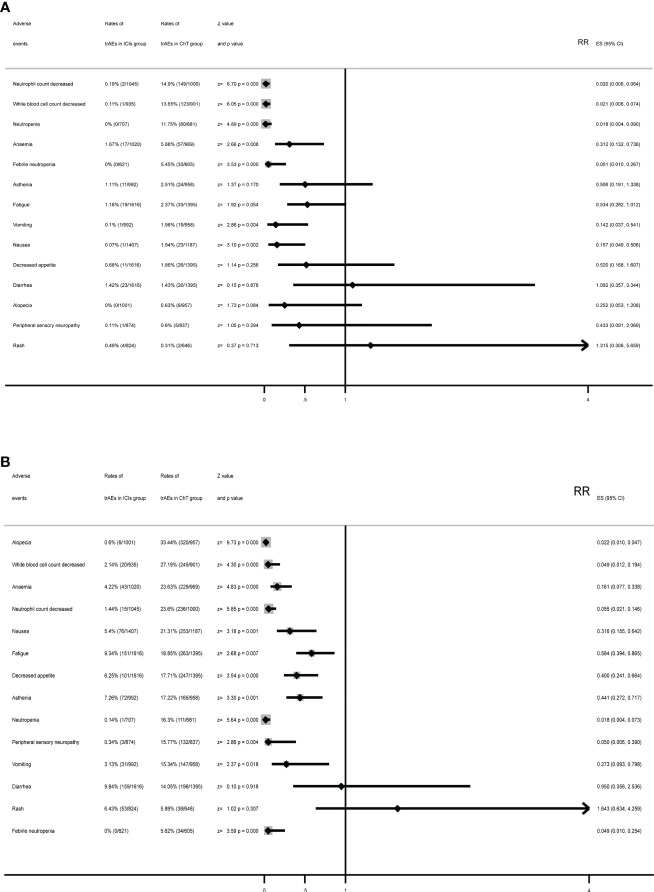
Summary forest plots for specific treatment-related adverse events with **(A)** forest plots for grade 3–5 trAEs and **(B)** forest plots for all-grade trAEs.

The meta-analysis for some specific treatment-related adverse events of interest is listed in **Figures 6A, B**. Compared with chemotherapy, in the ICI group, the rates for grade 3–5 trAEs of the following had been significantly reduced: decreased neutrophil count, decreased white blood cell count, neutropenia, anemia, febrile neutropenia, vomiting, and nausea. Moreover, the rates of the following in the ICI group were similar: asthenia, fatigue, decreased appetite, diarrhea, alopecia, peripheral sensory neuropathy, and rash. The highest rates of adverse events in the chemotherapy group were decreased neutrophil count (14.9%), followed by decreased white blood cell count (13.65%), neutropenia (11.75%), anemia (5.88%), and febrile neutropenia (5.45%). However, the most common adverse events in the ICI group were anemia (1.67%), followed by diarrhea (1.42%), fatigue (1.18%), and asthenia (1.11%).

Except for diarrhea and rash, ICIs had significantly reduced the rates of specific treatment-related adverse events. The most common all-grade trAEs were alopecia (33.44%), followed by decreased white blood cell count (27.19%), anemia (23.63%), decreased neutrophil count (23.6%), and nausea (21.31%) in the chemotherapy group and diarrhea (9.84%) in the ICI group, followed by fatigue (9.34%), asthenia (7.26%), rash (6.43%), and decreased appetite (6.25%).

### Network meta-analysis for irAEs

Only seven and eight trials had provided data on the rates of grade 3–5 irAEs (20, 22, 23, 27–30) and all-grade irAEs (20–23, 27–30), respectively. The network plot is shown in **Figures 3C, D**.

**Figure 7:**
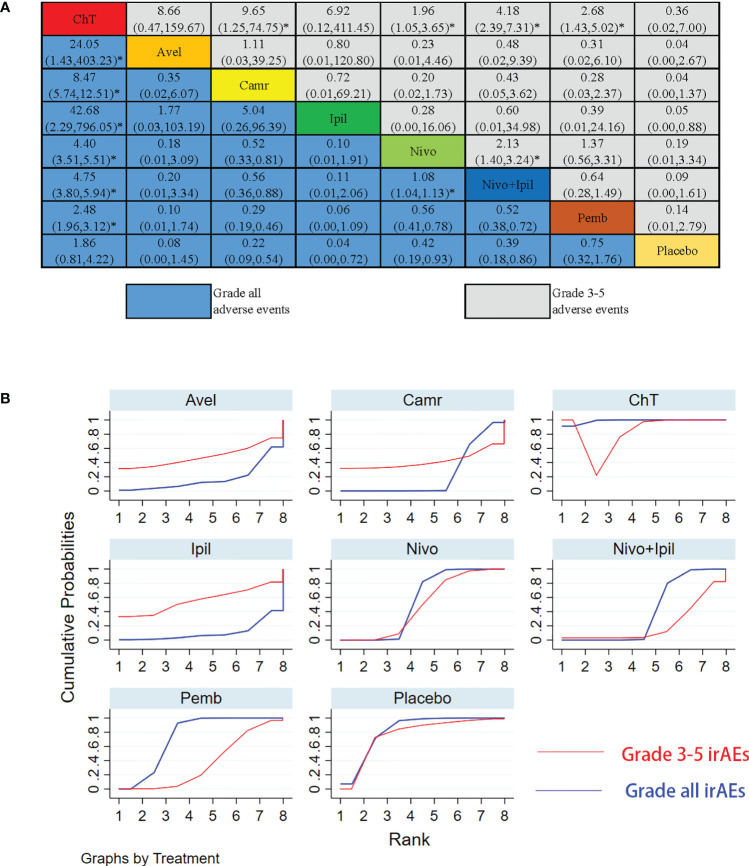
Results of the network meta-analysis for 10 treatment regimens in terms of immune-related adverse events (irAEs) with grade 3–5 irAEs and all-grade irAEs. **(A)** League table for different treatment regimens. **(B)** The surface under the cumulative ranking curves (SUCRAs) for grade 3–5 irAEs and all-grade irAEs. *: P<0.05.

The NMA results of the consistency model for the rates of grade 3–5 irAEs and all-grade irAEs are indicated in **Figures 7A, B**. The general safety of grade 3–5 irAEs assessed by SUCRA for different ICI drugs or ChT ranked from high to low is as follows: ChT (85.1%), placebo (76.5%), ipilimumab (56.0%), nivolumab (48.5%), avelumab (48.4%), camrelizumab (41.8%), pembrolizumab (36.4%), and nivolumab + ipilimumab (21.6%). In terms of all-grade irAEs, the general safety of different ICI drugs or ChT ranked from high to low is as follows: ChT (98.7%), placebo (82.0%), pembrolizumab (73.6%), nivolumab (54.6%), nivolumab + ipilimumab (39.9%), camrelizumab (23.3%), avelumab (17.6%), and ipilimumab (10.3%). The SUCRA values are detailed in **Supplementary Table S2**.

**Figure 8:**
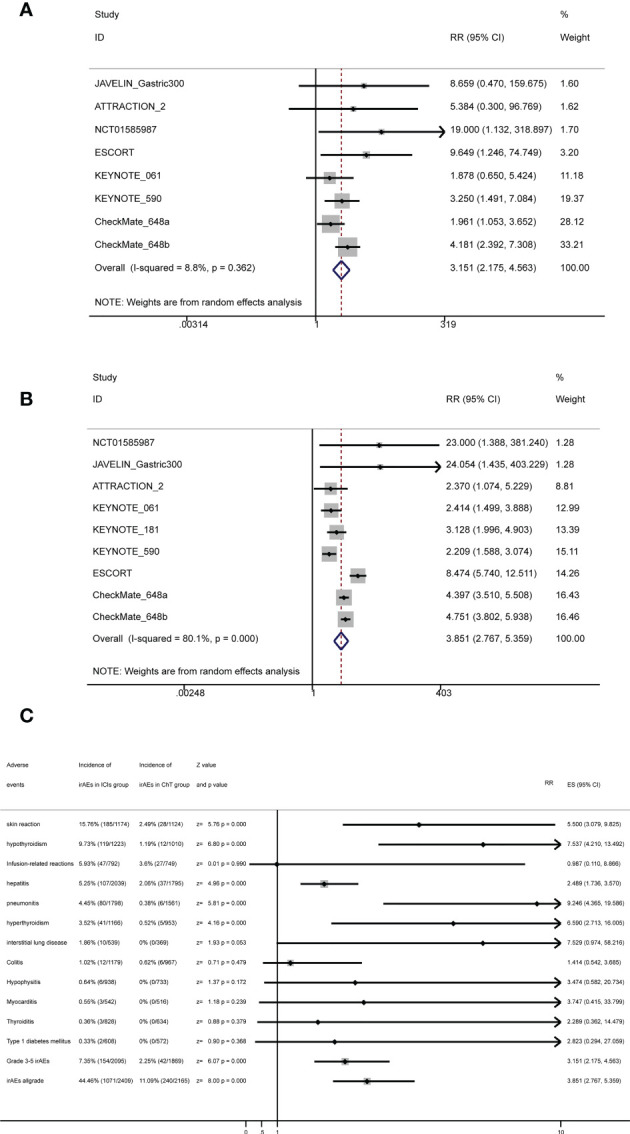
Forest plots for irAEs with **(A)** forest plots for grade 3–5 irAEs, **(B)** forest plots for all-grade irAEs, and **(C)** summary forest plots for irAEs.

Conventional pairwise meta-analysis was used to integrate all available data of irAEs. Seven clinical trials had provided detailed data comparing the rates of grade 3–5 irAEs between ICIs and chemotherapy (20, 22, 23, 27–30). The meta-analysis shows that the rates of grade 3–5 irAEs in the ICI group and the chemotherapy group were 7.35% (154/2,095, 95% CI: [6.23%, 8.47%]) and 2.25% (42/1,869, 95% CI: [1.58%, 2.92%]), respectively, with statistical significance (RR = 3.151, 95% CI = 2.175 to 4.563, *Z* = 6.07, *P* = 0.000). Eight clinical trials had provided detailed data comparing the rates of all-grade irAEs between ICIs and chemotherapy (20–23, 27–30). The meta-analysis shows that the rates of all-grade irAEs in the ICI group and the chemotherapy group were 44.46% (1,071/2,409) and 11.09% (240/2,165), respectively, with statistical significance (RR = 3.851, 95% CI = 2.767 to 5.359, *Z* = 8.00, *P* = 0.000). Therefore, immunotherapy not only increased immune-related adverse events of grades 3–5 but also increased immune-related adverse events of all grades. The corresponding forest plots are shown in **Figures 8A, B**.

### Subgroup analysis for irAEs

The stratification factors of irAEs were the same as those of trAEs. The detailed results for the subgroup analyses are listed in **Table 3**. Forest plots for subgroup analyses are indicated in **Supplementary Figures S7A–E** and **S8A–E**. In terms of grade 3–5 irAEs, except for the second-line treatment subgroup and the PD-L1 subgroup, it can be observed that ICIs had significantly increased the adverse events in almost all of the other subgroups. Moreover, it can be observed that ICIs had significantly increased all-grade irAEs in all subgroups.

**Table 3:** Subgroup analysis of risk ratios for immune-related adverse events (irAEs) comparing ICI therapy with chemotherapy.

Subgroup analysis^a^	Grade 3–5 irAEs			All-grade irAEs		
	*I* ^2^ (*P*)	RR (95% CI)	*Z* and *P*	*I* ^2^ (*P*)	RR (95% CI)	*Z* and *P*
Overall	95.7% (0.000)	3.151 (2.175, 4.563)	*Z* = 6.07, *P* = 0.000	80.1% (0.000)	3.851 (2.767, 5.359)	*Z* = 8.00, *P* = 0.000
Subgroup						
Treatment lines						
Second-line	51.6% (0.151)	3.387 (0.690, 16.635)	*Z* = 1.50, *P* = 0.133	89.8% (0.000)	4.036 (1.833, 8.888)	*Z* = 3.46, *P* = 0.001
First-line	37.6% (0.201)	3.011 (1.880, 4.823)	*Z* = 4.59, *P* = 0.000	87.0% (0.000)	3.653 (2.430, 5.493)	*Z* = 6.23, *P* = 0.000
Third-line	0.0% (0.823)	9.716 (1.849, 51.060)	*Z* = 2.69, *P* = 0.007	60.8% (0.078)	7.513 (1.096, 51.516)	*Z* = 2.05, *P* = 0.040
ICI drugs						
PD-1	0.0% (0.504)	2.484 (1.620, 3.807)	*Z* = 4.17, *P* = 0.000	85.3% (0.000)	3.464 (2.280, 5.262)	*Z* = 5.82, *P* = 0.000
CTLA-4	9.4% (0.293)	4.729 (2.071, 10.798)	*Z* = 3.69, *P* = 0.000	19.0% (0.267)	5.562 (2.176, 14.215)	*Z* = 3.58, *P* = 0.000
PD-L1	–	8.659 (0.470, 159.675)	*Z* = 1.45, *P* = 0.147	–	24.054 (1.435, 403.229)	*Z* = 2.21, *P* = 0.027
Treatment mode						
ICIs alone	0.0% (0.943)	9.690 (2.670, 35.166)	*Z* = 3.45, *P* = 0.001	76.9% (0.002)	5.099 (2.396, 10.850)	*Z* = 4.23, *P* = 0.000
ICIs + ChT	23.4% (0.270)	2.839 (1.892, 4.261)	*Z* = 5.04, *P* = 0.000	84.5% (0.000)	3.357 (2.320, 4.858)	*Z* = 6.42, *P* = 0.000
Sample size						
<500	0.0% (0.943)	9.690 (2.670, 35.166)	*Z* = 3.45, *P* = 0.001	68.8% (0.022)	6.573 (2.383, 18.128)	*Z* = 3.64, *P* = 0.000
≥500	23.4% (0.270)	2.839 (1.892, 4.261)	*Z* = 5.04, *P* = 0.000	80.0% (0.000)	3.329 (2.432, 4.559)	*Z* = 7.50, *P* = 0.000

a Subgroup analyses were conducted based on the pairwise comparisons of all individual trials.

### Meta-analysis based on specific immune-related adverse events

Some specific immune-related adverse events of interest are listed in **Figure 8C** and **Supplementary Table S3**. The most common irAEs in the ICI group were skin reaction (15.76%, 95% CI: [13.67%, 17.84%]), followed by hypothyroidism (9.73%, 95% CI: [8.07%, 11.39%]), infusion-related reactions (5.93%, 95% CI: [4.29%, 7.58%]), hepatitis (5.25%, 95% CI: [4.28%, 6.22%]), and pneumonitis (4.45%, 95% CI: [3.5%, 5.4%]).

## Discussion

Immunotherapy based on ICIs has currently become one of the most promising treatment regimens for cancer, which plays an encouraging role in the treatment of advanced cancer (35). Under normal physiological conditions, immune checkpoints help in maintaining self-tolerance and protecting host tissues from damage by the immune system when the immune system responds to specific physiological and pathological conditions (36). Tumor cells take full advantage of this feature to escape the attack of immune cells (37). Currently, the CTLA-4/B7-1/2 and PD-1/PD-L1 pathways has become the most popular in the field of cancer research on immunotherapy, both of which are the key pathways for immune T-cell activation (38). Most ICIs change the activity of immune checkpoints by targeting the inhibitory receptors (IRs) CTLA-4, PD-1, or PD-L1 and reactivate the immune response of T cells to tumor cells, thereby achieving antitumor effects (39). As immunotherapeutics have made substantial clinical progress in a variety of solid tumors, many PD-1/PD-L1 and CTLA-4 inhibitors have been approved by the FDA and can be used alone or combined with surgery, chemotherapy, radiotherapy, targeted therapy, and other therapeutic methods for many tumors (40). Due to the lack of effective treatment strategies, patients with advanced esophageal cancer generally have poor long-term survival and quality of life (41). Chemotherapy has been the main treatment strategy for patients with advanced esophageal cancer, but there is a serious lack of effective systemic chemotherapy regimens (42). The ATTRACTION-1 (43), KEYNOTE-028 (44), and KEYNOTE-180 (45) studies confirmed the efficacy and safety of immunotherapy in the second-line and third-line treatment of advanced esophageal cancer. The KEYNOTE-590 and CheckMate-648 studies further established the fundamental status of ICIs in the first-line treatment of advanced or resectable esophageal squamous cell carcinoma (22, 23). Although immunotherapeutics have special antitumor effects compared with cytotoxic chemotherapy and molecular targeted therapy, treatment- and immune-related adverse events should deserve attention and research (46). Some studies had shown that the toxicity of ICI drugs is generally lower than that of standard chemotherapy, but serious irAEs of ICI drugs will still be reported in clinical trials from time to time (47). In this review, we focused on the rates of trAEs and irAEs for ICIs in different treatment lines for advanced esophageal cancer. Meta-analysis was conducted based on 11 published RCTs to evaluate the safety of ICIs. In this review, we systematically describe the rates and influencing factors of various adverse events caused by ICIs in patients with advanced esophageal cancer or gastroesophageal junction cancer. Now, we discuss the problems discovered during the study process as follows.

In this review, we have included a total of 11 studies, including 7,089 patients, of which 6,992 cases can be used for adverse event analysis. As far as we know, the current meta-analysis may be the study with the largest sample size to explore the possible adverse events of immunotherapy in esophageal cancer and gastroesophageal junction cancer. Based on the results of our NMA analysis of different lines of immunotherapy for esophageal/gastroesophageal junction cancer, we can draw five main conclusions that may affect clinical practice.

First of all, from the point of view of different treatment modalities, different combinations of treatment modalities had obviously distinct safety outcomes in trAEs and irAEs. Similar to the results of practice in lung cancer, ICIs were generally less toxic in monotherapy than in chemotherapy, and the combination of ICIs and chemotherapy would increase the rates of grade 3–5 trAEs and grade 3–5 irAEs (48, 49). Although the overall survival and progression-free survival of combination therapy were significantly longer than those of chemotherapy alone, the treatment- and immuno-related toxicities had also been increased, which should not be underestimated (50). From the results of our network meta-analysis, compared with chemotherapy alone or ICIs alone, almost all combination treatments of ICIs and chemotherapy had increased the rates of treatment-related adverse events. Nivolumab + ChT, ChT, nivolumab + ipilimumab + ChT, and pembrolizumab + ChT had the smallest area under the SUCRA curve (see **Supplementary Table S1**), which means that these treatment modalities have the highest probability of grade 3–5 trAEs. From the perspective of monotherapy, the general safety of grade 3–5 trAEs for different ICI drugs ranked from high to low is as follows: avelumab, nivolumab, ipilimumab, pembrolizumab, and camrelizumab. However, camrelizumab had the highest rates of all-grade trAEs. Further analysis showed that increased all-grade trAEs are mainly caused by the increased occurrence of reactive capillary endothelial proliferation (RCEP), which was a skin reaction that rarely occurred in other ICIs but commonly manifested in camrelizumab. RCEP mostly appeared within 2 to 4 weeks after medication, most of which were grade 1 to 2 with rare grade 3–4 events occurring. The data showed that the rates of RECP were about 66.8%–70% in solid tumors (51, 52) and 80% in the ESCORT trial (20). In our meta-analysis, as only the ESCORT trial reported the data of RECP, the pooled analysis was not carried out. Our meta-results showed that the risk of all-grade trAEs for different treatment modalities ranked from high to low is as follows: camrelizumab, nivolumab + ChT, ChT, nivolumab + ipilimumab + ChT, and pembrolizumab + ChT. From the perspective of monotherapy, the general safety of all-grade trAEs for different ICI drugs ranked from high to low is as follows: ipilimumab, nivolumab, avelumab, and pembrolizumab (see **Supplementary Table S1**). Similar to the previous observations reported in related meta-analysis investigating the safety of ICIs, we confirmed that anti-programmed cell death ligand 1 ICI drugs (PD-L1) were safer than ChT in the subgroup analysis (49, 53, 54). A meta-analysis reported that 46% (95% CI 40–53) of patients who received the combination of immunotherapy and chemotherapy encountered grade ≥3 AEs, which was significantly higher than immunotherapy alone or chemotherapy alone (55).

Secondly, the application of ICI drugs in esophageal cancer involved first-line, second-line, third-line, or later-line treatment (56). In most cases, second-line treatment or later-line treatment would be dominated by single-agent therapy, including single-agent chemotherapy or single-agent immunotherapy (56). Monotherapy tended to be better tolerated, especially for patients with advanced tumors with poor ECOG score. In the first-line treatment, ICIs are usually used in combination with chemotherapy in the hope that the efficacy can be further improved (22–25). In our subgroup analysis, we found that ICIs applied in second-line treatment significantly reduced the rates of grade 3–4 trAEs, while ICIs applied in first-line treatment had the opposite performance, which further indicated that adding ICIs to chemotherapy will increase the treatment-related adverse events. Although the influence of treatment line on the rates of adverse events was largely due to different treatment combinations, our data showed that ICIs should be avoided as much as possible in combination with chemotherapy in the second- or third-line treatment for esophageal cancer to reduce the risk of adverse events (57).

Third, previous studies had shown that different types of ICIs have different toxicity profiles because of their different mechanisms of action (58). Anti-CTLA-4 drugs work by enhancing T-cell priming, while PD-1/PD-L1 inhibitors are thought to work by reactivating the pre-existing CD8 T-cell response (59). CTLA-4 inhibitors were generally considered to be more toxic, while PD-L1 inhibitors were considered to be more tolerable (60). A previous meta-analysis showed that 34% (95% CI 27–42) of patients treated with CTLA-4 inhibitors encountered grade ≥3 AEs, but only 14% (95% CI 12–16) of patients treated with PD-1/PD-L1 inhibitors suffered grade ≥3 AEs (55). In our meta-analysis, only two trials published the safety data on CTLA-4 inhibitors for esophageal cancer (23, 28). In terms of grade 3–4 trAEs, the risk of AEs caused by ipilimumab was lower than that of pembrolizumab and camrelizumab but higher than that of avelumab and nivolumab. This finding is different from previous reports and should be noted. On the other hand, from the perspective of immune-related adverse events, CTLA-4 inhibitors in our NMA had the highest risk of grade 1–5 irAEs, while the risk of grade 3–5 adverse events was relatively low. Compared with monotherapy, combined immunotherapy of nivolumab and ipilimumab had the highest risk of grade 3–5 irAEs. This finding was similar to the previously reported results (55), and the same findings have been found in clithe nical practice of lung cancer (35). Whether using CTLA-4 or PD-1/PD-L1 inhibitors, the application of ICIs significantly increased both the rates of grade 3–5 irAEs and grade 1–5 irAEs in our subgroup analyses. Therefore, a careful balance between toxicity and efficacy should be evaluated when ICIs need to be applied (55).

Fourth, the spectrum of trAEs caused by ICIs was also significantly different from that caused by ChT. Our meta-analysis based on specific treatment-related adverse events showed that ICIs were safer and had a significantly different spectrum of grade 3–5 trAEs and all-grade trAEs from chemotherapy. Hematological toxicity was the main adverse event for chemotherapy, while systemic symptoms such as fatigue, asthenia, and decreased appetite were the main adverse events for ICIs (50). For patients with poor bone marrow function, immunotherapy may be a better treatment option (61). For specific immune-related adverse events, our meta-analysis results showed that the most common irAEs in the ICI group were skin reaction (15.76%, 95% CI: [13.67%, 17.84%]), followed by hypothyroidism (9.73%, 95% CI: [8.07%, 11.39%]), infusion-related reactions (5.93%, 95% CI: [4.29%, 7.58%]), hepatitis (5.25%, 95% CI: [4.28%, 6.22%]), and pneumonitis (4.45%, 95% CI: [3.5%, 5.4%]). Due to the limited data obtained, we cannot further analyze the detailed rates of 3–5 grade irAEs and cannot further analyze the difference in the rates of specific trAEs and irAEs between CTLA-4 inhibitors and PD-1/PD-L1 inhibitors. However, the rates of all-grade-specific irAEs were close to the results of the previous meta-analysis reported. More specifically, colitis and hypophysitis seem to be more common with CTLA-4 inhibitors, whereas pneumonitis, hypothyroidism, and arthralgia appear to be more commonly associated with PD-1/PD-L1 inhibitors (55, 62, 63).

Finally, there was no consensus on whether the rates of irAEs were related to the primary site of the tumor. One review found that the rates of several specific AEs of interest varied among different cancer types (64). However, another review found that the overall rates of all-grade and grade 3–5 irAEs did not differ among different tumor sites (62). In our systematic review, both patients with esophageal cancer and gastroesophageal junction cancer were selected as the research subjects. Two reasons for this were the limited number of randomized controlled trials of immunotherapy for esophageal cancer and that some patients with esophageal adenocarcinoma had to be enrolled in some trials on GEJC. We did not further investigate whether specific irAEs differed between EPC and GEJC, which may be a potential focus for future analyses. In our view, the occurrence and severity of adverse events would be influenced by many factors, including the patients’ characteristics (disease stage, physical condition, age, gender, basic diseases, etc.). However, the rates were low in some special adverse events, especially for irAEs (47, 65). It is difficult to analyze the impact of these factors on the rates of AEs through the available data extracted from the literature, so we had to ignore the potential impact.

It should be pointed out from the results of our meta-analysis that, although ICIs increased the adverse events, the rates were actually low and acceptable. Although immunotherapy had increased the rates of irAEs, to a certain extent, the occurrence of immune-related events may be positively correlated with the therapy’s efficacy and the patient’s prognosis (66, 67). When focusing on the anti-tumor effects of ICIs, we should also pay attention to the occurrence of irAEs when ICIs are applied (68). However, we should not stop eating for fear of choking; after all, the current evidence showed that the benefits of ICIs outweigh the potential risks. For clinicians, the task we have to do is to achieve the best balance between the antitumor effects and the related adverse events of ICIs based on the best evidence-based medical practice.

There are some limitations in our review that need to be mentioned. First, the network meta-analysis assumes that the estimates of the study effects between the various trials have commonality, transferability, and exchangeability, which means that the similarities of population characteristics, interventions, chemotherapy regimen, and other features among different trials are required. However, as the conditions of the trials may affect the study results, this assumption is very unrealistic. In our meta-analysis, heterogeneity was detected in the results of grade 3–5 trAEs and all-grade trAEs. Subgroup meta-analyses revealed that trials with treatment line = second line, treatment line = first line, treatment mode, and a sample size ≥500 patients were potential sources of heterogeneity. Second, some specific irAEs and trAEs may be selectively reported in most trials because the rates of these adverse events were lower than a preset threshold, such as 1% or 5%. In this case, we cannot obtain the pooled estimates of rates for these rare adverse events, so it is inevitable to underestimate the overall mean rates of some adverse events. Third, in order to catch the latest data from newly published trials, some recent conference abstracts were enrolled in our meta-analysis, from which some summary data were extracted. However, this may lead to another selection bias because the comprehensive toxicity data might not be reported in these abstracts. Furthermore, some previous meta-analyses on this topic had shown the influence of different drug doses on the occurrence of adverse events (54). In our study, the related data on the influence of doses were not available. Therefore, we had to ignore this point. Finally, sometimes, serious adverse effects are either rare or not encountered. In this case, the confidence interval of the calculated effect estimate is too wide, which will affect the accuracy of the pooled effect size. This was extremely common in the evaluations of adverse events. In our meta-analysis, the rates of irAEs in arms without ICIs were very low, so a large number of wide-ranging estimates of RR appeared. Therefore, one should be cautious when interpreting the results of the meta-analysis and drawing conclusions.

## Conclusion

Monotherapy with immune checkpoint inhibitors displayed better safety profiles in terms of trAEs than chemotherapy alone; however, combinational treatment regimens involving ICIs increased the risk of trAEs. Different ICIs had different toxicity manifestations and should not be considered as an entity. Compared with chemotherapy, ICIs were more prone to irAEs, but the overall rates remained low and acceptable. For clinicians, it is important to recognize and monitor the adverse events caused by ICIs for patients with esophageal cancer or gastroesophageal junction cancer.

## Data availability statement

The original contributions presented in the study are included in the article/**Supplementary Material**. Further inquiries can be directed to the corresponding authors.

## Author contributions

JZ and LX collected the data. JZ and MW performed data cleaning and analysis. JZ and BH performed the systematic review. JZ and BH evaluated the data. JL drafted and reviewed the manuscript for scientific soundness. All authors contributed to the article and approved the submitted version.
