# Supplementary material for: Treatment- and immune-related adverse events of immune checkpoint inhibitors in esophageal or gastroesophageal junction cancer: A network meta-analysis of randomized controlled trials
Source: Front Oncol. 2022 Dec 8;12:821626. doi: 10.3389/fonc.2022.821626 (PMC9780048; doi:10.3389/fonc.2022.821626)
Supplement: Supplementary file 1 [file DataSheet_1.zip › Supplementary figures and tables/Supplementary.docx]

TABLE S1 | The relevant SUCRA values for different treatment for trAEs

| Treatment | Grade 1-2 | | Grade 3-5 | | Grade all | |
| --- | --- | --- | --- | --- | --- | --- |
|  | SUCRA | PrBest () | SUCRA | PrBest | SUCRA | PrBest |
| ChT | 53.5 | 0.0 | 16.7 | 0.0 | 21.6 | 0.0 |
| Avel | 40.7 | 1.2 | 78.6 | 15.6 | 56.8 | 0.0 |
| Camr | 12.1 | 0.0 | 54.7 | 1.1 | 15.3 | 0.0 |
| Ipil | 23.0 | 2.4 | 57.0 | 3.2 | 89.3 | 4.6 |
| Nivo | 10.2 | 0.0 | 73.9 | 2.0 | 60.1 | 0.0 |
| Nivo+ChT | 88.2 | 33.1 | 4.6 | 0.0 | 17.1 | 0.0 |
| Nivo+Ipil+ChT | 82.4 | 22.0 | 24.9 | 0.0 | 38.5 | 0.0 |
| Pemb | 51.4 | 0.8 | 56.6 | 1.4 | 53.5 | 0.0 |
| Pemb+ChT | 85.1 | 31.4 | 37.0 | 0.0 | 48.2 | 0.0 |
| Placebo | 53.3 | 9.1 | 96.0 | 76.7 | 99.5 | 95.4 |

TABLE S2 | The relevant SUCRA values for different treatment for irAEs

| Treatment | Grade 3-5 | | Grade all | |
| --- | --- | --- | --- | --- |
|  | SUCRA | PrBest (%) | SUCRA | PrBest (%) |
| ChT | 85.1 | 0.0 | 98.7 | 91.3 |
| Avel | 48.4 | 31.6 | 17.6 | 1.2 |
| Camr | 41.8 | 31.9 | 23.3 | 0.0 |
| Ipil | 56.0 | 33.0 | 10.3 | 0.5 |
| Nivo | 48.5 | 0.0 | 54.6 | 0.0 |
| Nivo+Ipil | 21.6 | 3.0 | 39.9 | 0.0 |
| Pemb | 36.4 | 0.4 | 73.6 | 0.0 |
| Placebo | 76.5 | 0.0 | 82.0 | 7.1 |

TABLE S3 | The rates of some specific immune-related adverse events of interest

| Adverse events | ICIs | | | non-ICIs | | |
| --- | --- | --- | --- | --- | --- | --- |
|  | Rate | Lci | Uci | Rate | Lci | Uci |
| skin reaction | 15.76 | 13.67 | 17.84 | 2.49 | 1.58 | 3.40 |
| hypothyroidism | 9.73 | 8.07 | 11.39 | 1.19 | 0.52 | 1.86 |
| Infusion-related reactions | 5.93 | 4.29 | 7.58 | 3.60 | 2.27 | 4.94 |
| hepatitis | 5.25 | 4.28 | 6.22 | 2.06 | 1.40 | 2.72 |
| pneumonitis | 4.45 | 3.50 | 5.40 | 0.38 | 0.08 | 0.69 |
| hyperthyroidism | 3.52 | 2.46 | 4.57 | 0.52 | 0.07 | 0.98 |
| interstitial lung disease | 1.86 | 0.78 | 3.11 | 0.00 | 0.00 | 0.51 |
| Colitis | 1.02 | 0.44 | 1.59 | 0.62 | 0.13 | 1.12 |
| Hypophysitis | 0.64 | 0.16 | 1.22 | 0.00 | 0.00 | 0.26 |
| Myocarditis | 0.55 | 0.00 | 1.32 | 0.00 | 0.00 | 0.36 |
| Thyroiditis | 0.36 | 0.00 | 0.86 | 0.00 | 0.00 | 0.30 |
| Type 1 diabetes mellitus | 0.33 | 0.00 | 0.92 | 0.00 | 0.00 | 0.33 |
| Grade 3-5 irAEs | 7.35 | 6.23 | 8.47 | 2.25 | 1.58 | 2.92 |
| irAEs allgrade | 44.46 | 42.47 | 46.44 | 11.09 | 9.76 | 12.41 |

Lci: Lower limit of 95% confidence interval, Uci: Upper 95% confidence interval.


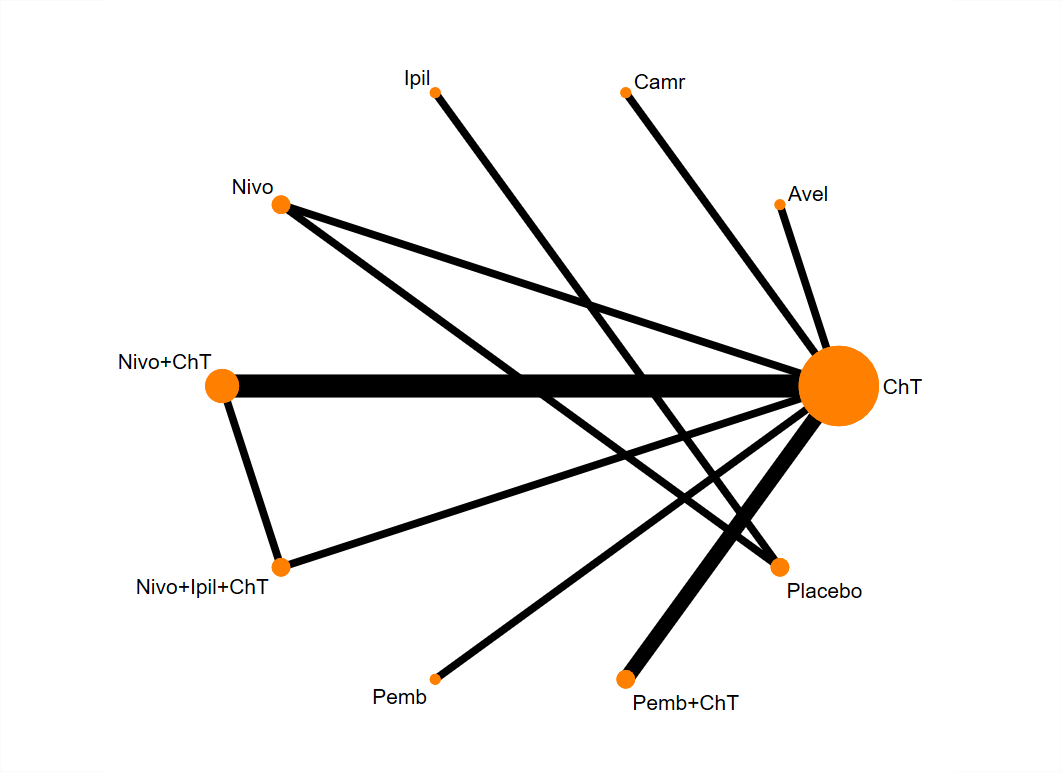


FIGURE S1 | Network plots of comparisons for grade 1-2 trAEs based network meta-analyses.

ChT, chemotherapy; Avel, avelumab; Camr, camrelizumab; Ipil, ipilimumab; Nivo, nivolumab; Pemb, pembrolizumab.

FIGURE S2A


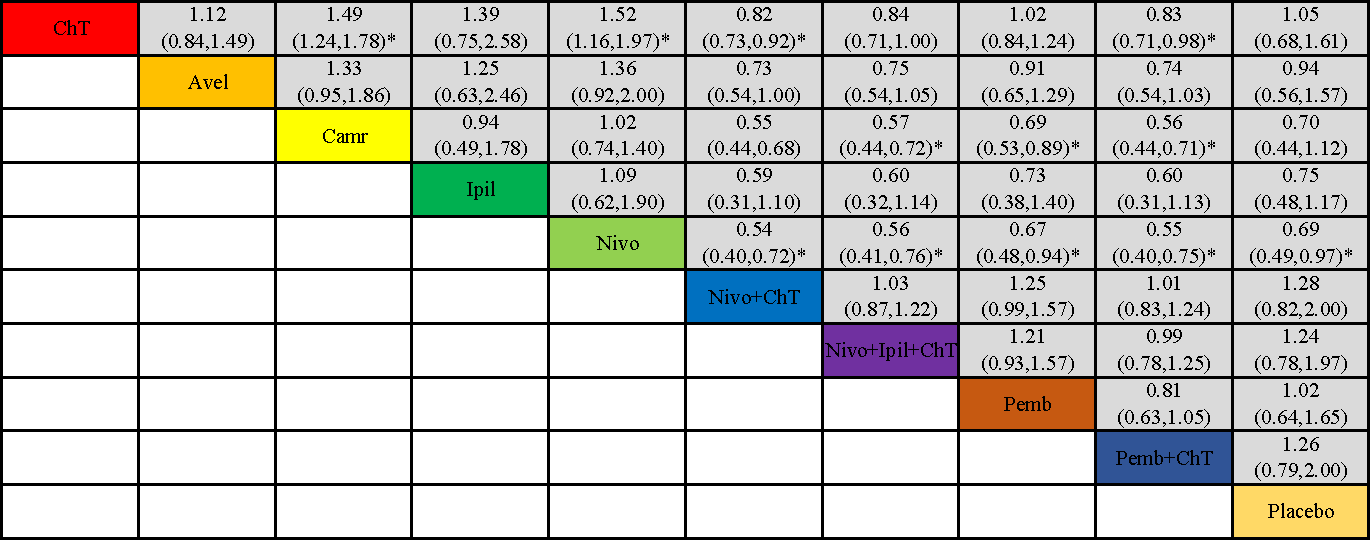


FIGURE S2B


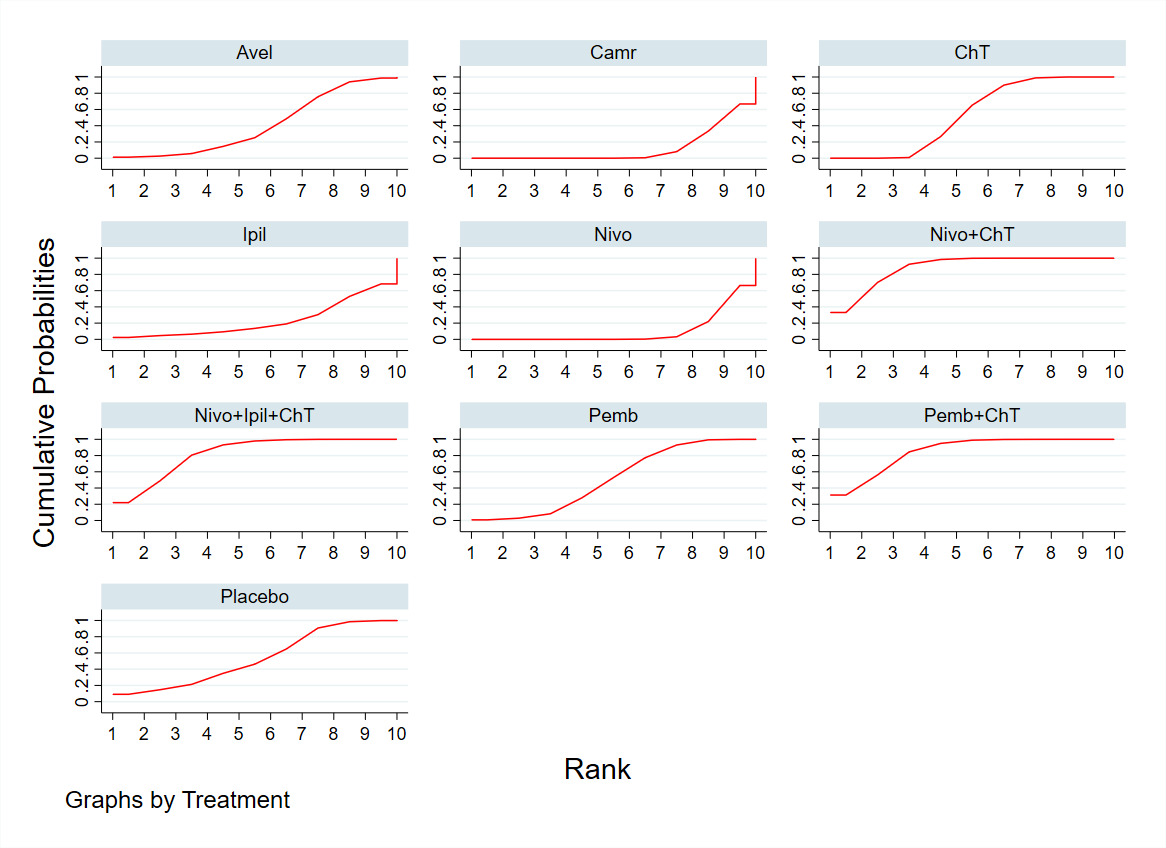


FIGURE S2 | Results of the network meta-analysis for 10 treatment regimens in terms of treatment-related adverse events(trAEs) with grade 1-2 trAEs

(A) League table for different treatment regimens. ChT=chemotherapy; Avel=avelumab; Camr=Camrelizumab; Ipil= ipilimumab; Nivo=nivolumab; Pemb=pembrolizumab.Placebo also involves the best supportive care. (B) The surface under the cumulative ranking curves (SUCRA) for grade 3-5 trAEs and all grade trAEs.


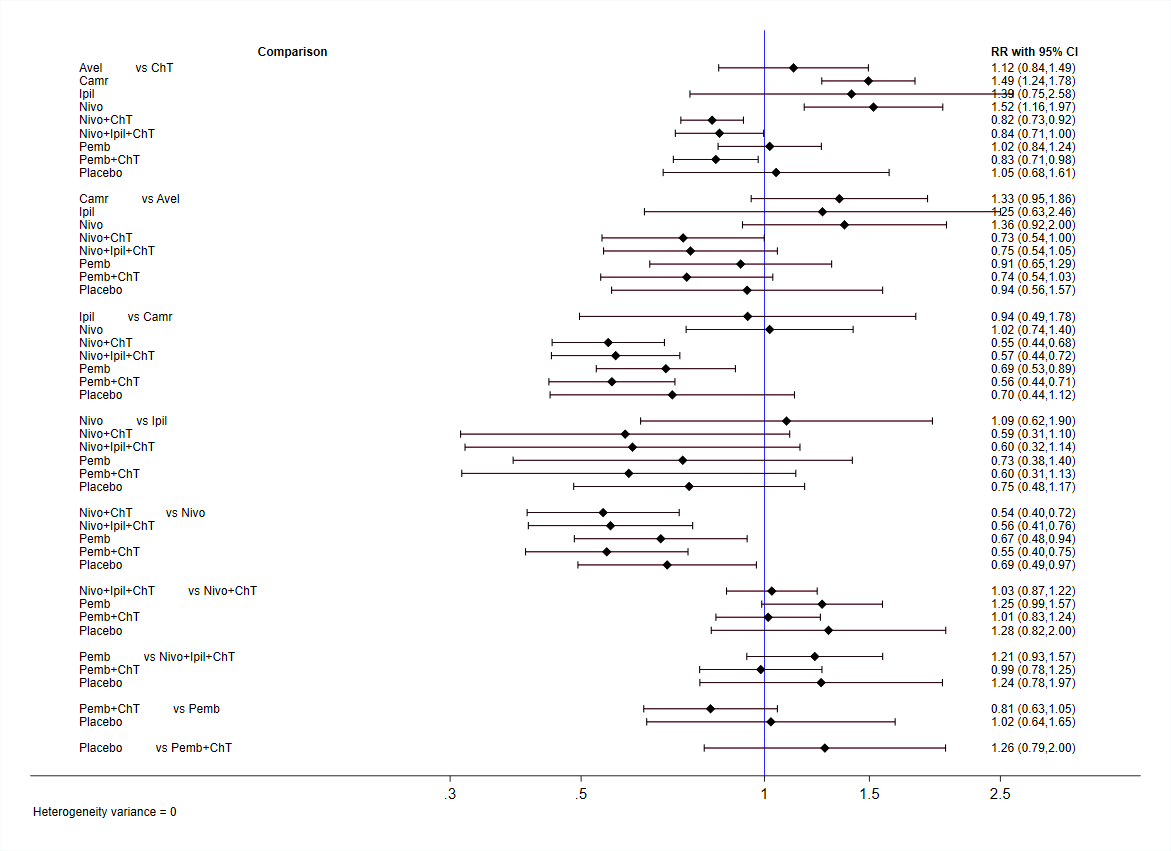


FIGURE S3 | Forestplots for pairwise comparisons of all individual regimens with each other for grade 1-2 trAEs.

FIGURE S4A


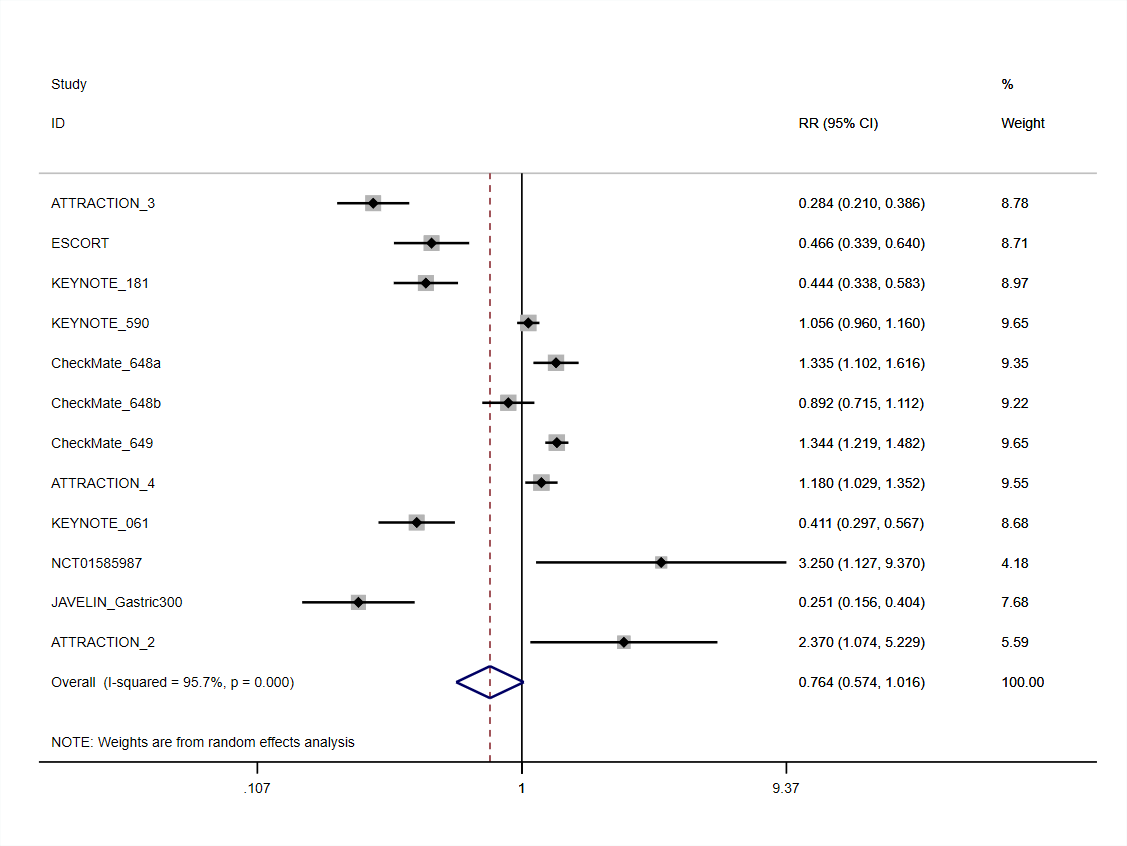


FIGURE S4B


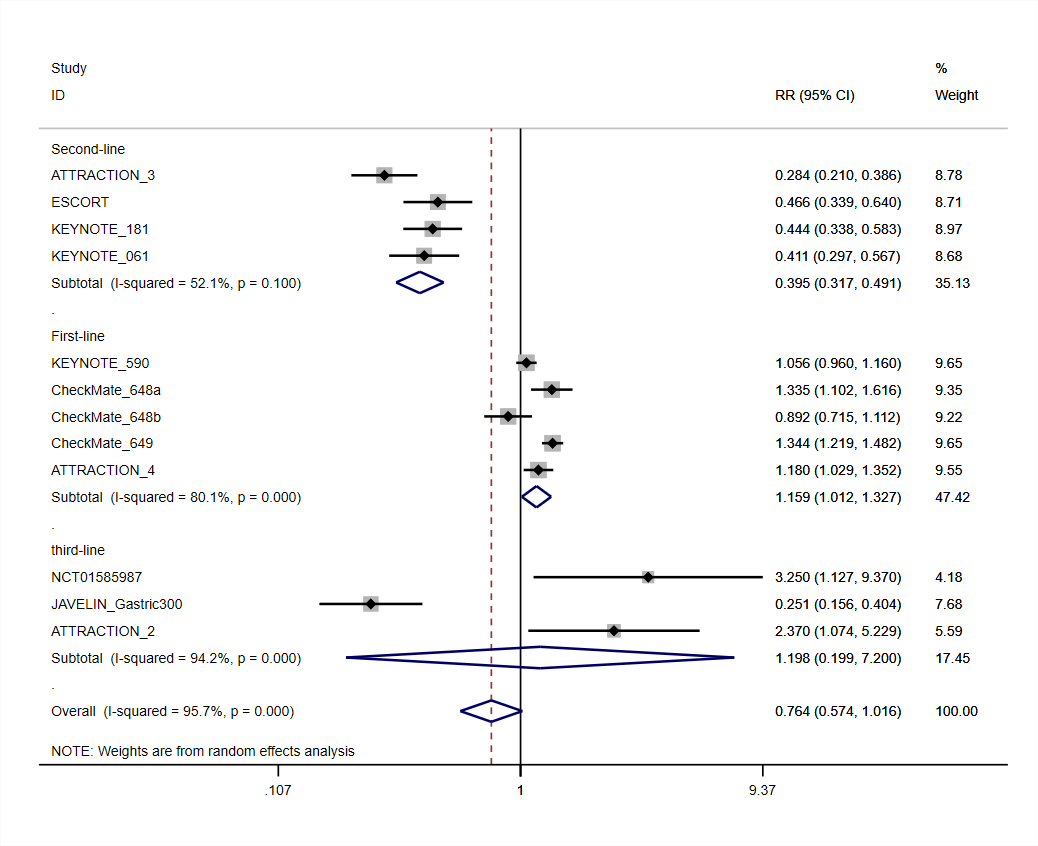


FIGURE S4C


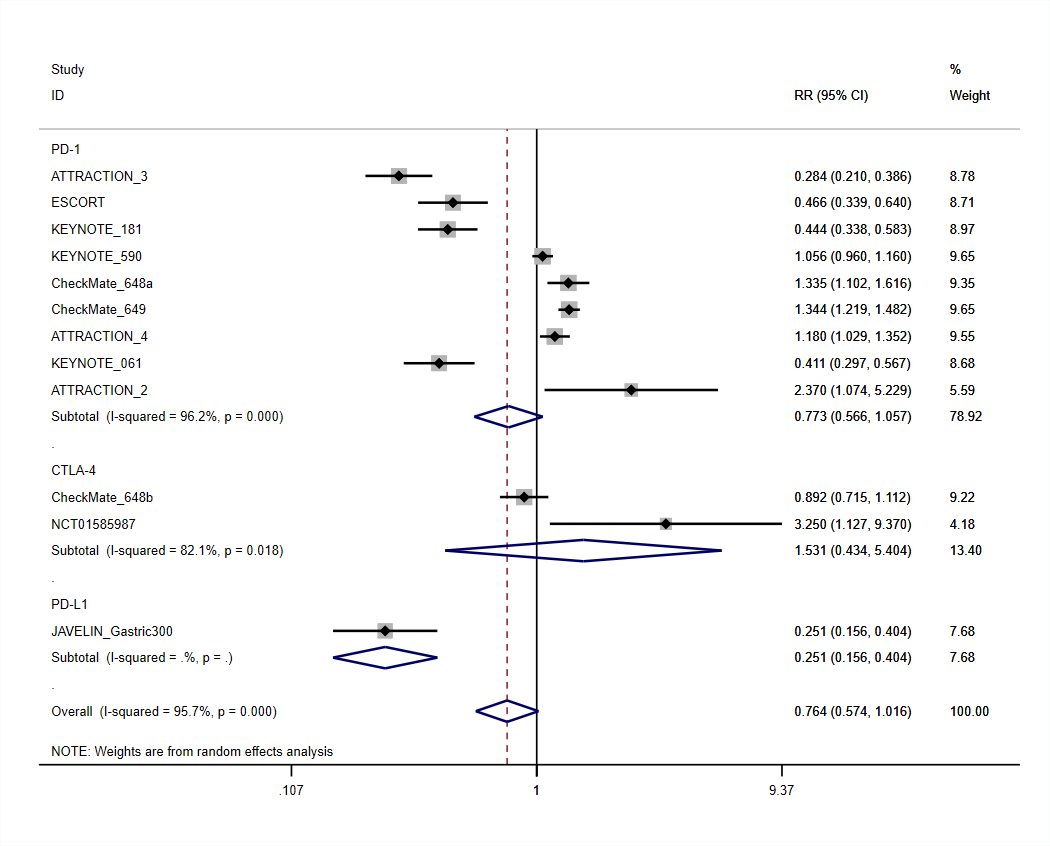


FIGURE S4D


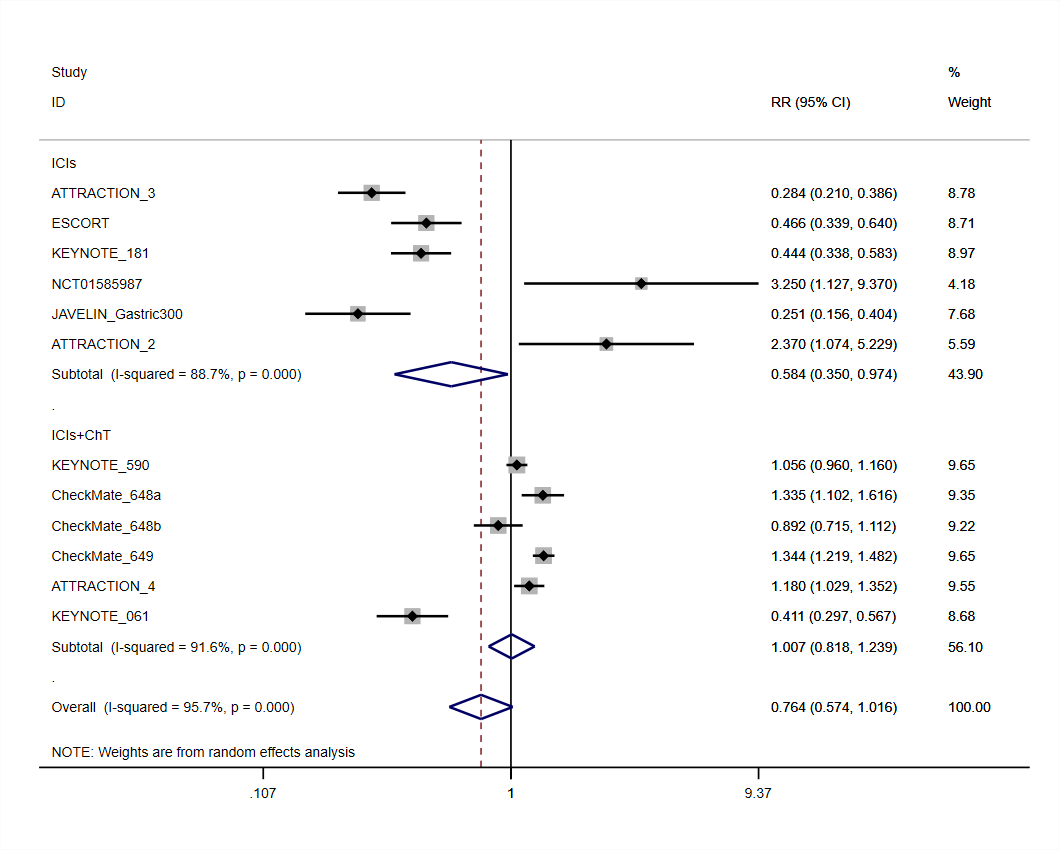


FIGURE S4E


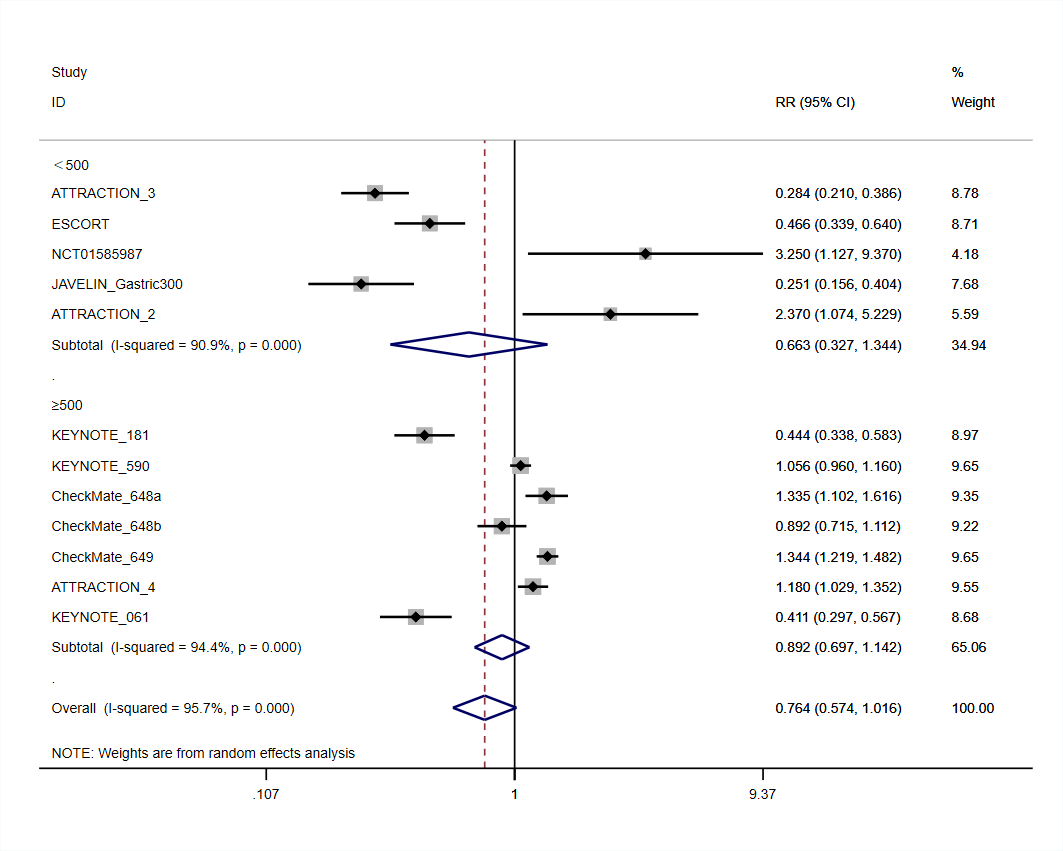


FIGURE S4 | Forestplots for traditional pairwise meta-analysis for grade 3-5 trAEs with (A) total, (B) subgrouped by treatment lines, (C) subgrouped by ICIs drugs type, (D) subgrouped by treatment mode and (E) subgrouped by sample size.

FIGURE S5A


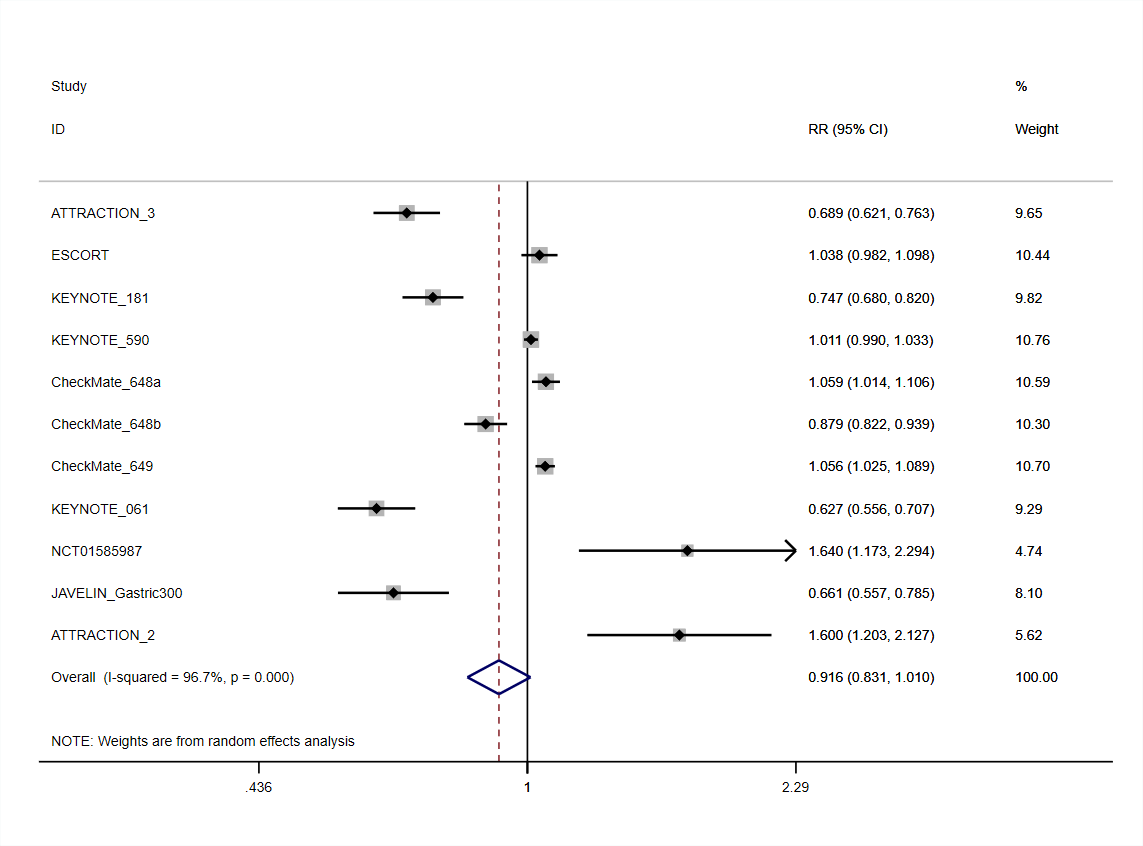


FIGURE S5B


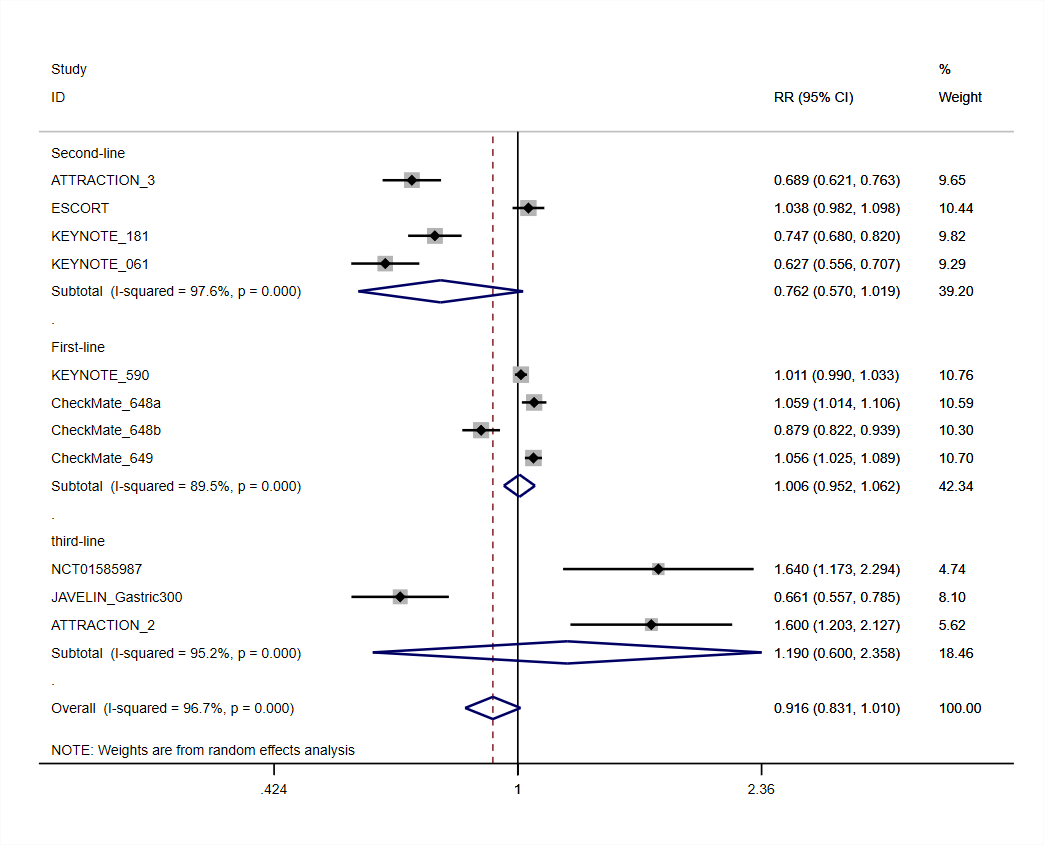


FIGURE S5C


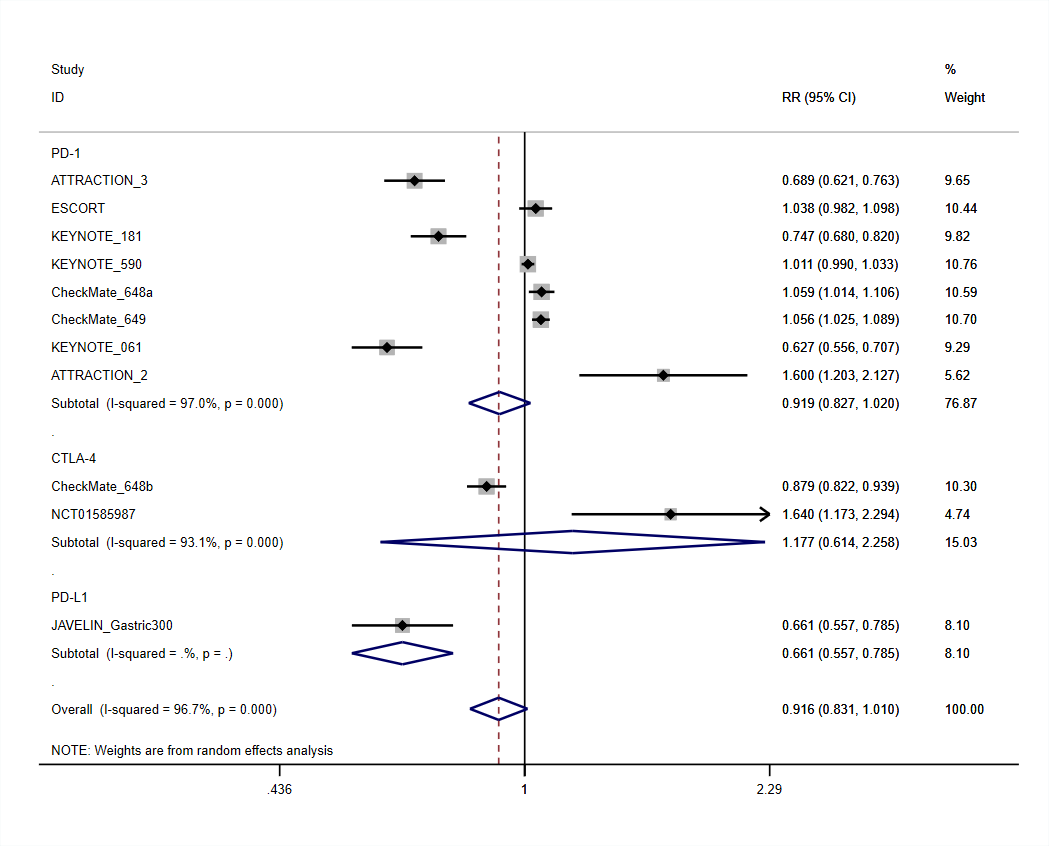


FIGURE S5D


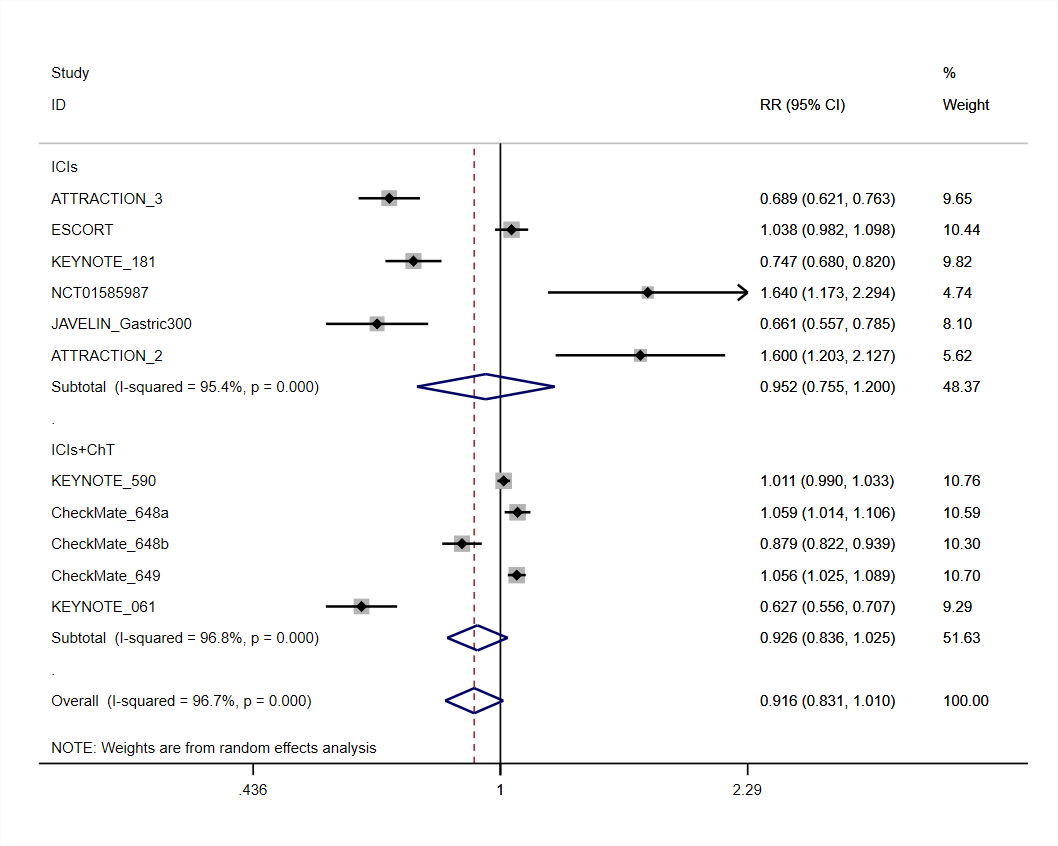


FIGURE S5E


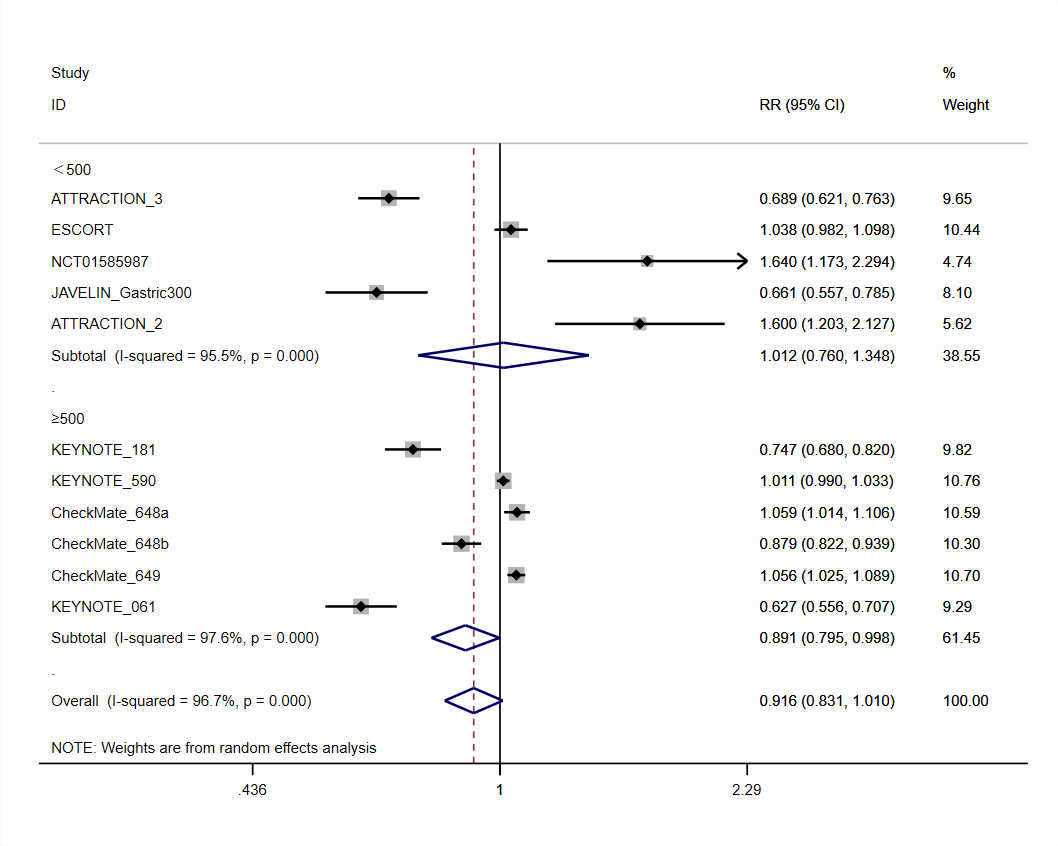


FIGURE S5 | Forestplots for traditional pairwise meta-analysis for grade 3-5 trAEs with (A) total, (B) subgrouped by treatment lines, (C) subgrouped by ICIs drugs type, (D) subgrouped by treatment mode and (E) subgrouped by sample size.

FIGURE S6A


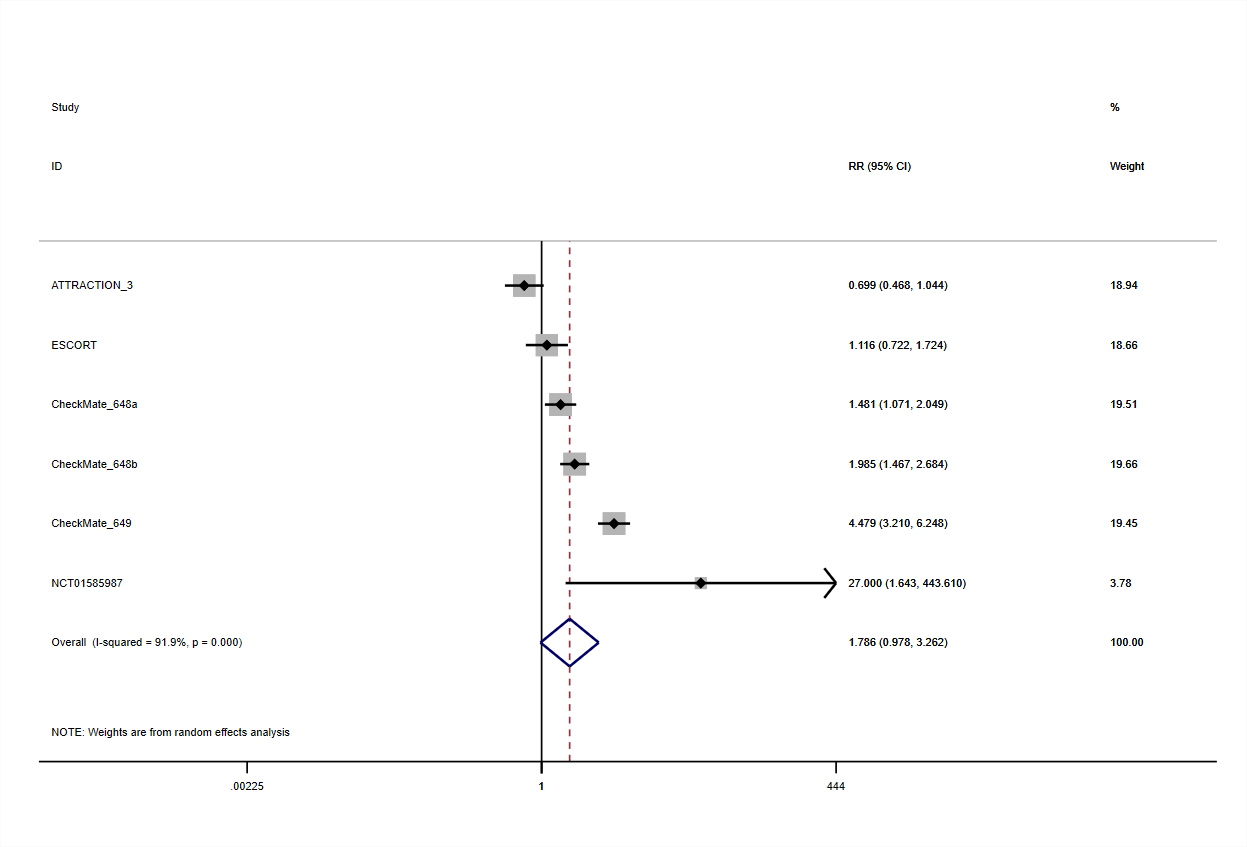


FIGURE S6B


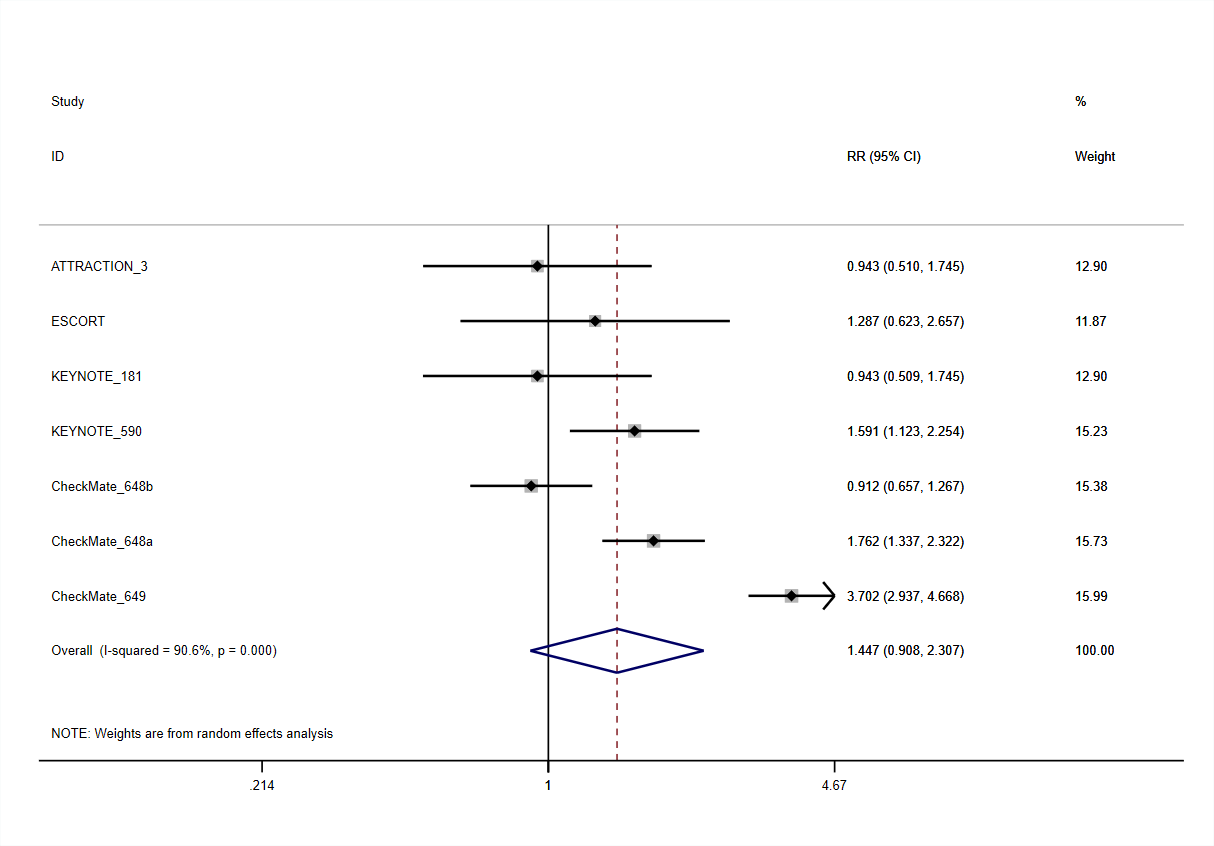


FIGURE S6C


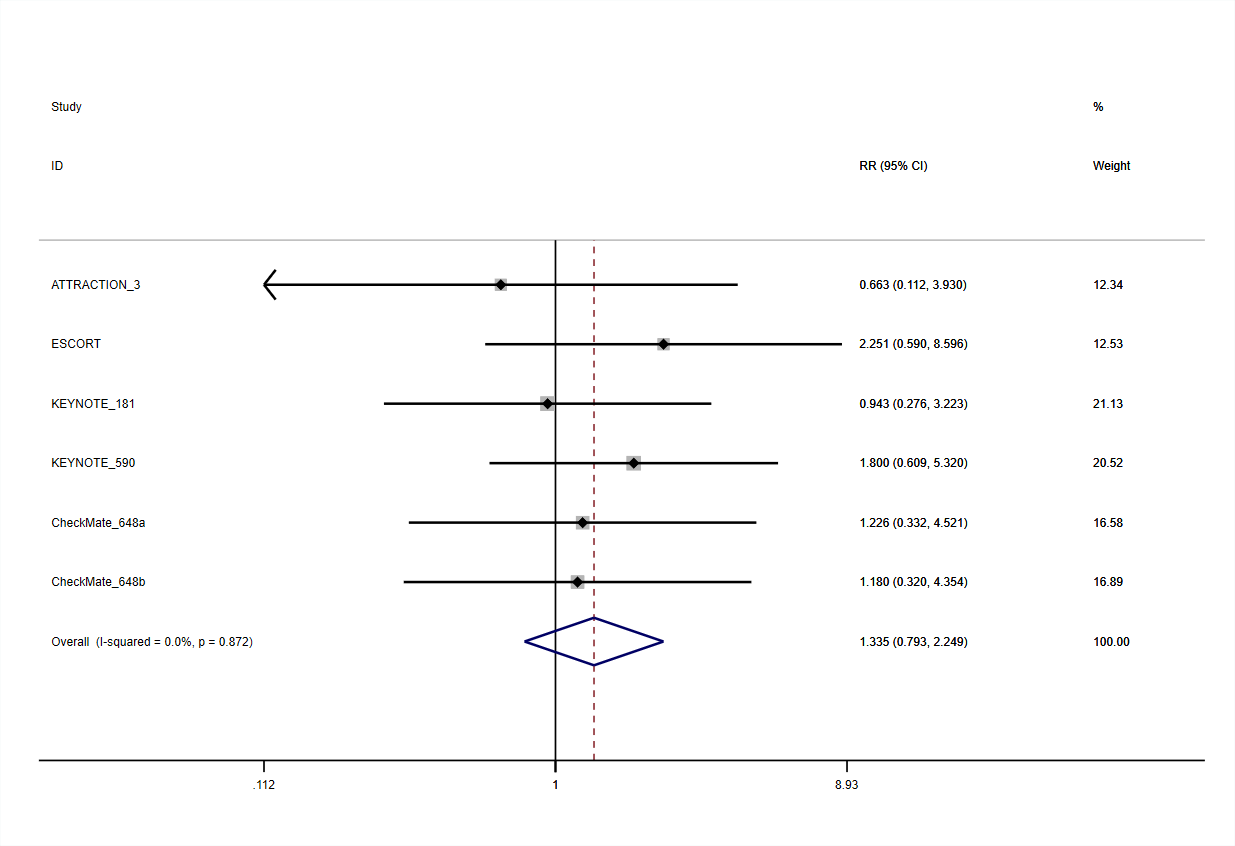


FIGURE S6 | Forestplots for serious outcome with (A) forestplots for serious trAEs, (B) forestplots for events leading to discontinuation and (C) forestplots for treatment-related death.

The CheckMate 648 study is a three-arm trial, so the comparison pairs were split into CheckMate_648a and CheckMate_648b.

FIGURE S7A


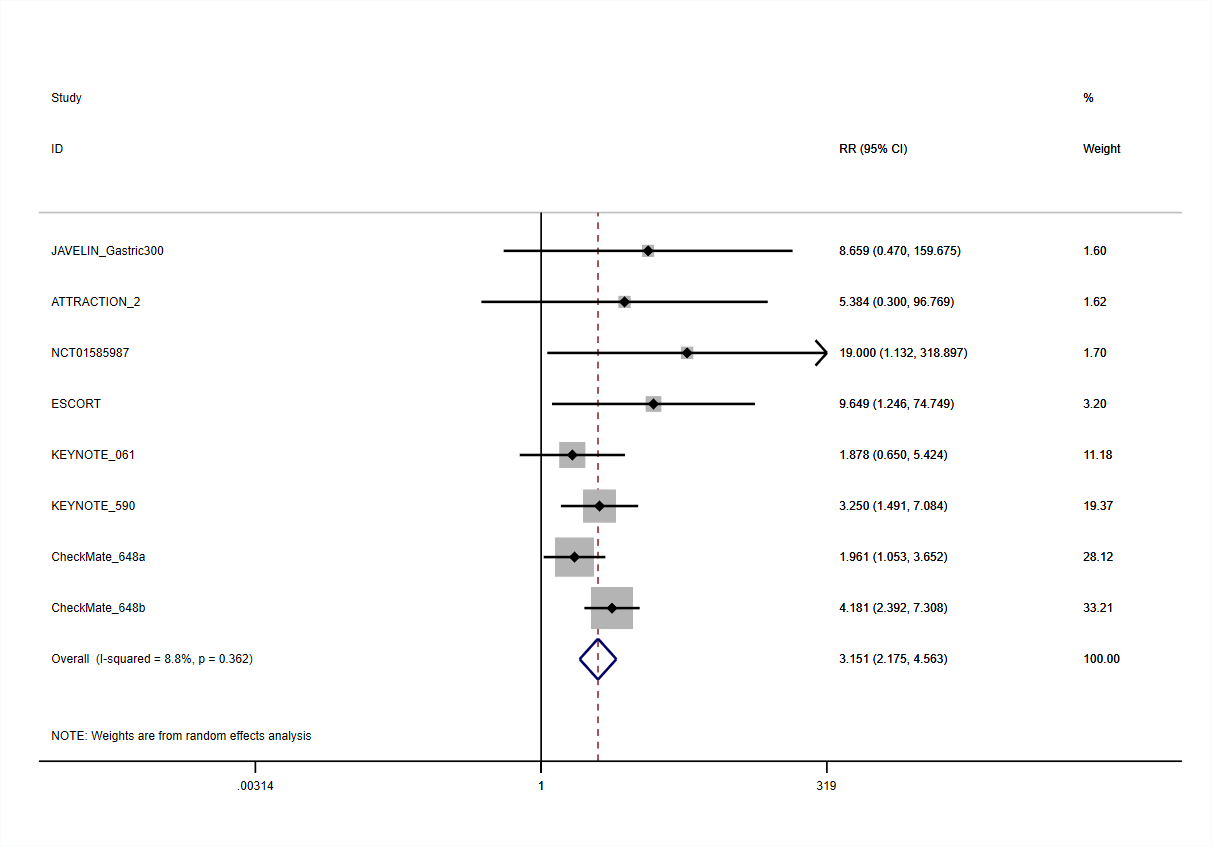


FIGURE S7B


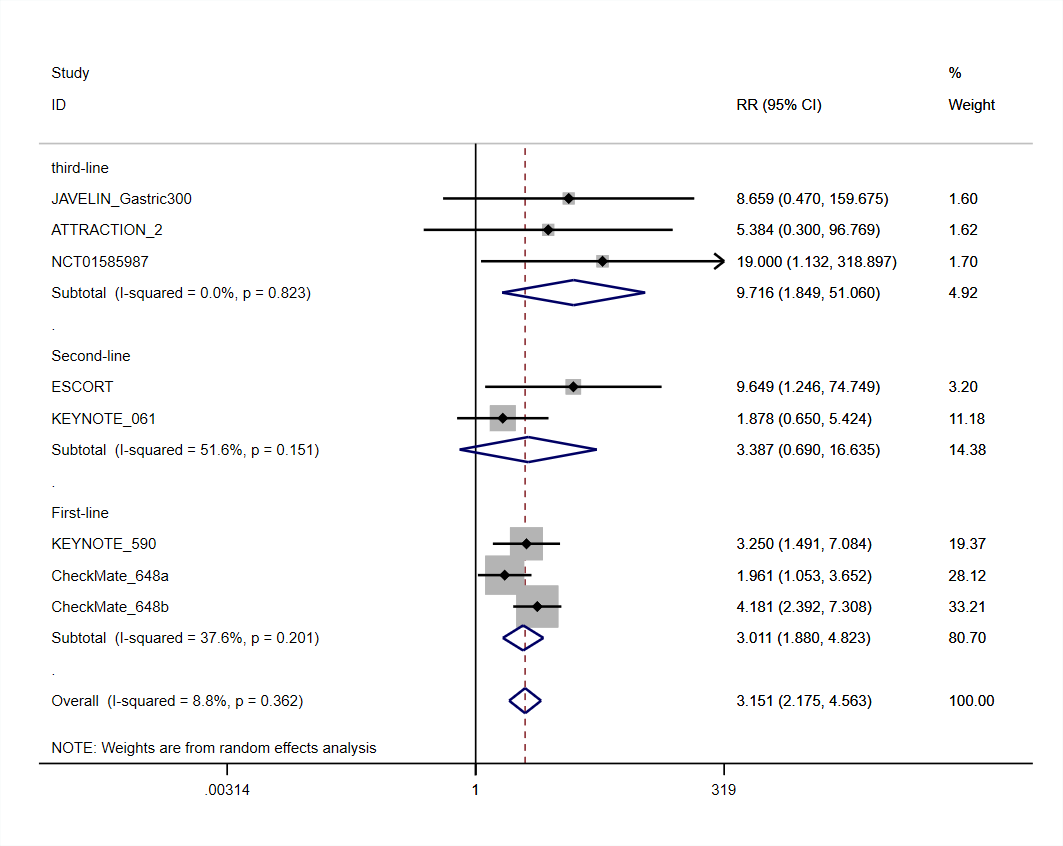


FIGURE S7C


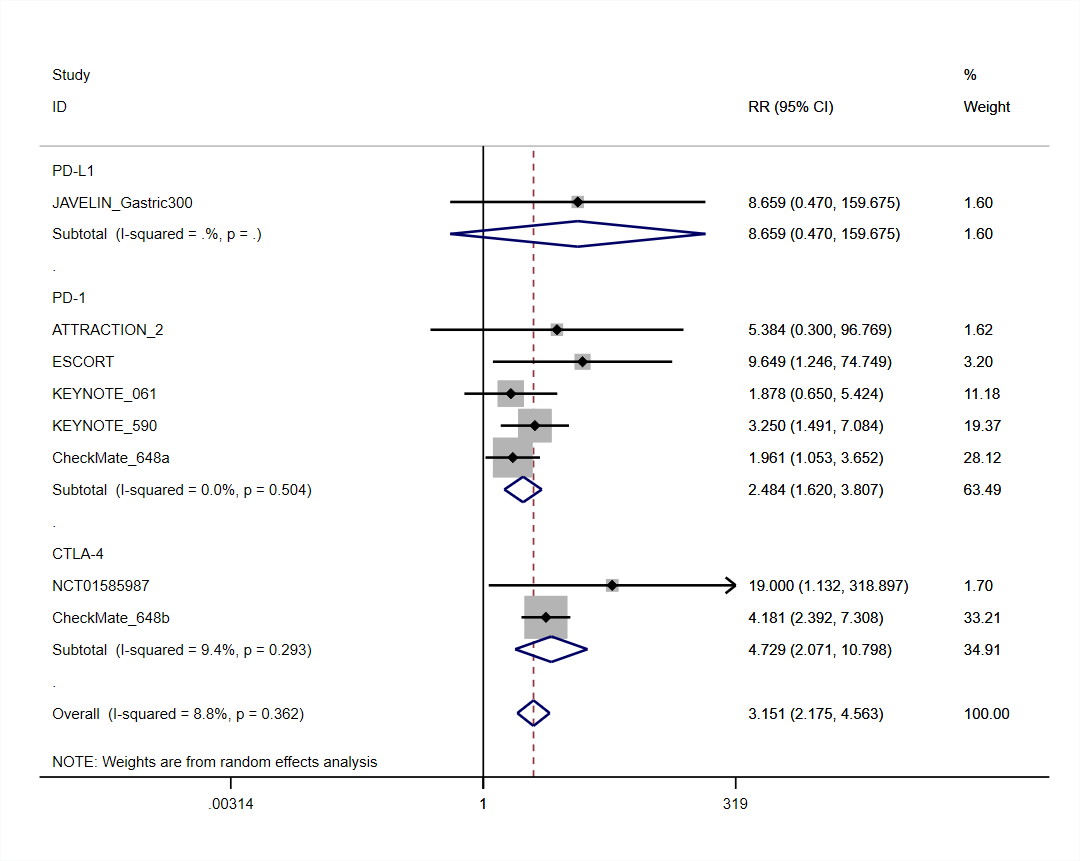


FIGURE S7D


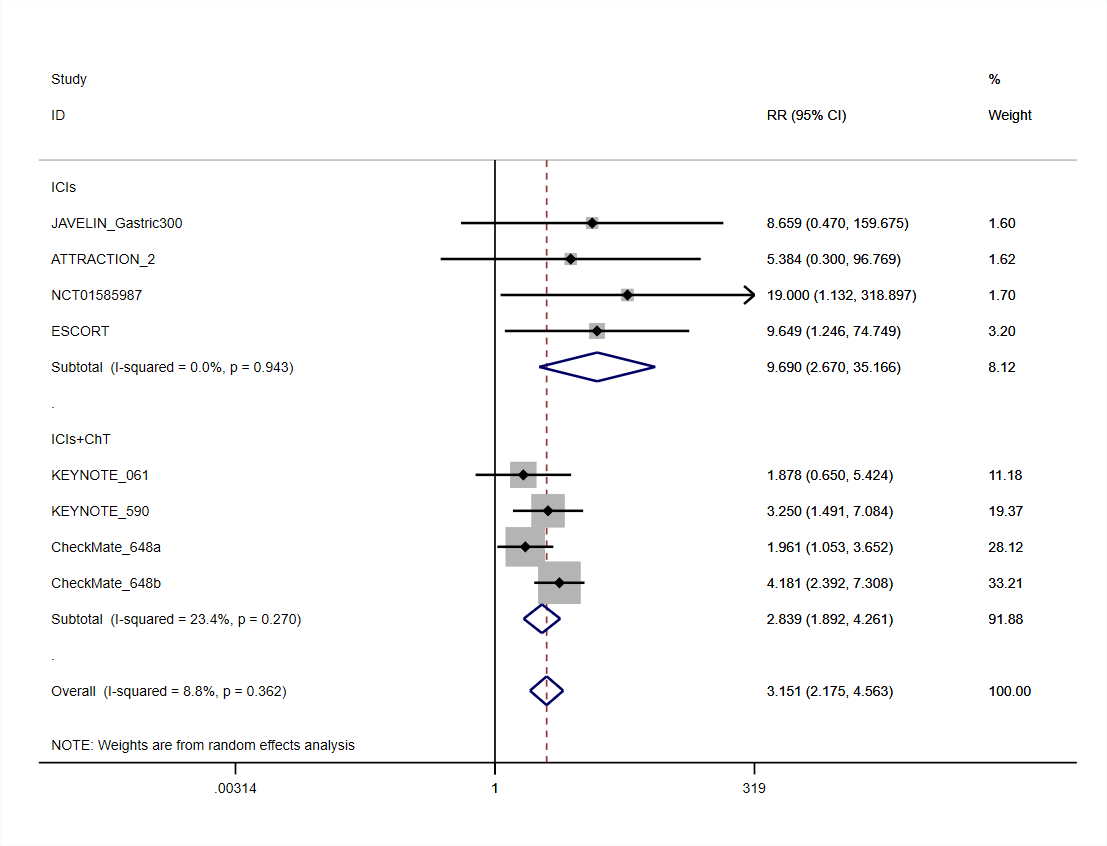


FIGURE S7E


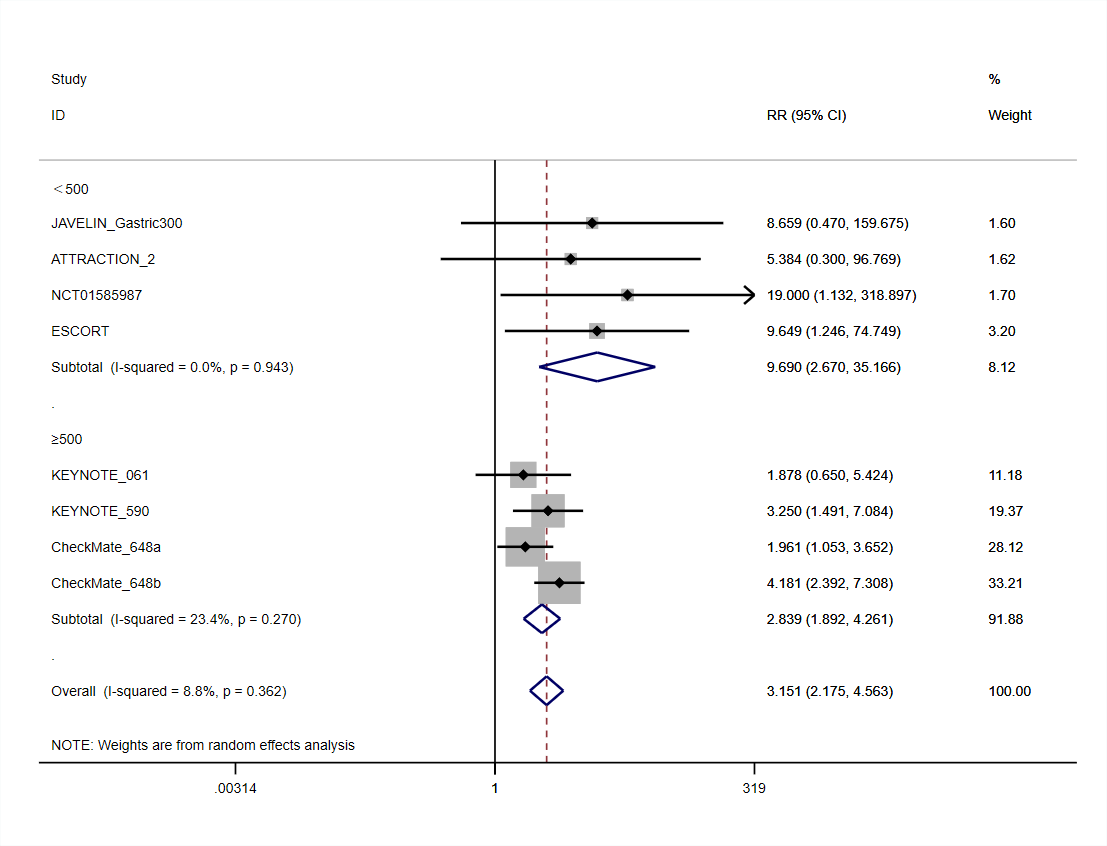


FIGURE S7 | Forestplots for traditional pairwise meta-analysis for grade 3-5 irAEs with (A) total, (B) subgrouped by treatment lines, (C) subgrouped by ICIs drugs type, (D) subgrouped by treatment mode and (E) subgrouped by sample size.

FIGURE S8A


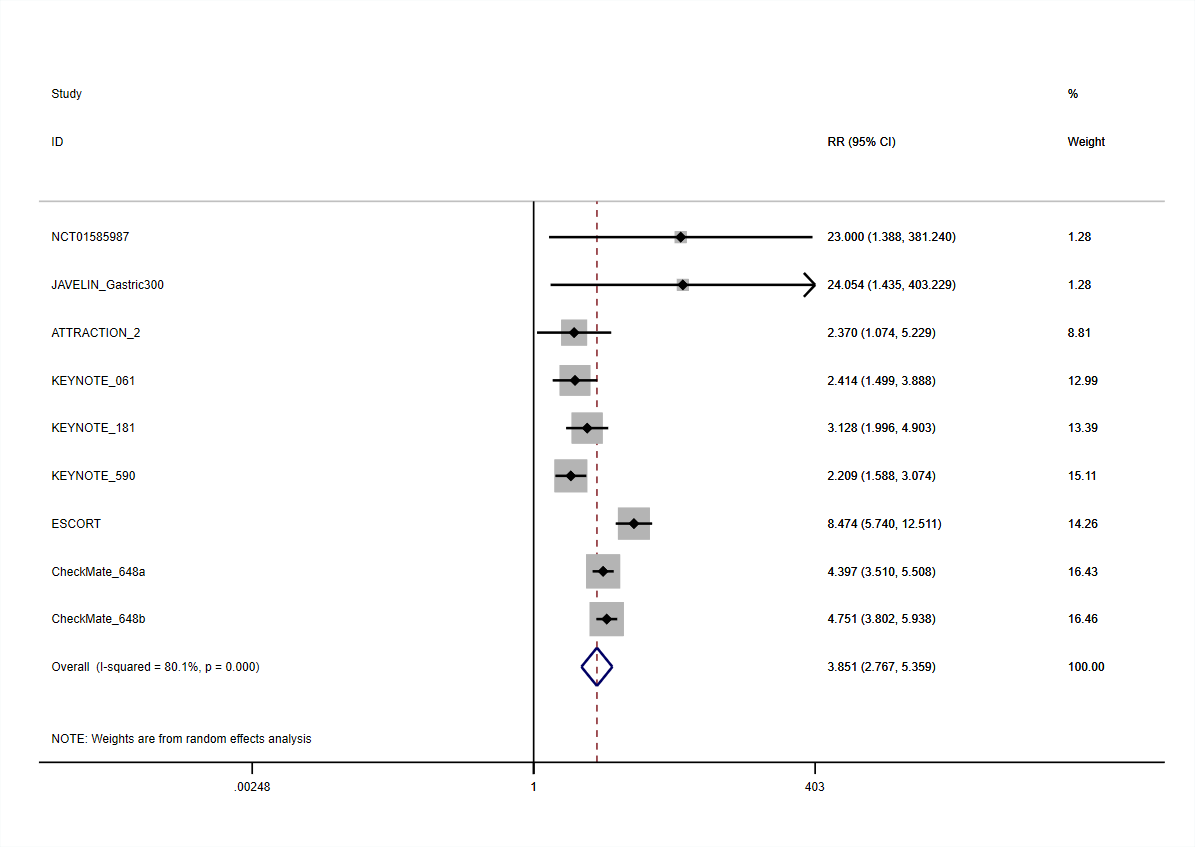


FIGURE S8B


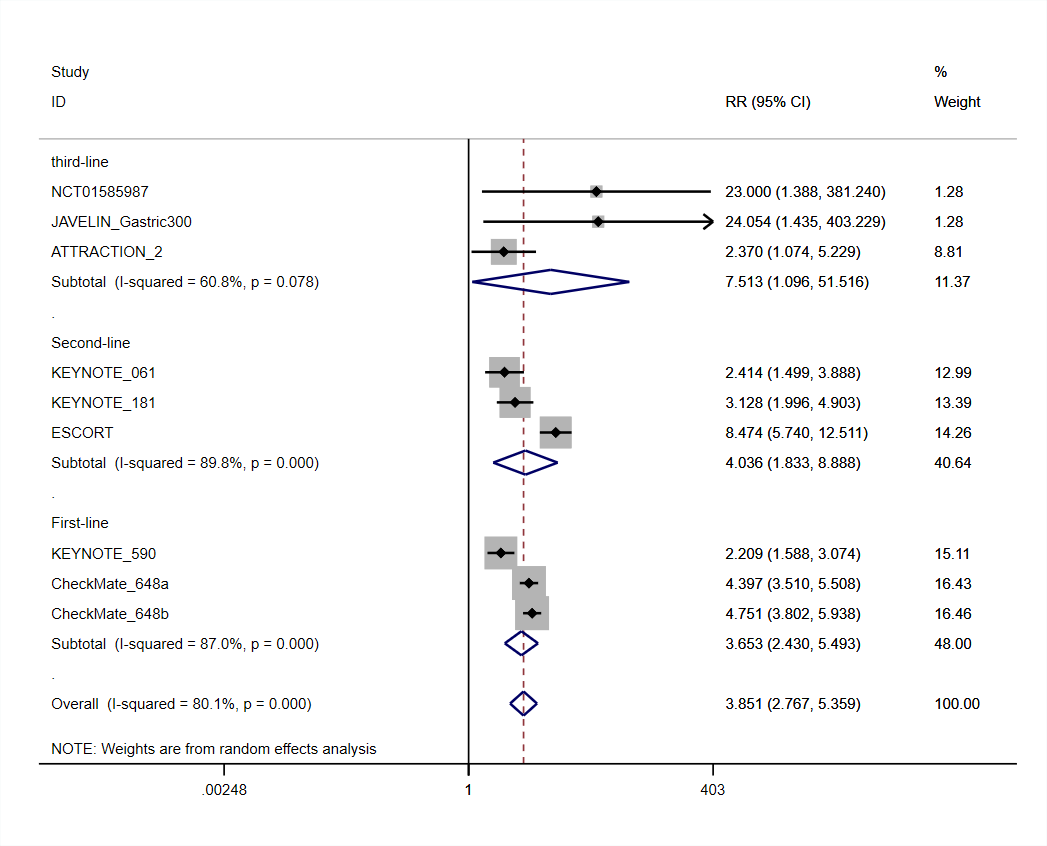


FIGURE S8C


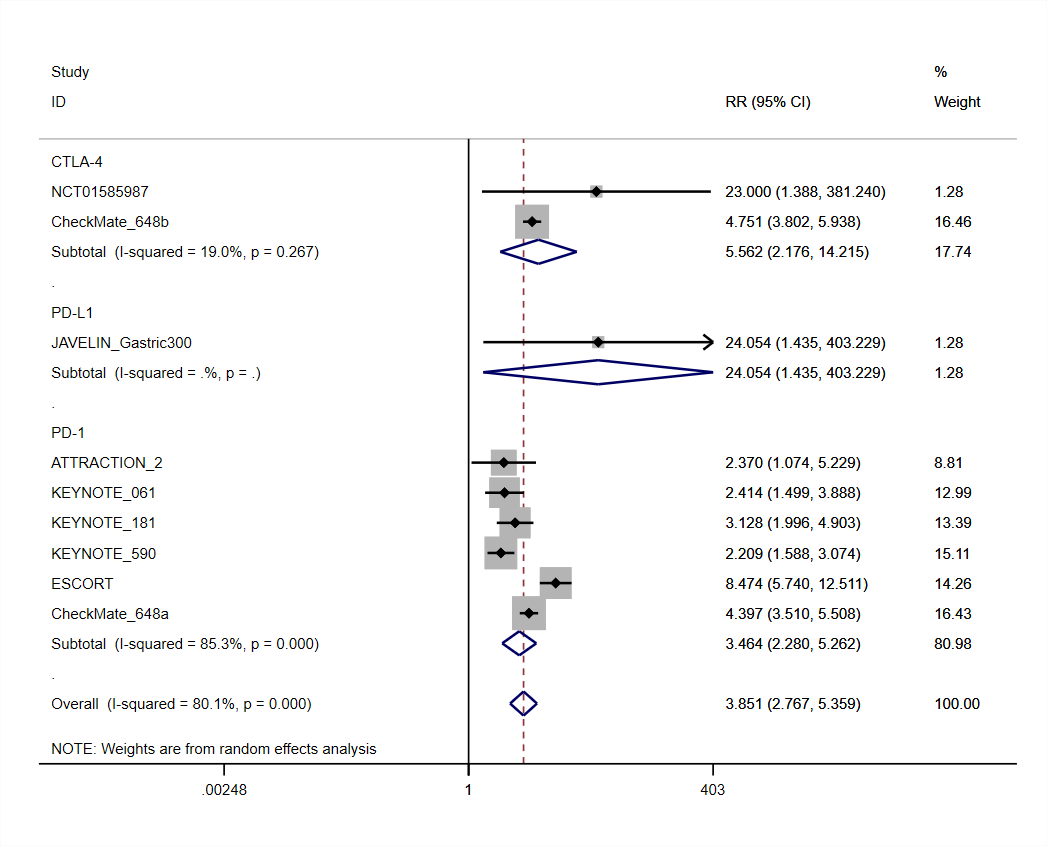


FIGURE S8D


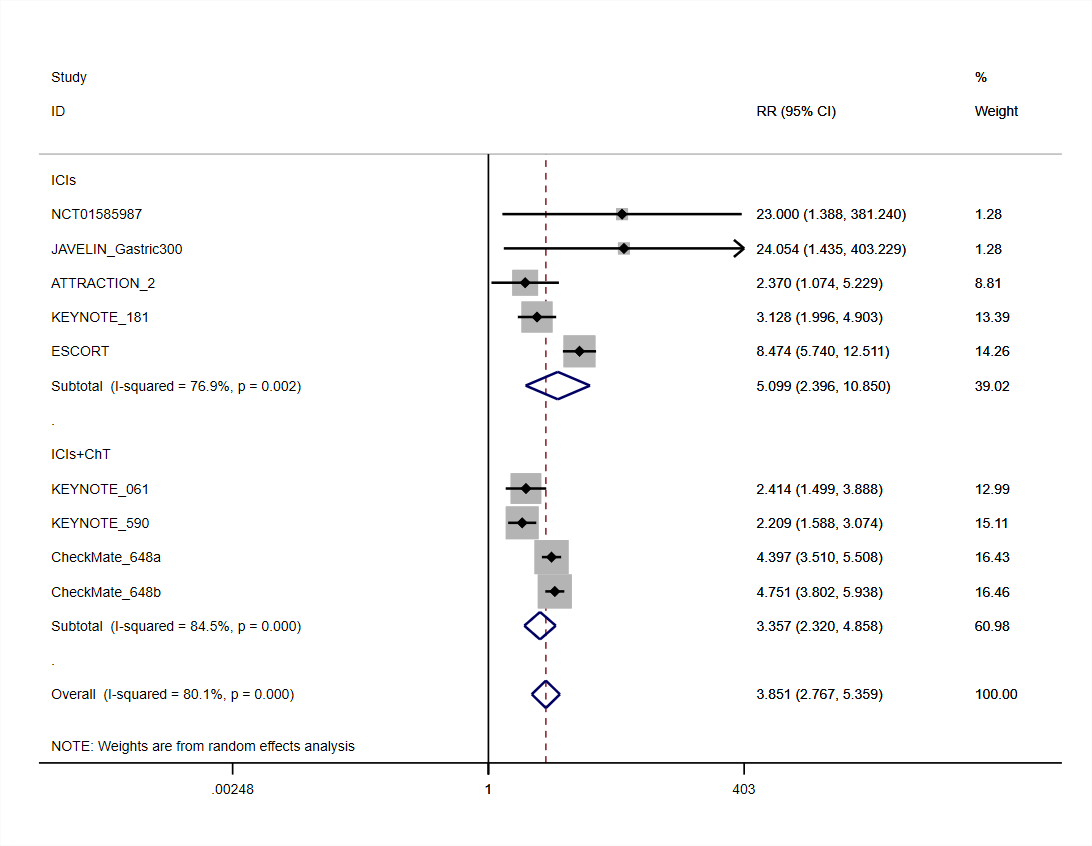


FIGURE S8E


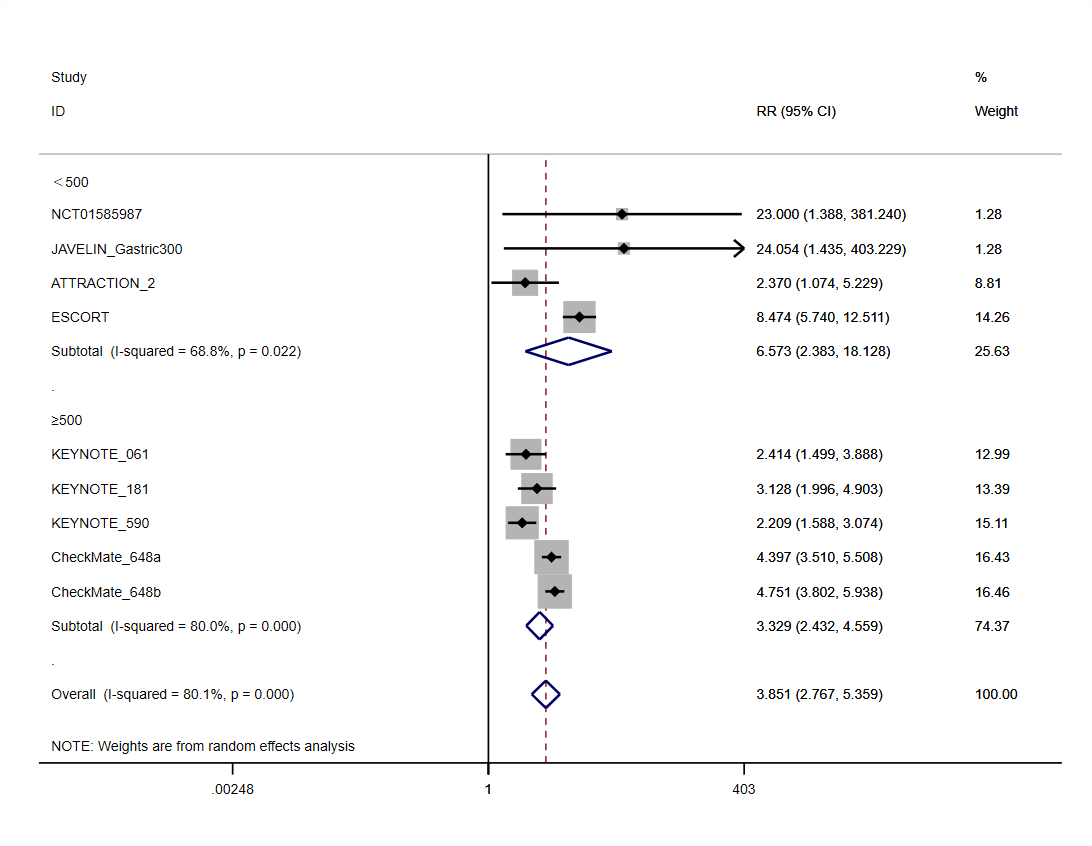


FIGURE S8 | Forestplots for traditional pairwise meta-analysis for grade 3-5 irAEs with (A) total, (B) subgrouped by treatment lines, (C) subgrouped by ICIs drugs type, (D) subgrouped by treatment mode and (E) subgrouped by sample size.
