# Supplementary figures and images for: Treatment- and immune-related adverse events of immune checkpoint inhibitors in esophageal or gastroesophageal junction cancer: A network meta-analysis of randomized controlled trials
Source: Front Oncol. 2022 Dec 8;12:821626. doi: 10.3389/fonc.2022.821626 (PMC9780048; doi:10.3389/fonc.2022.821626)

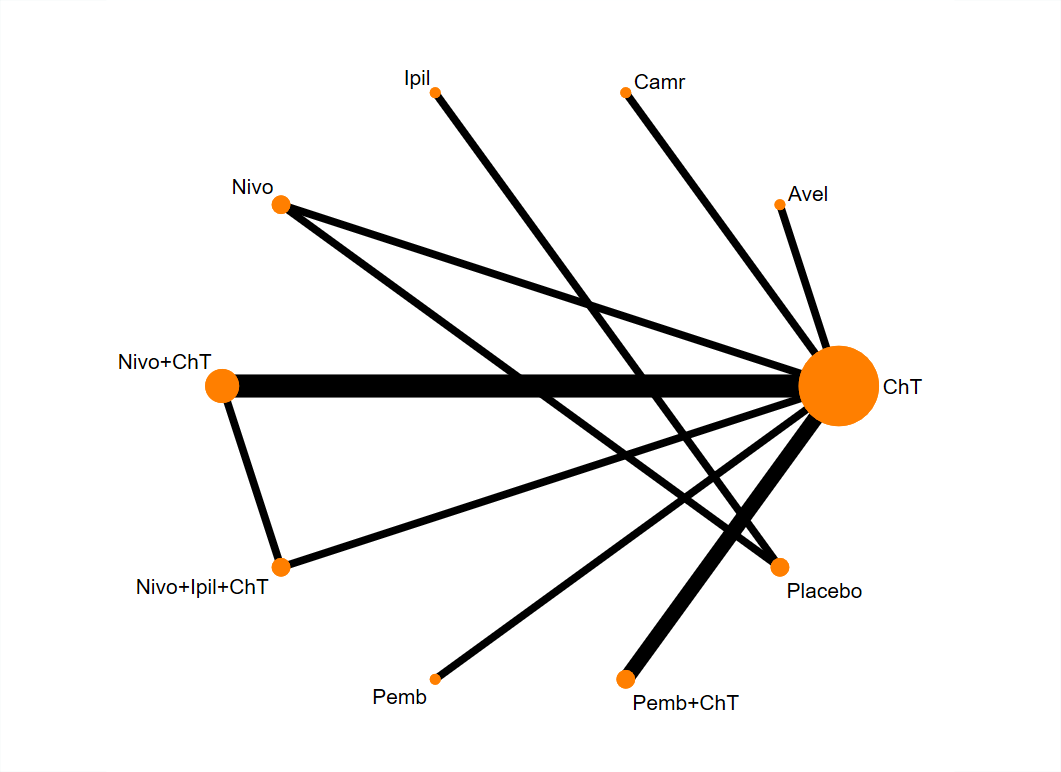

Supplement: Supplementary file 1 [file DataSheet_1.zip › Supplementary figures and tables/FIGURE S1 Network plots of grade 1-2 trAEs.tif]

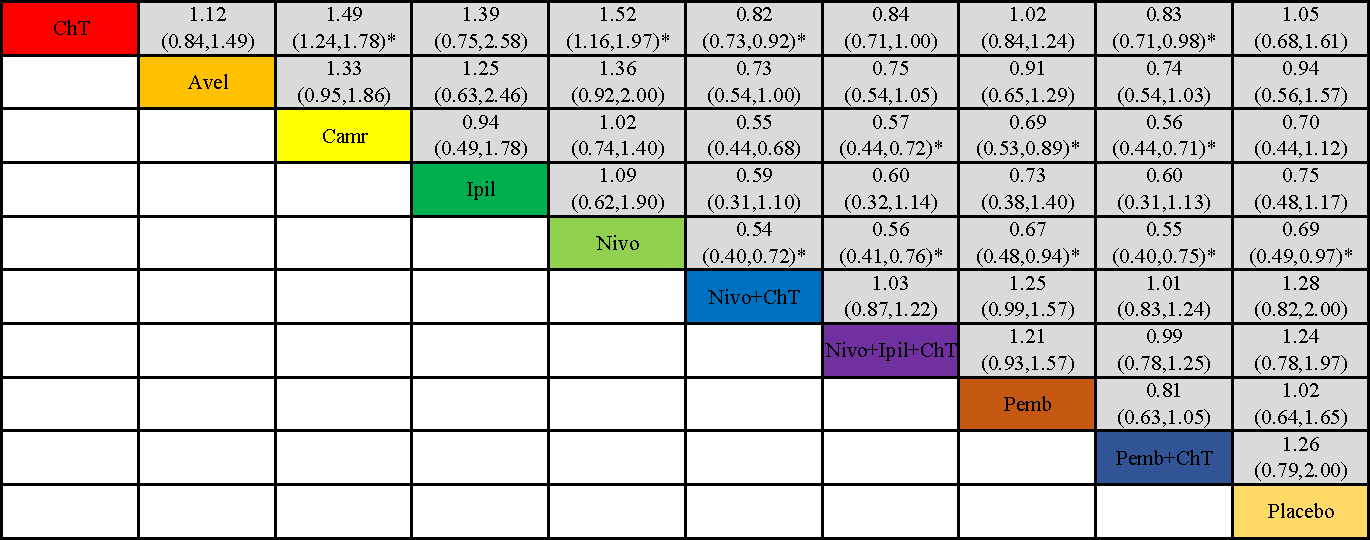

Supplement: Supplementary file 1 [file DataSheet_1.zip › Supplementary figures and tables/FIGURE S2A Results of the network meta-analysis for grade 1-2 trAEs.tiff]

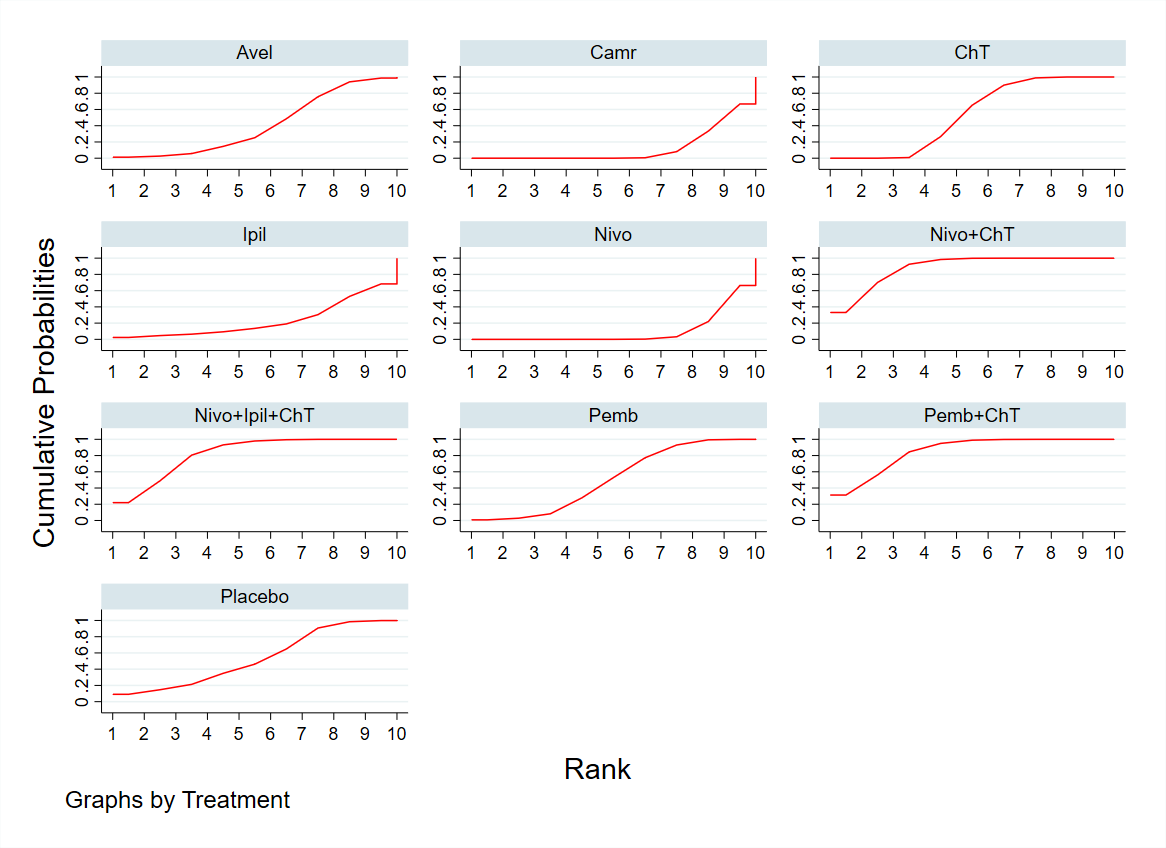

Supplement: Supplementary file 1 [file DataSheet_1.zip › Supplementary figures and tables/FIGURE S2B The surface under the cumulative ranking curves (SUCRA) for grade 1-2 trAEs .tif]

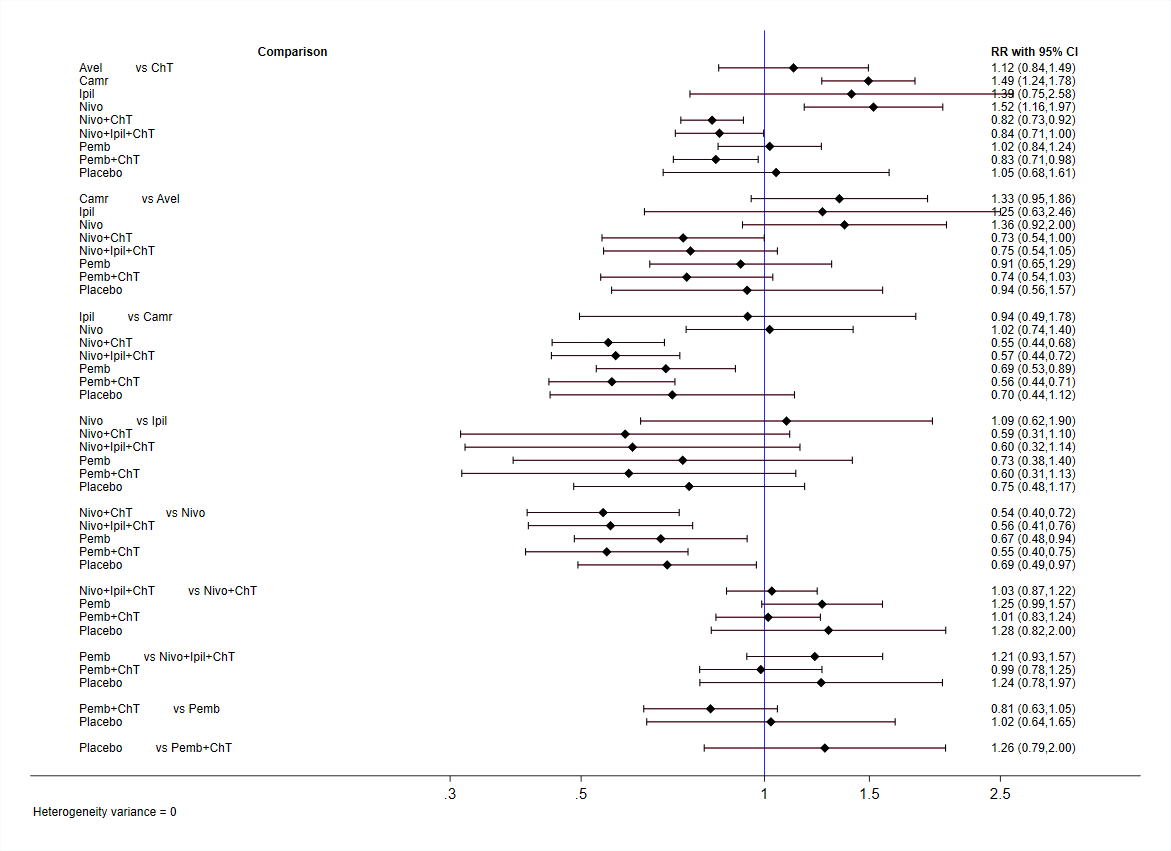

Supplement: Supplementary file 1 [file DataSheet_1.zip › Supplementary figures and tables/FIGURE S3 Forestplots for pairwise comparisons of all individual regimens with each other for grade 1-2 trAEs.tif]

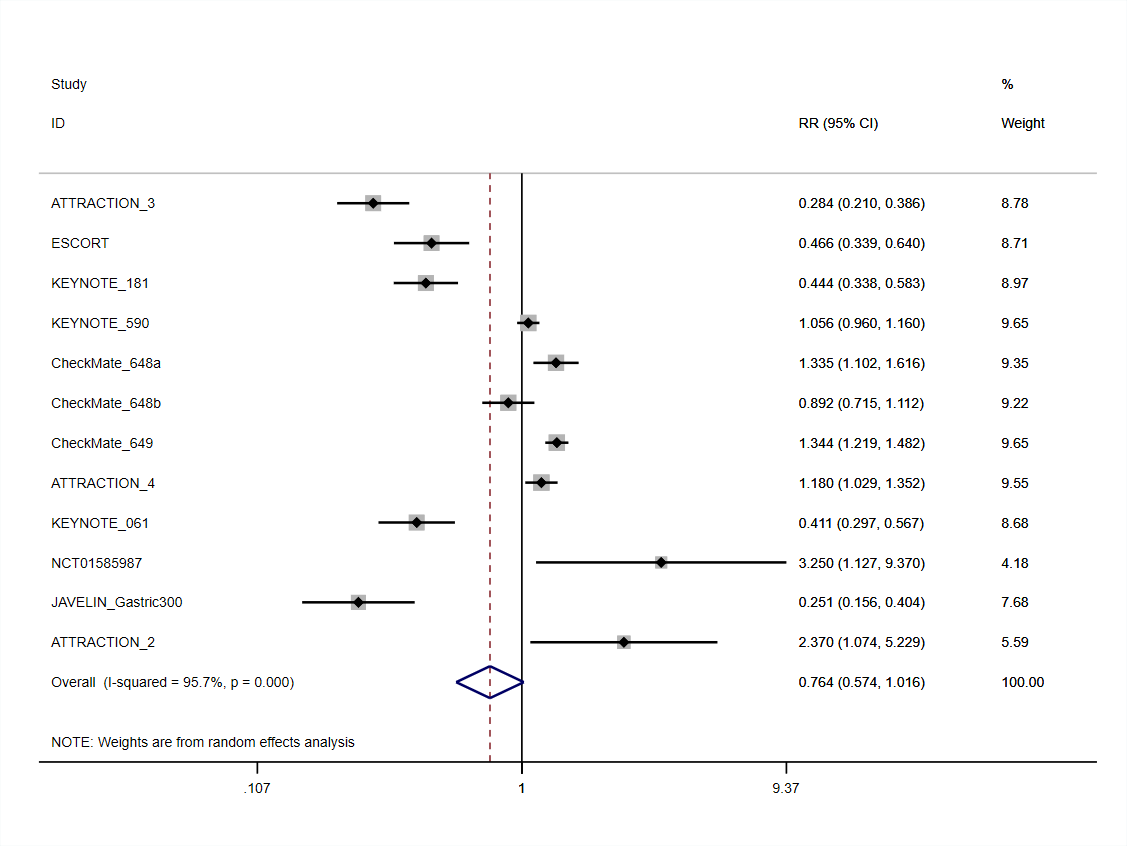

Supplement: Supplementary file 1 [file DataSheet_1.zip › Supplementary figures and tables/FIGURE S4A Subgroup forestplots for traditional pairwise meta-analysis for grade 3-5 trAEs.tif]

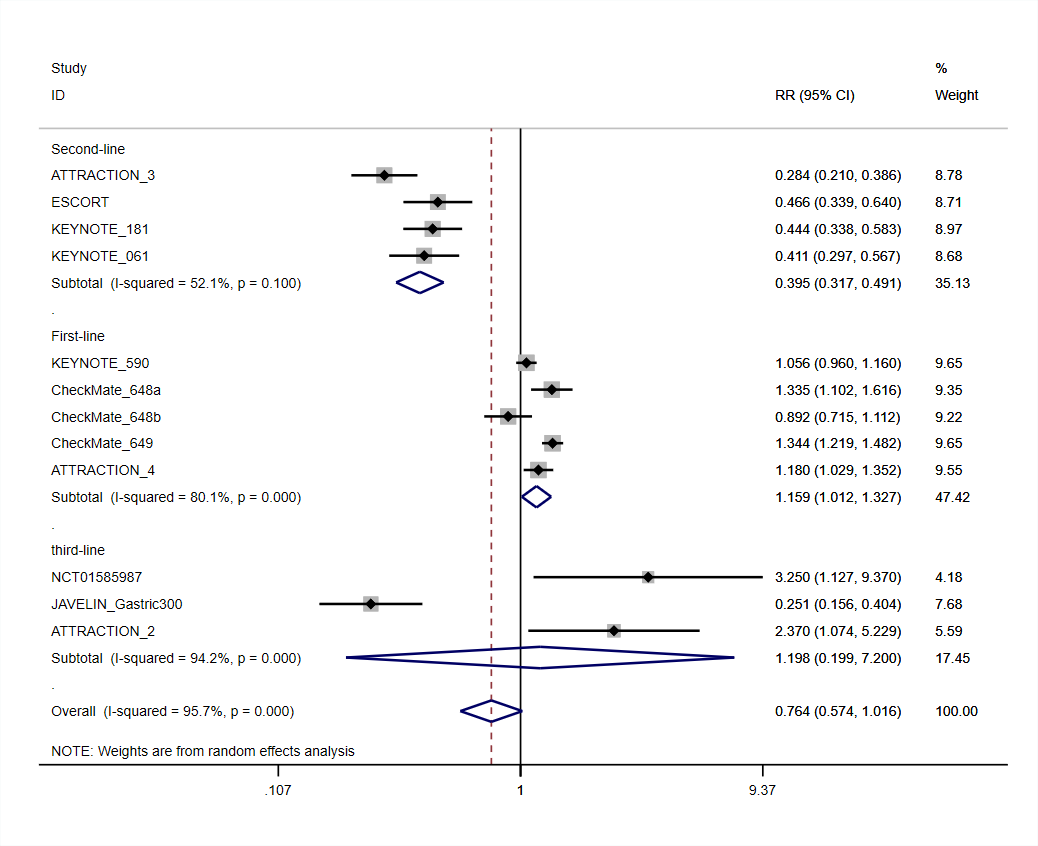

Supplement: Supplementary file 1 [file DataSheet_1.zip › Supplementary figures and tables/FIGURE S4B Forestplots for traditional pairwise meta-analysis for grade 3-5 trAEs subgrouped by treatment lines.tif]

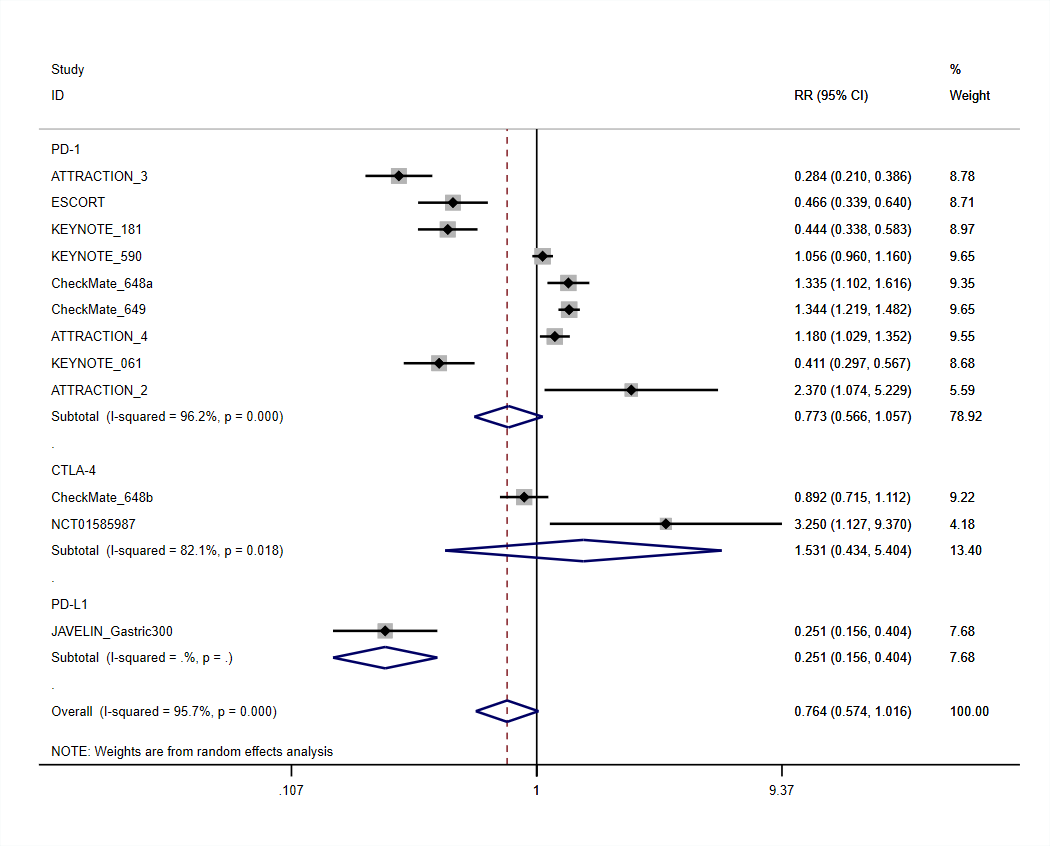

Supplement: Supplementary file 1 [file DataSheet_1.zip › Supplementary figures and tables/FIGURE S4C Forestplots for traditional pairwise meta-analysis for grade 3-5 trAEs subgrouped by ICIs drugs type.tif]

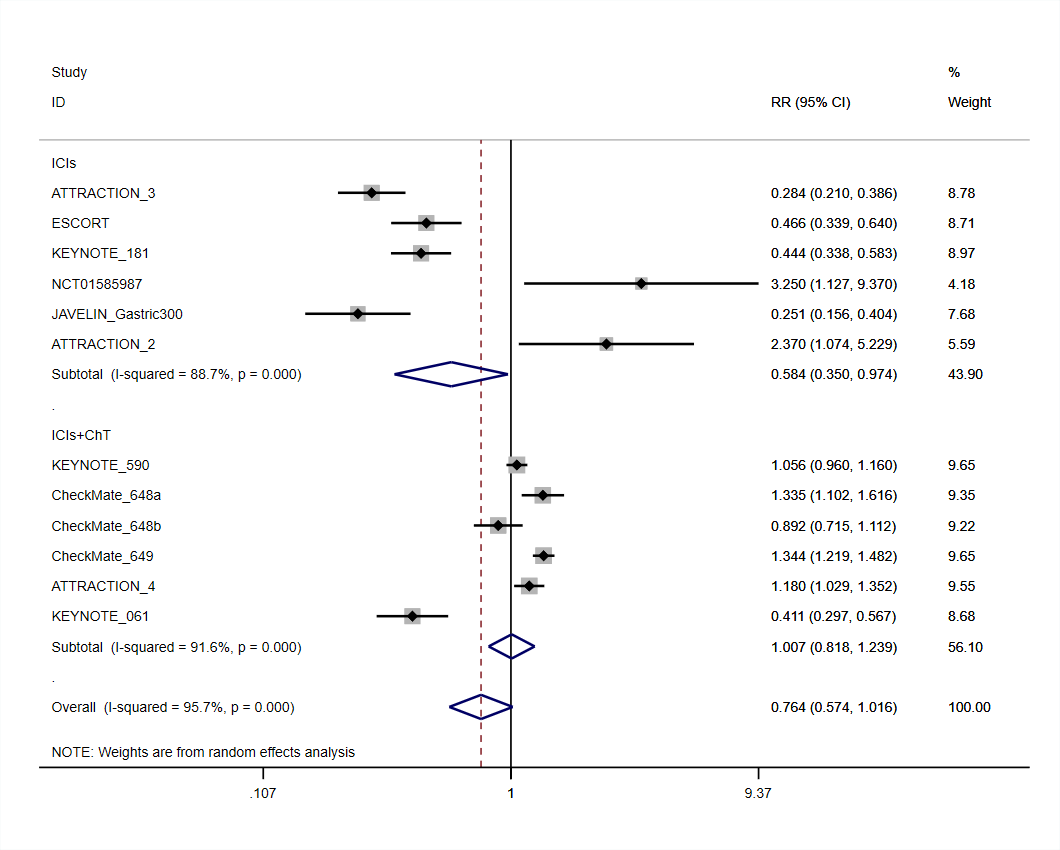

Supplement: Supplementary file 1 [file DataSheet_1.zip › Supplementary figures and tables/FIGURE S4D Forestplots for traditional pairwise meta-analysis for grade 3-5 trAEs subgrouped by treatment mode.tif]

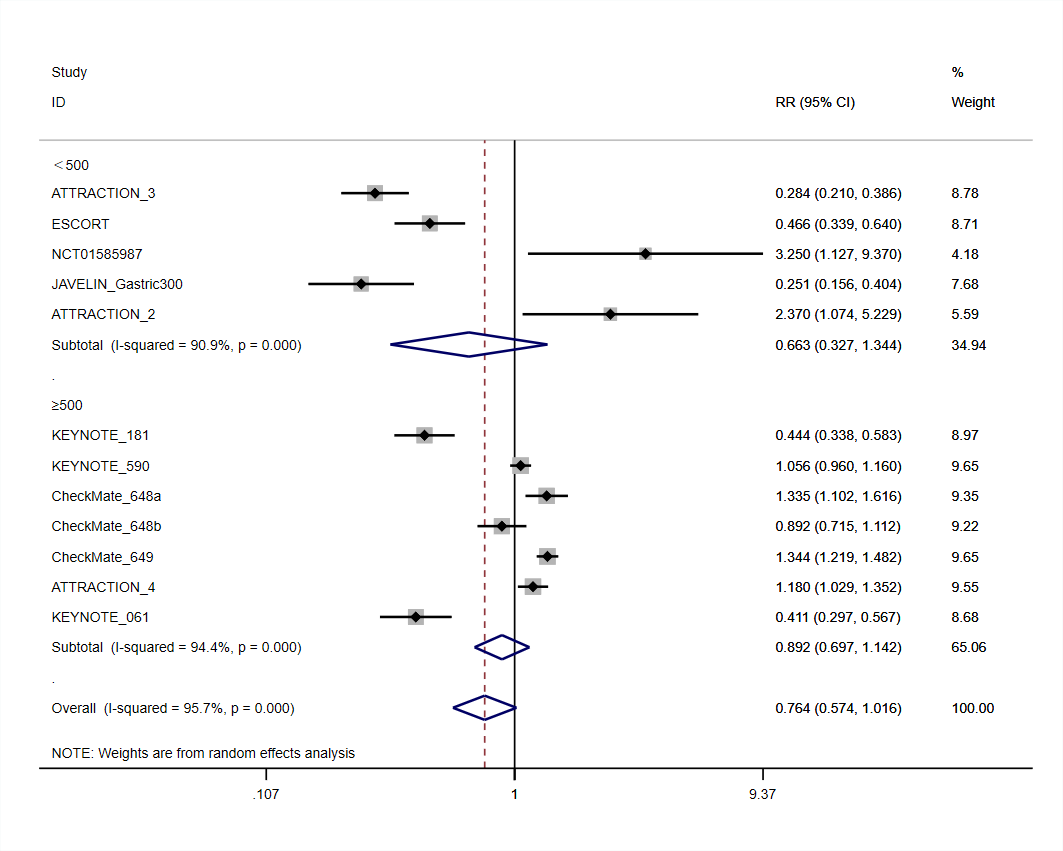

Supplement: Supplementary file 1 [file DataSheet_1.zip › Supplementary figures and tables/FIGURE S4E Forestplots for traditional pairwise meta-analysis for grade 3-5 trAEs subgrouped by sample size.tif]

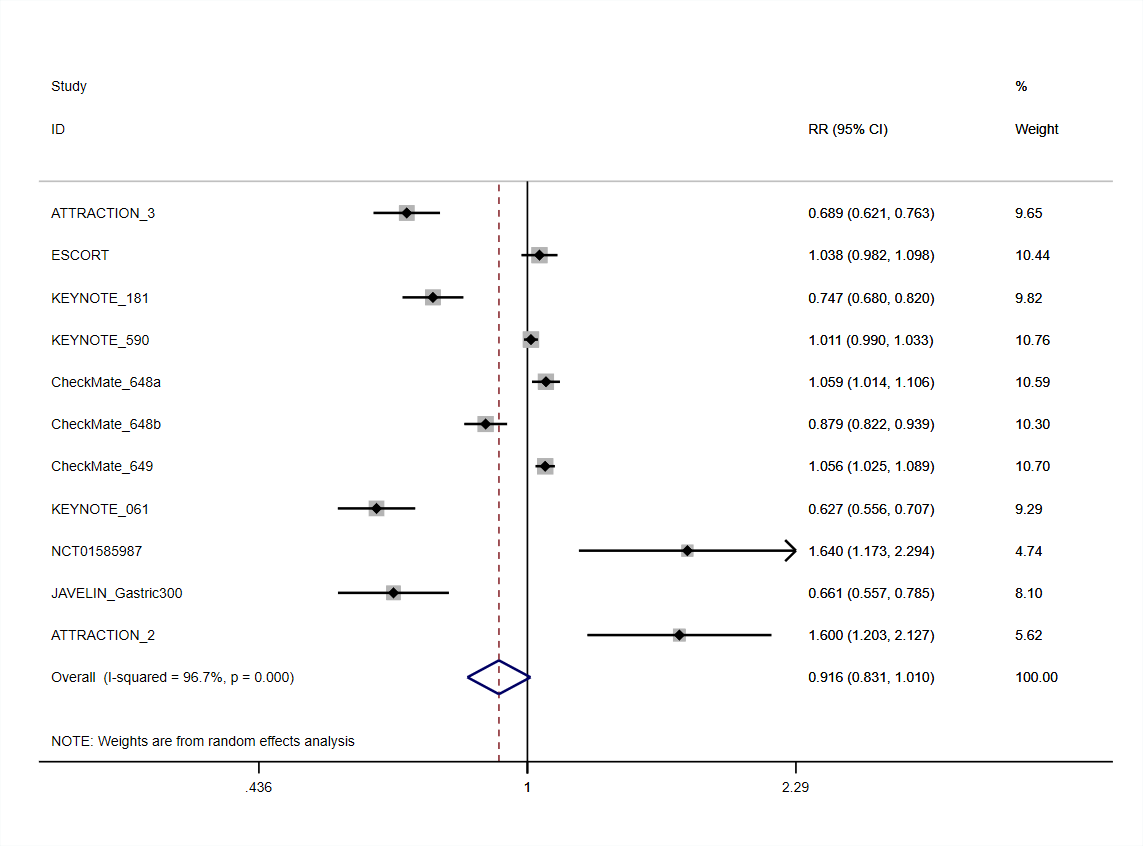

Supplement: Supplementary file 1 [file DataSheet_1.zip › Supplementary figures and tables/FIGURE S5A Subgroup forestplots for traditional pairwise meta-analysis for all grade trAEs.tif]

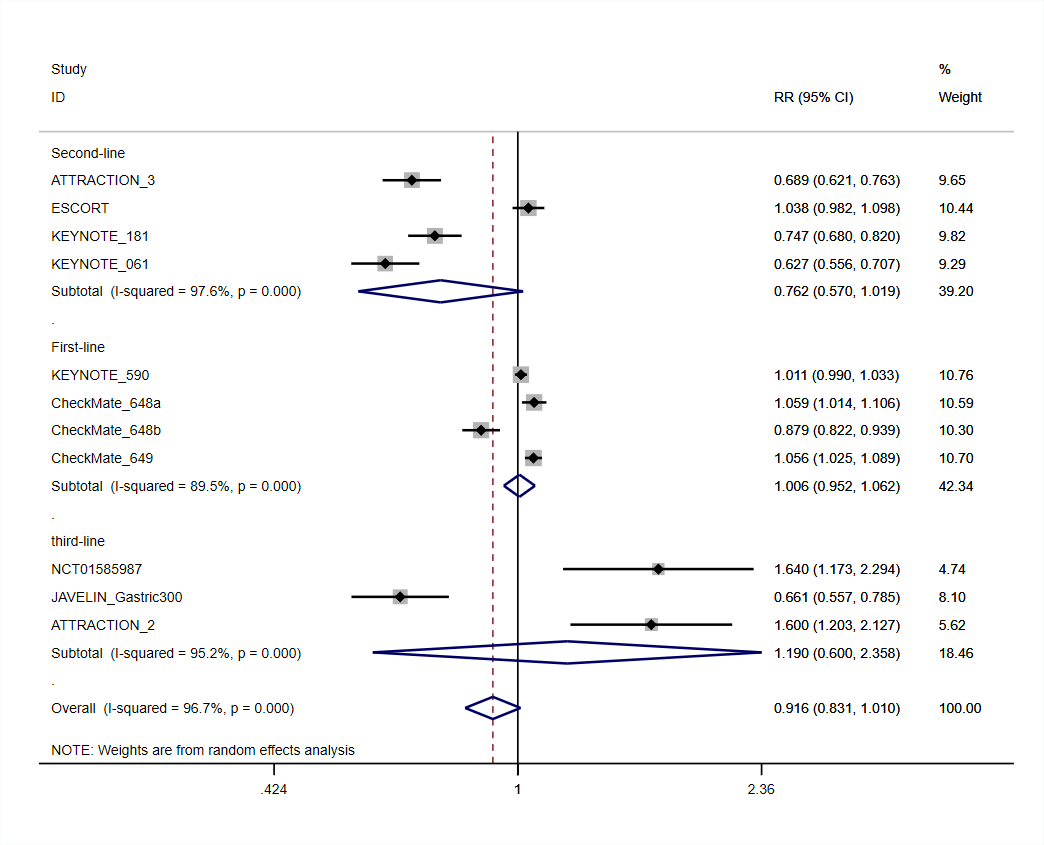

Supplement: Supplementary file 1 [file DataSheet_1.zip › Supplementary figures and tables/FIGURE S5B Forestplots for traditional pairwise meta-analysis for all grade trAEs subgrouped by treatment lines.tif]

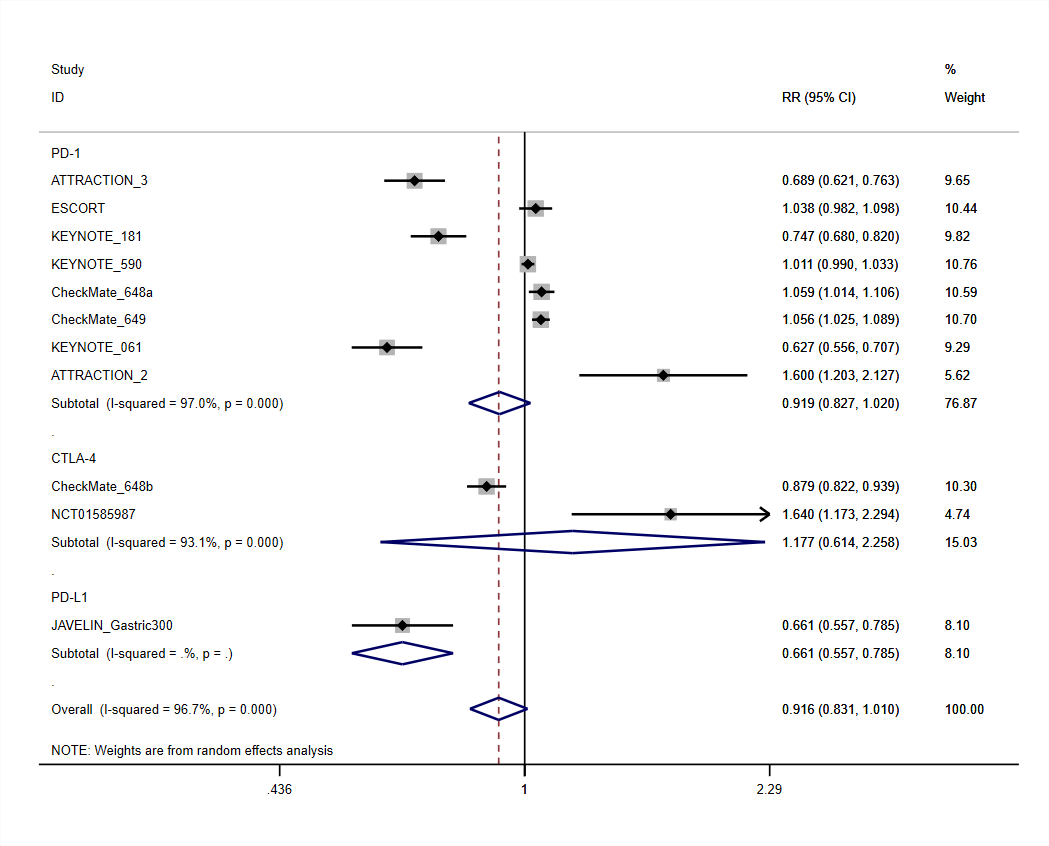

Supplement: Supplementary file 1 [file DataSheet_1.zip › Supplementary figures and tables/FIGURE S5C Forestplots for traditional pairwise meta-analysis for all grade trAEs subgrouped by ICIs drugs type.tif]

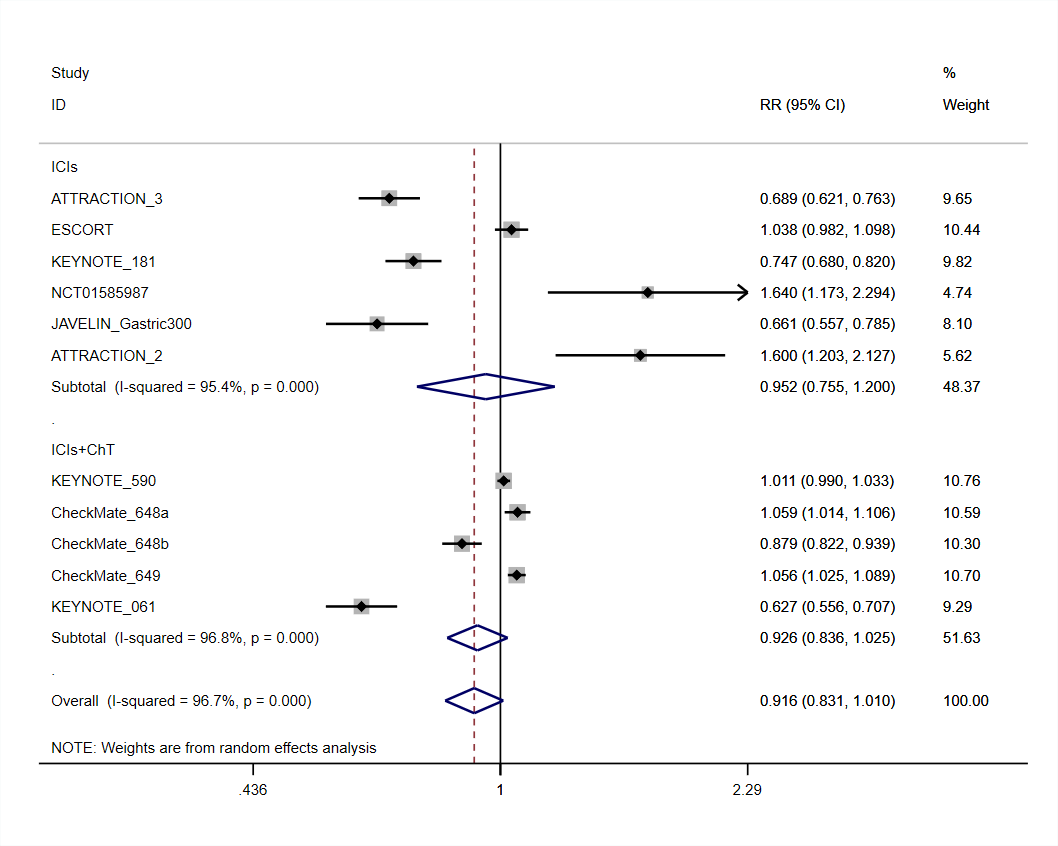

Supplement: Supplementary file 1 [file DataSheet_1.zip › Supplementary figures and tables/FIGURE S5D Forestplots for traditional pairwise meta-analysis for all grade trAEs subgrouped by treatment mode.tif]

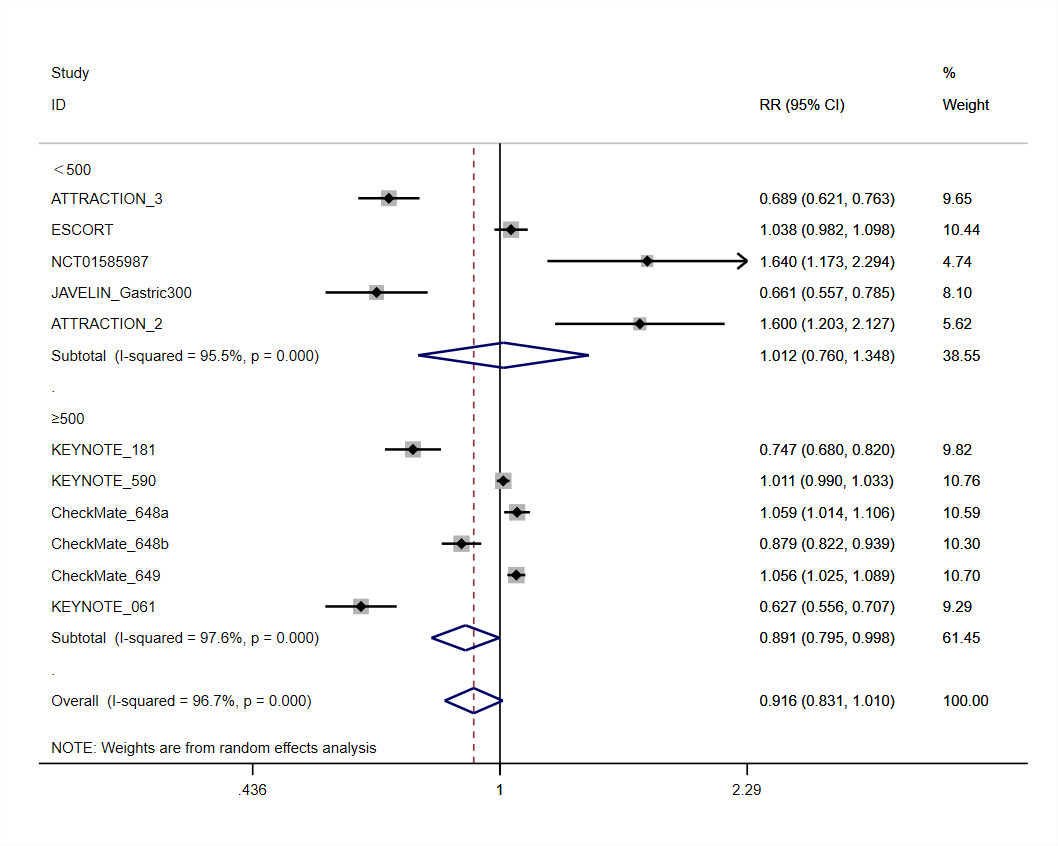

Supplement: Supplementary file 1 [file DataSheet_1.zip › Supplementary figures and tables/FIGURE S5E Forestplots for traditional pairwise meta-analysis for all grade trAEs subgrouped by sample size.tif]

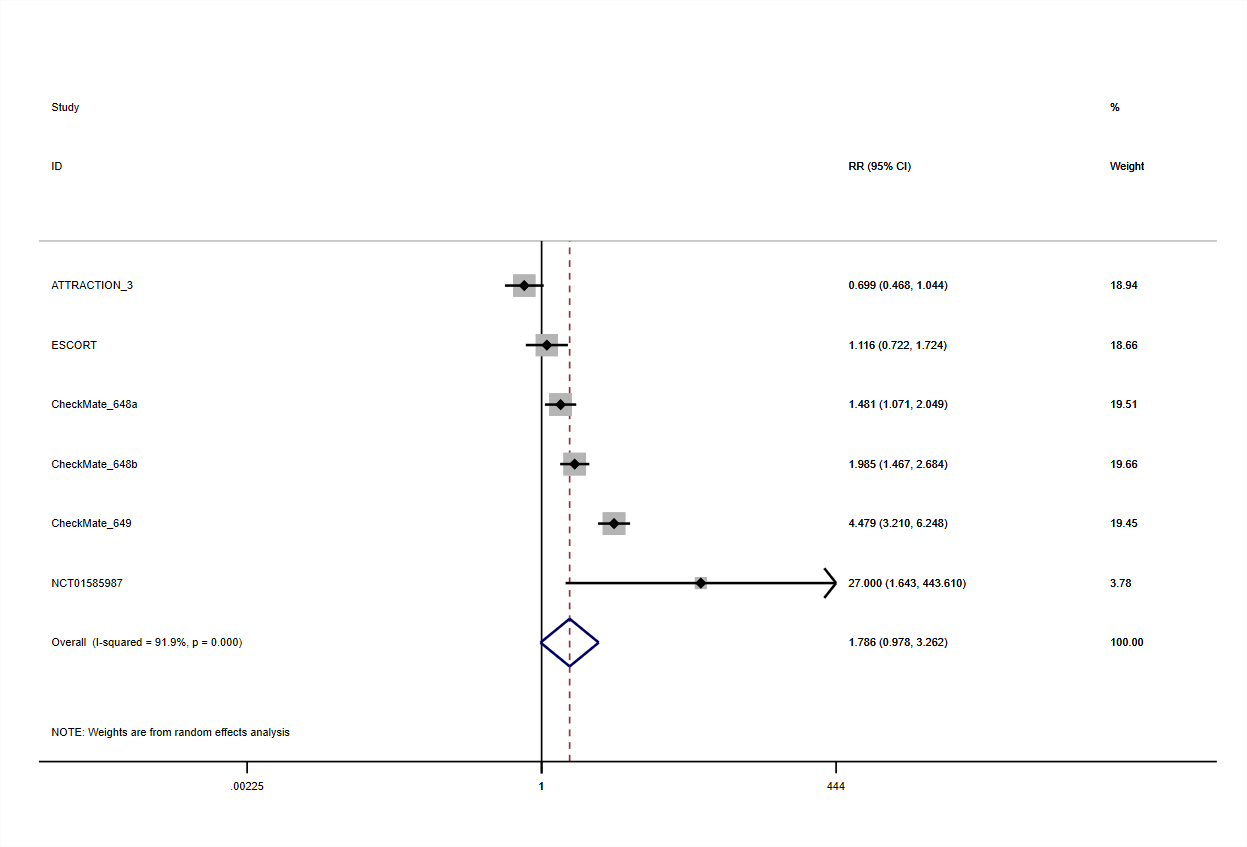

Supplement: Supplementary file 1 [file DataSheet_1.zip › Supplementary figures and tables/FIGURE S6A Forestplots for serious trAEs.tif]

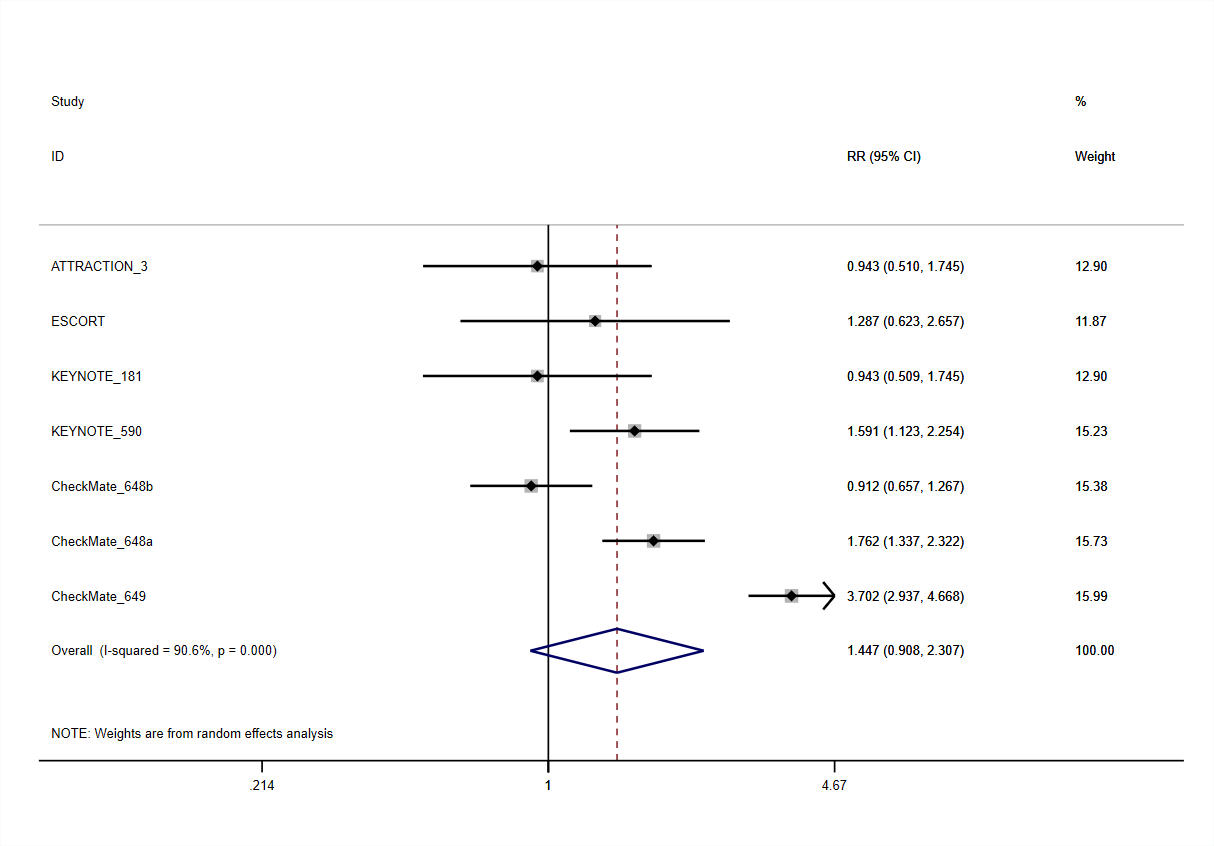

Supplement: Supplementary file 1 [file DataSheet_1.zip › Supplementary figures and tables/FIGURE S6B Forestplots for events leading to discontinuation.tif]

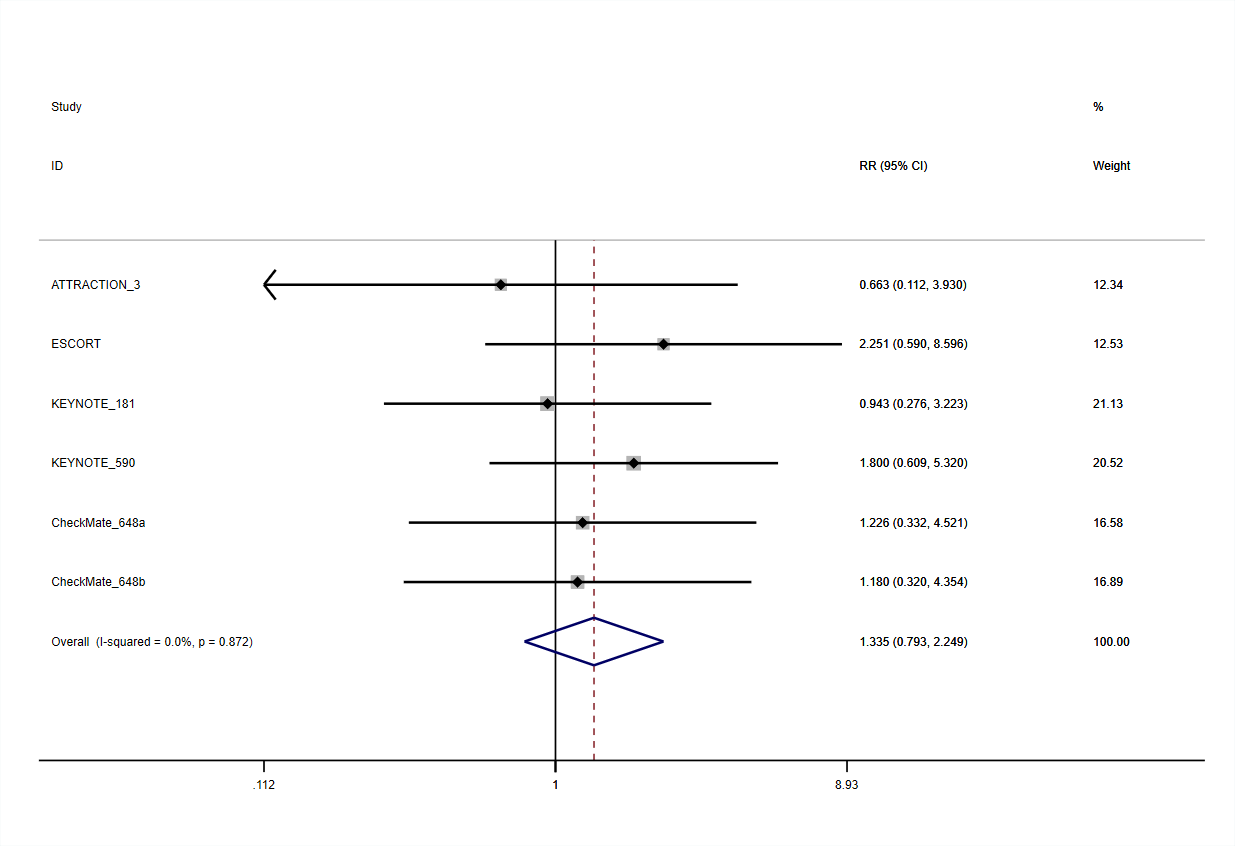

Supplement: Supplementary file 1 [file DataSheet_1.zip › Supplementary figures and tables/FIGURE S6C Forestplots for treatment-related death.tif]

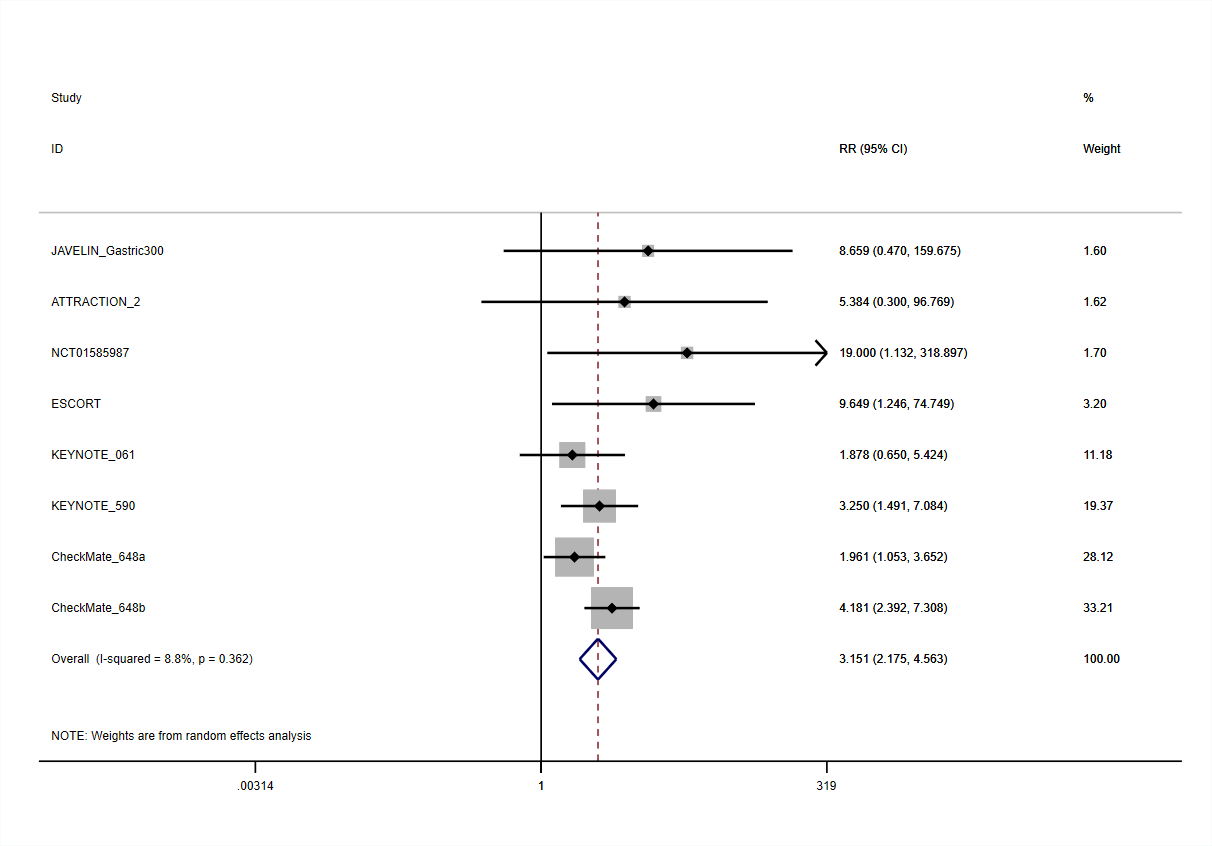

Supplement: Supplementary file 1 [file DataSheet_1.zip › Supplementary figures and tables/FIGURE S7A Subgroup forestplots for traditional pairwise meta-analysis for grade 3-5 irAEs.tif]

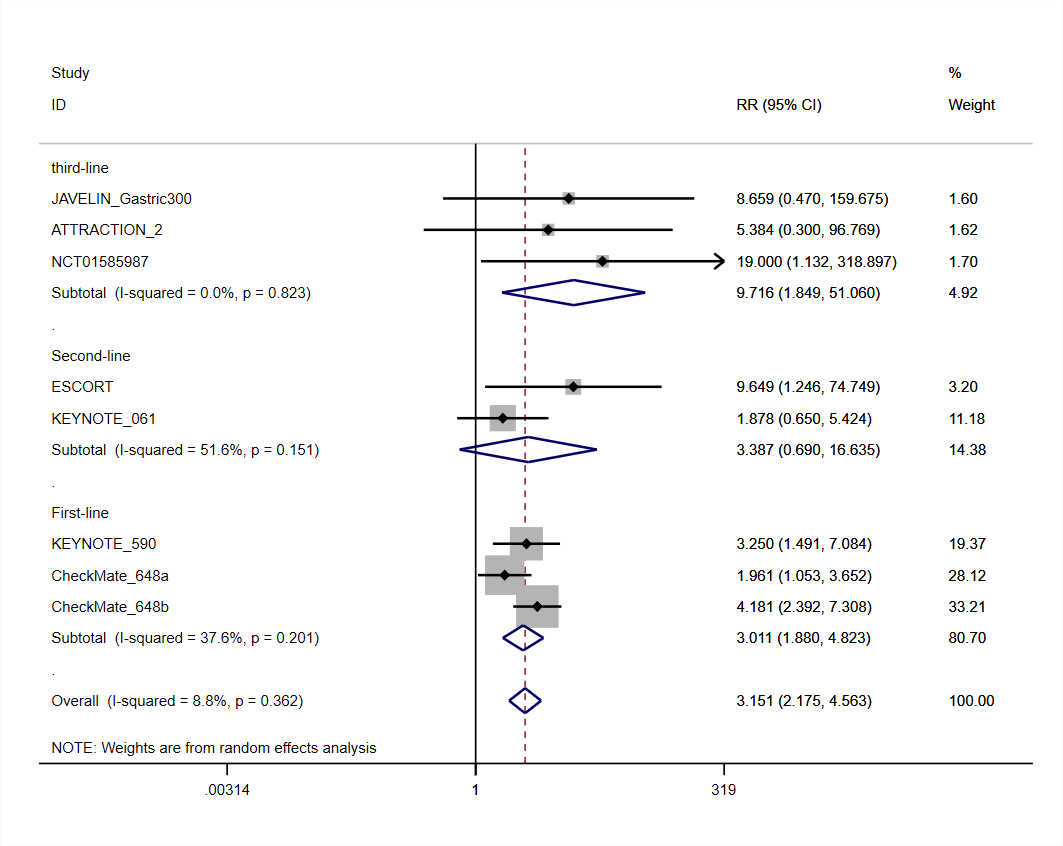

Supplement: Supplementary file 1 [file DataSheet_1.zip › Supplementary figures and tables/FIGURE S7B Forestplots for traditional pairwise meta-analysis for grade 3-5 irAEs subgrouped by treatment lines.tif]

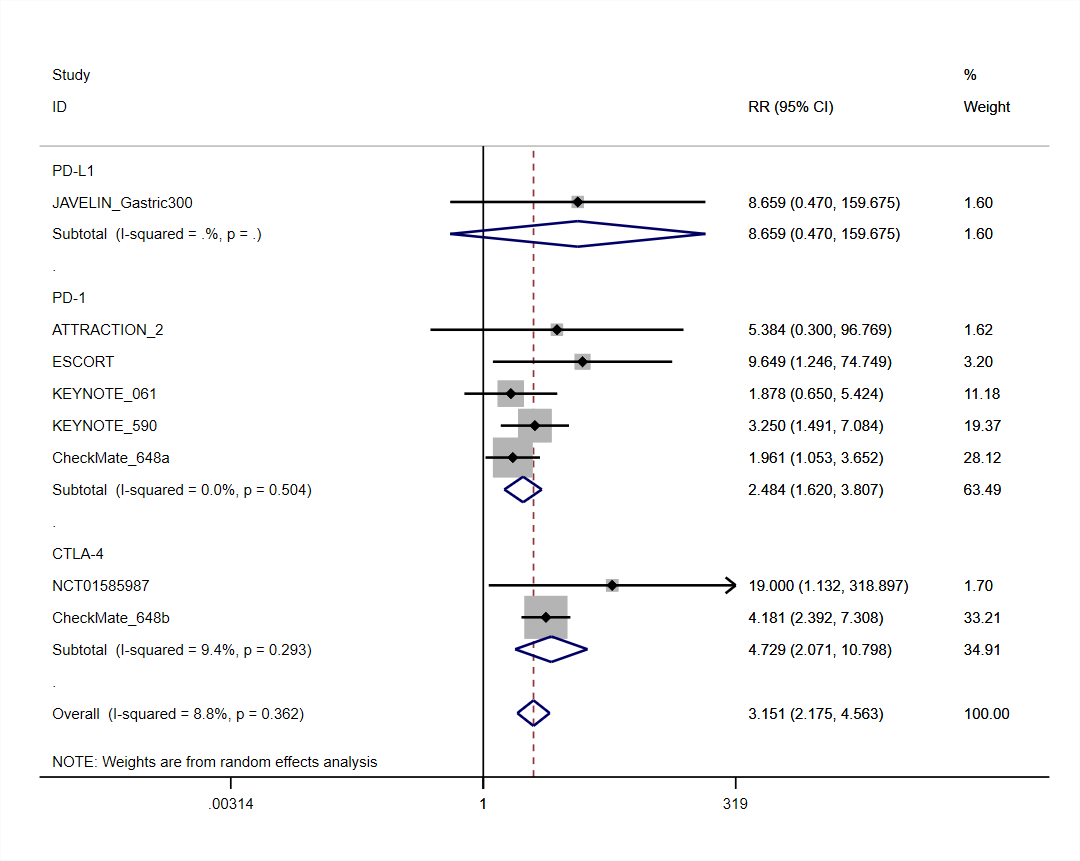

Supplement: Supplementary file 1 [file DataSheet_1.zip › Supplementary figures and tables/FIGURE S7C Forestplots for traditional pairwise meta-analysis for grade 3-5 irAEs subgrouped by ICIs drugs type.tif]

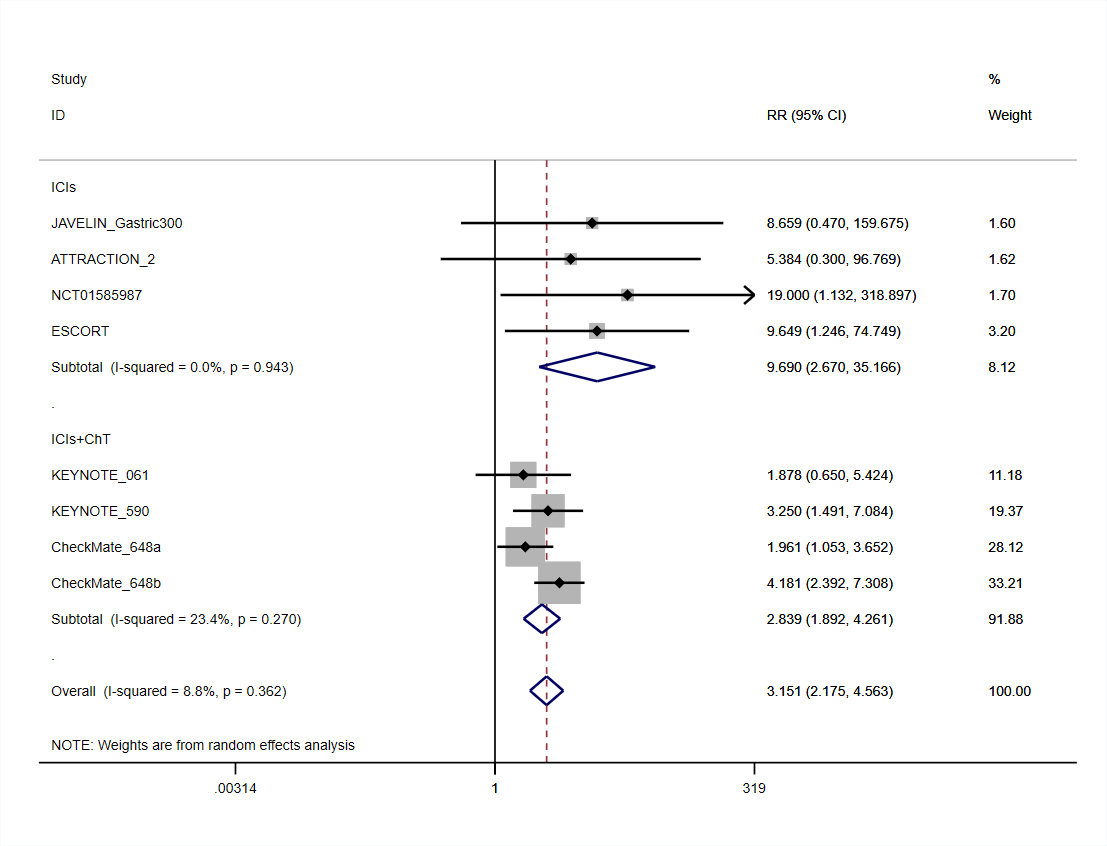

Supplement: Supplementary file 1 [file DataSheet_1.zip › Supplementary figures and tables/FIGURE S7D Forestplots for traditional pairwise meta-analysis for grade 3-5 irAEs subgrouped by treatment mode.tif]

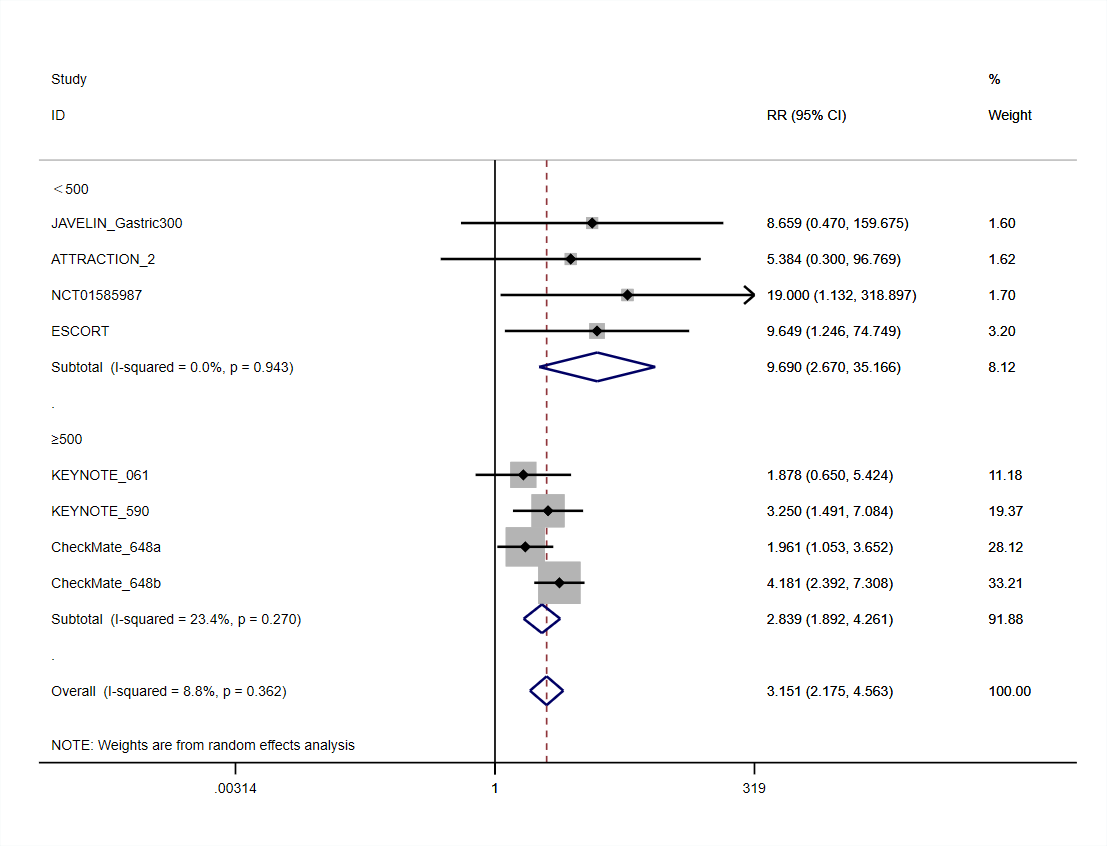

Supplement: Supplementary file 1 [file DataSheet_1.zip › Supplementary figures and tables/FIGURE S7E Forestplots for traditional pairwise meta-analysis for grade 3-5 irAEs subgrouped by sample size.tif]

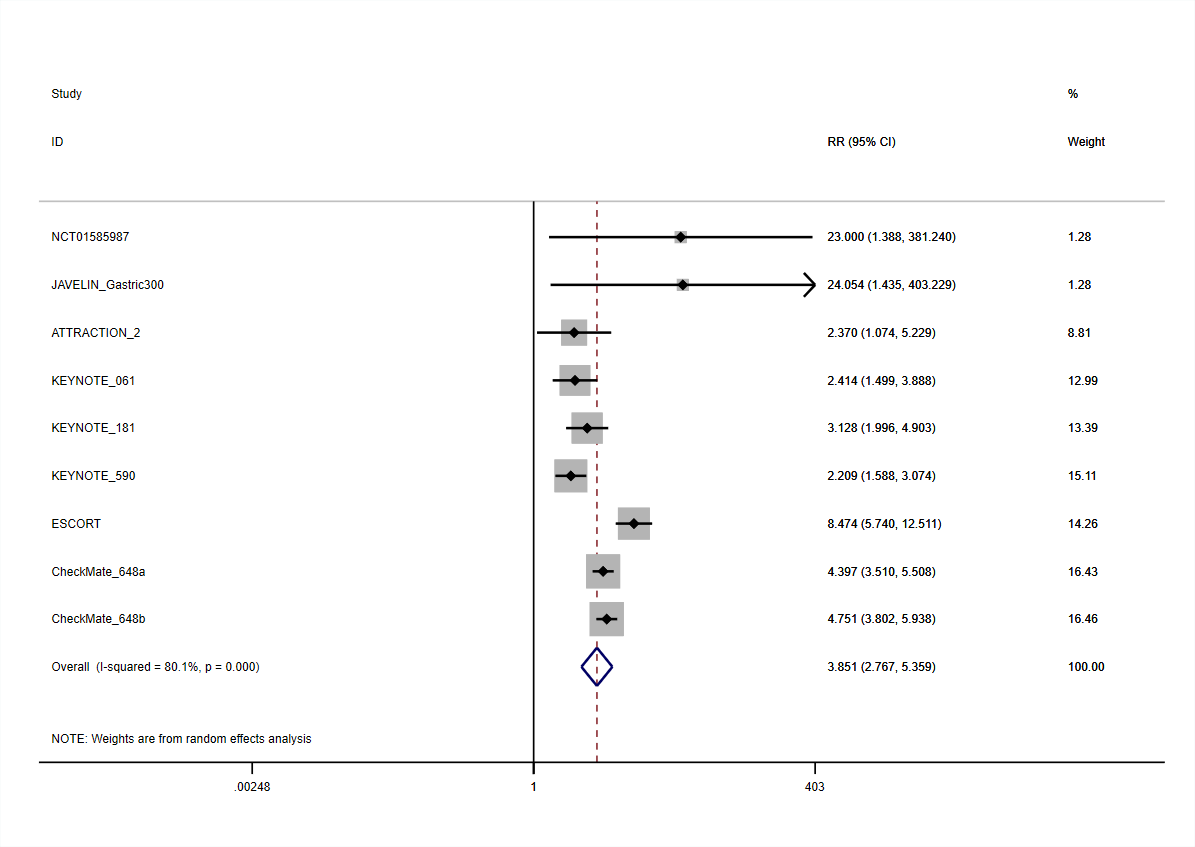

Supplement: Supplementary file 1 [file DataSheet_1.zip › Supplementary figures and tables/FIGURE S8A Subgroup forestplots for traditional pairwise meta-analysis for all grade irAEs.tif]

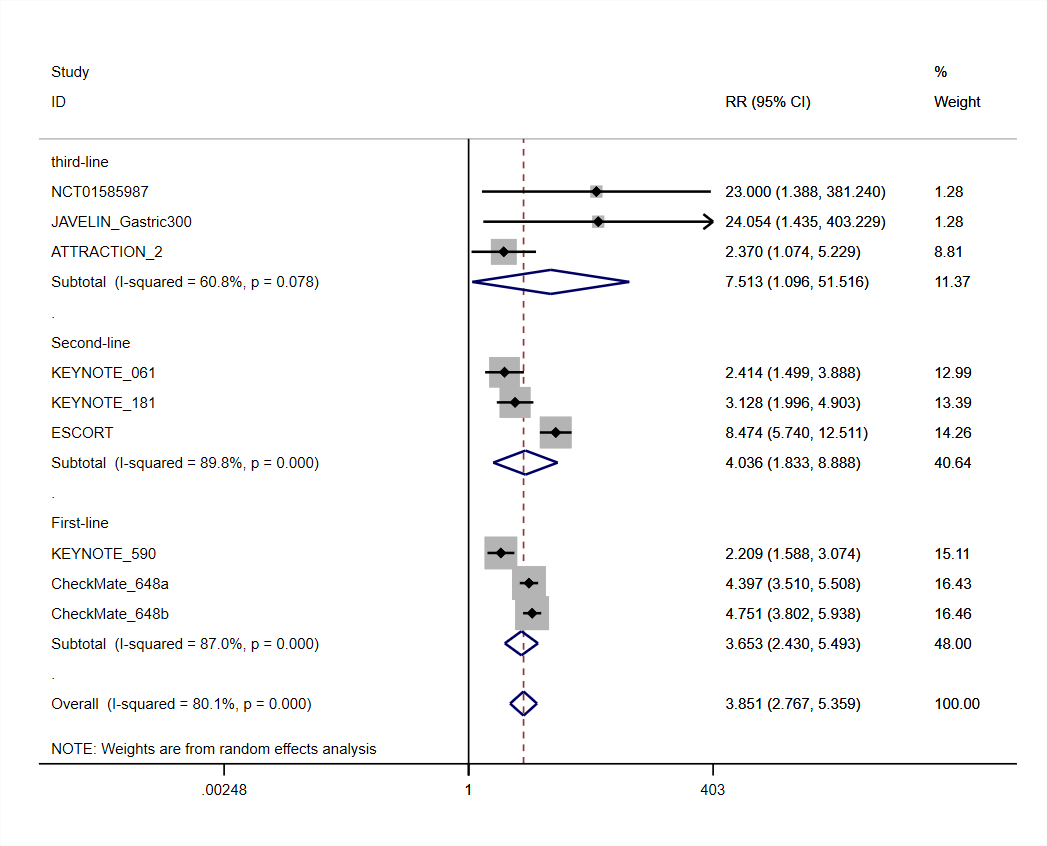

Supplement: Supplementary file 1 [file DataSheet_1.zip › Supplementary figures and tables/FIGURE S8B Forestplots for traditional pairwise meta-analysis for all grade irAEs subgrouped by treatment lines.tif]

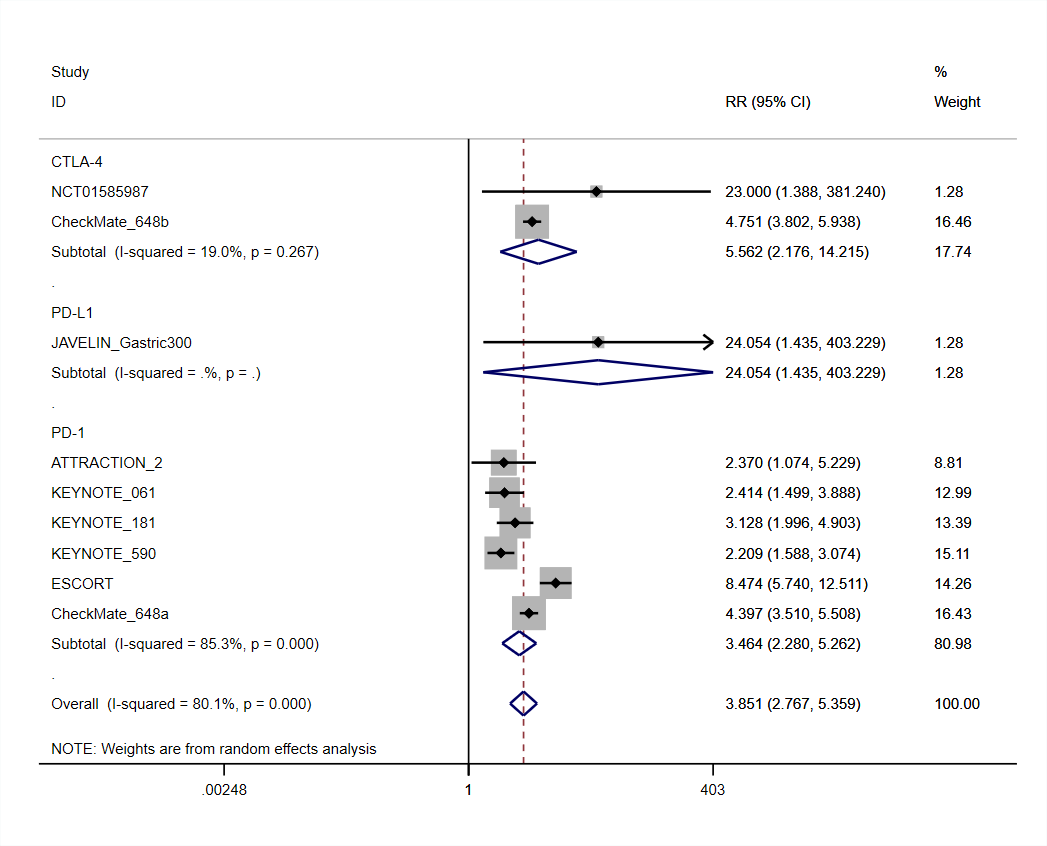

Supplement: Supplementary file 1 [file DataSheet_1.zip › Supplementary figures and tables/FIGURE S8C Forestplots for traditional pairwise meta-analysis for all grade irAEs subgrouped by ICIs drugs type.tif]

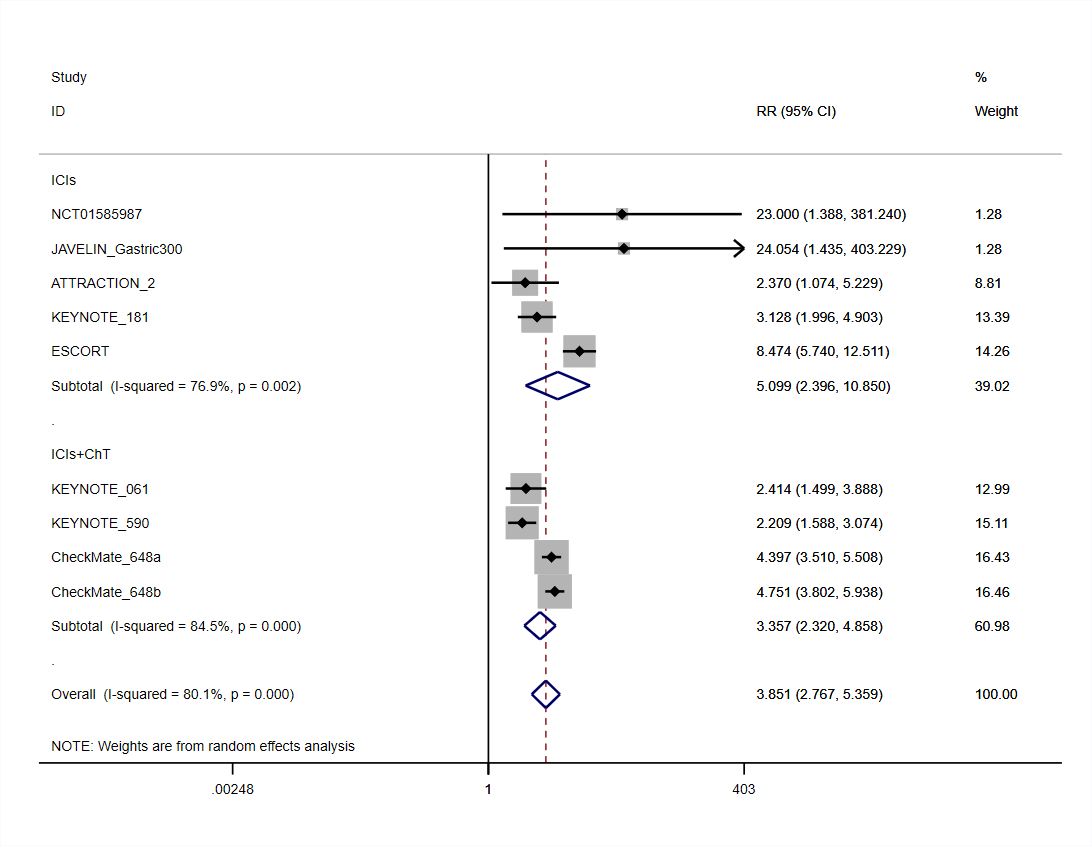

Supplement: Supplementary file 1 [file DataSheet_1.zip › Supplementary figures and tables/FIGURE S8D Forestplots for traditional pairwise meta-analysis for all grade irAEs subgrouped by treatment mode.tif]

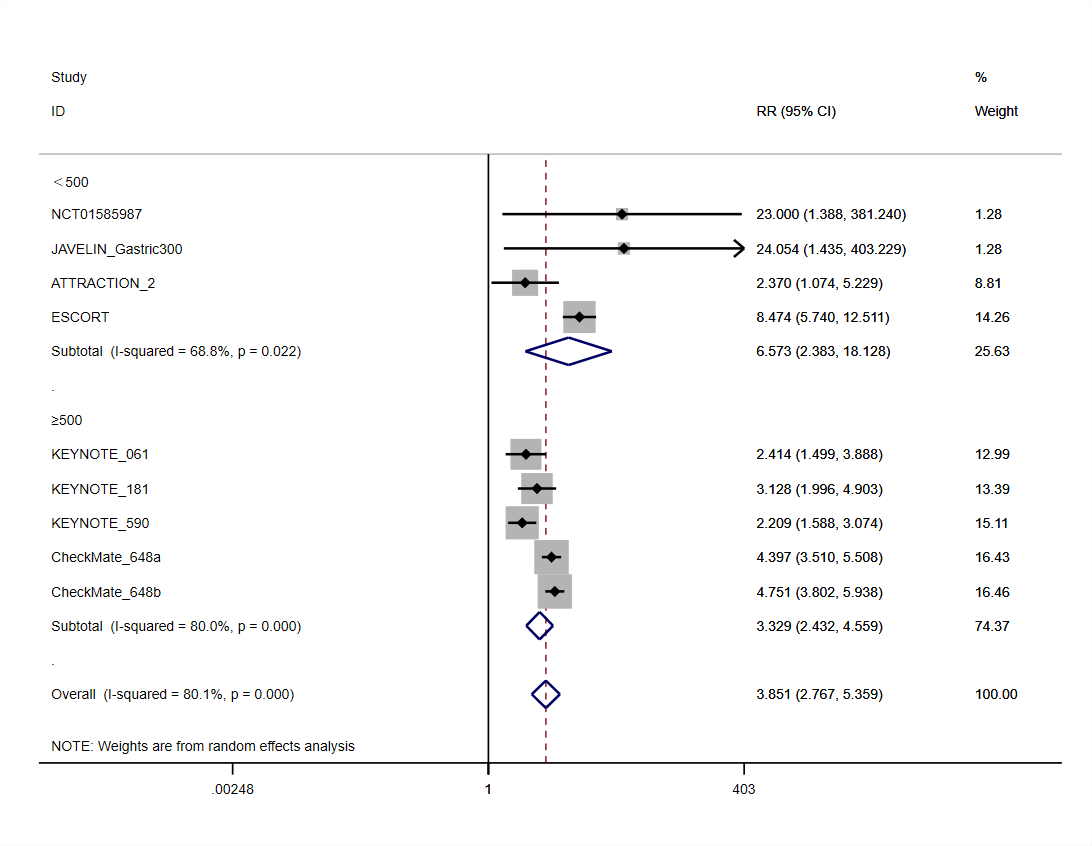

Supplement: Supplementary file 1 [file DataSheet_1.zip › Supplementary figures and tables/FIGURE S8E Forestplots for traditional pairwise meta-analysis for all grade irAEs subgrouped by sample size.tif]
